# Supplementary material for: Impact and cost-effectiveness of the community-led AMETHIST intervention among female sex workers in Zimbabwe
Source: Nat Commun. 2025 Dec 13;17:241. doi: 10.1038/s41467-025-66927-x (PMC12783726; doi:10.1038/s41467-025-66927-x)
Supplement: Supplementary file 1 — Supplementary Information [file 41467_2025_66927_MOESM1_ESM.pdf]

Appendix

Impact on general population HIV incidence and cost-effectiveness of the enhanced AMETHIST intervention for sex workers in the context of an existing sex worker programme in Zimbabwe

Lead author: Loveleen Bansi-Matharu (l.bansi-matharu@ucl.ac.uk)

Contents

Table A1: Calibration with Zimbabwe for adults aged 15+ adults	Page 2
Table A2: Breakdown of key costs for each scenario	Page 3
Calibration figures	Page 4
Model details	Page 13
Distribution of parameters	Page 99
Sexual behaviour	*Page 99*
HIV-testing	*Page 102*
PrEP	*Page 103*
ART	*Page 111*
Sex workers	*Page 116*

Table A1: Calibration with Zimbabwe for adults aged 15+ adults

(FSW modelled and observed comparison is shown in Table 1 of the main text)

	Modelled Mean (90% range)<br><br>(estimates are shown for the same year as the observed data)	Observed
Number of adults living with HIV	1,215,537 (1102938,1435784)	1 200 000 (UNAIDS 2023[1])
Percentage of PLHIV diagnosed	87.4% (81.9, 94.1)	86.8% (PHIA 2020[2])
Of those diagnosed, percentage of people on ART	96.3% (92.5, 98.6)	97.0% (PHIA 2020)
Of those on ART, percentage of people virally suppressed	88.4% (80.9, 94.3)	90.3% (PHIA 2020)
Number on ART	1,097,965 (1003414, 1249138)	1,189,793 (UNAIDS 2023)
Prevalence	12.1% (10.3, 13.7)	11.8 % (PHIA 2020)
Incidence (per 100 person years)	0.5 (0.3, 0.9)	0.5 (PHIA 2020)
	0.4 (0.2, 0.7)	0.2 (UNAIDS 2023)

[1] Zimbabwe | UNAIDS Accessed May 2025. Note that UNAIDS data are modelled estimates.

[2] Ministry of Health and Child Care (MOHCC) Z. Zimbabwe Population-Based HIV Impact Assessment (ZIMPHIA) 2020: Full Report. Harare, MOHCC.; Dec 2021.

Table A2: Breakdown of key costs for each scenario

	**Continuation of KP programme from 2024 onwards**	**KP + AMETHIST from 2024 onwards**	**Discontinuation of KP programme from 2024 onwards**
Costs	Mean across runs (95% CI)	Mean across runs (95% CI)	Mean across runs (95% CI)
**Antiretroviral (ART)**	39.9 (38.1, 41.6)	38.5 (36.8, 40.2)	44.6 (42.5, 46.7)
**PrEP**	8.8 (7.0, 10.6)	8.8 (7.1, 10.5)	10.1 (8.3, 11.9)
**Non ART clinical care for HIV**	15.3 (14.8, 15.8)	15.1 (14.6, 15.6)	17.6 (16.3, 19.0)
**Viral load**	7.7 (6.9, 8.5)	7.5 (6.7, 8.2)	8.4 (7.6, 9.3)
**Visit**	27.2 (25.7, 28.7)	26.2 (24.7, 27.7)	30.5 (28.7, 32.2)
**Testing**	17.1 (15.8, 18.5)	17.3 (15.9, 18.6)	17.6 (16.3, 19.0)
**Circumcision**	6.2 (5.7, 6.6)	6.2 (5.7, 6.6)	6.2 (5.7, 6.6)
**Condom distribution**	30.3 (30.2, 30.4)	30.1 (30.2, 30.3)	30.1 (30.0, 30.2)
**SW program**	4.0 (3.5, 4.4)	4.8 (4.2, 5.3)	0

Calibration to Zimbabwe

Key HIV epidemic outputs

(Modelled data are means across runs)

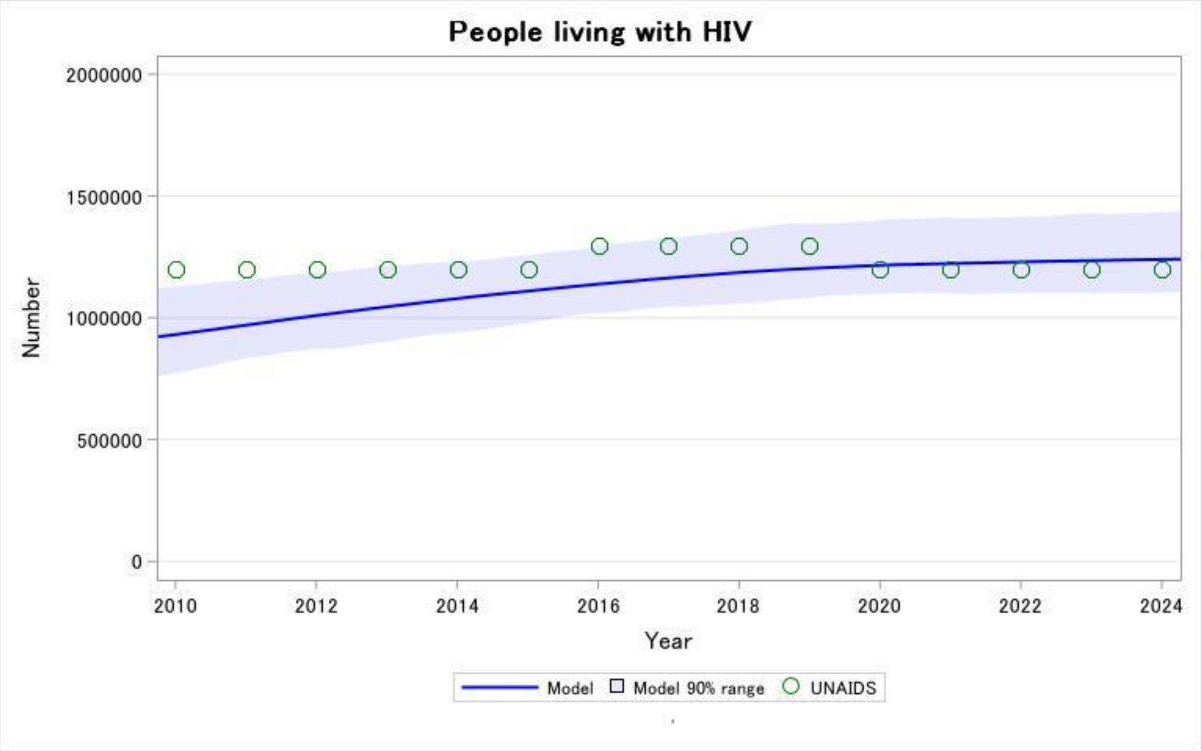

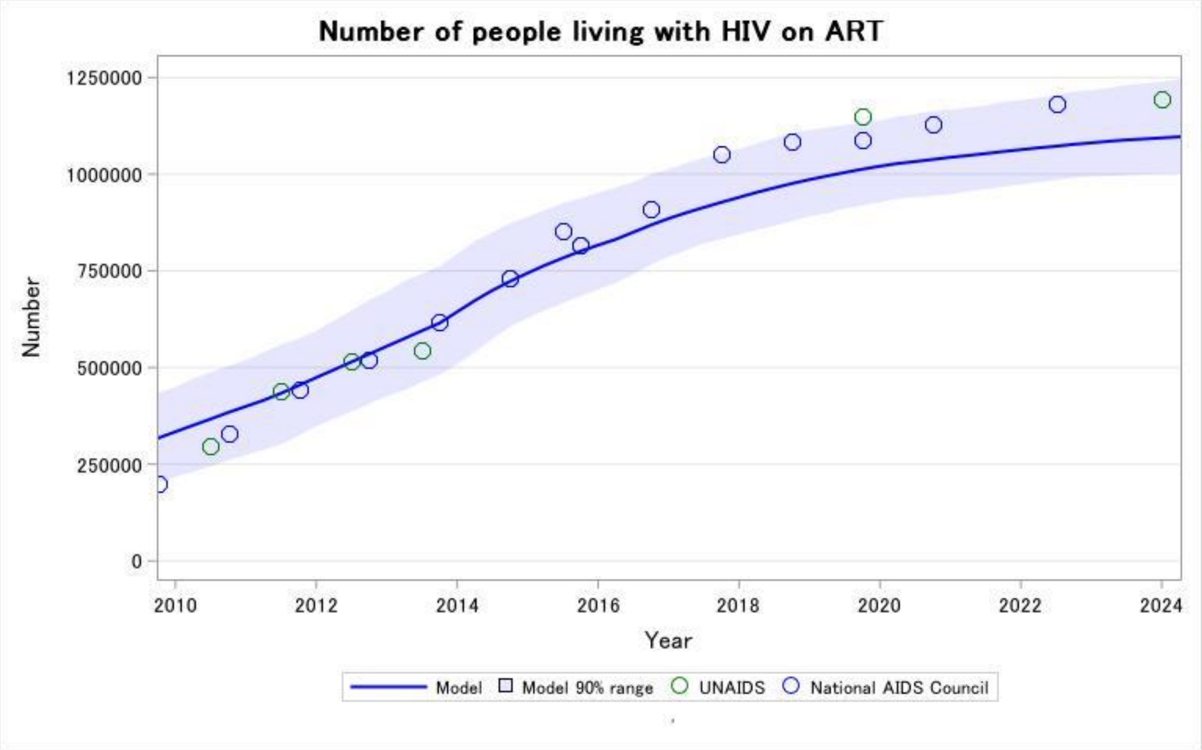

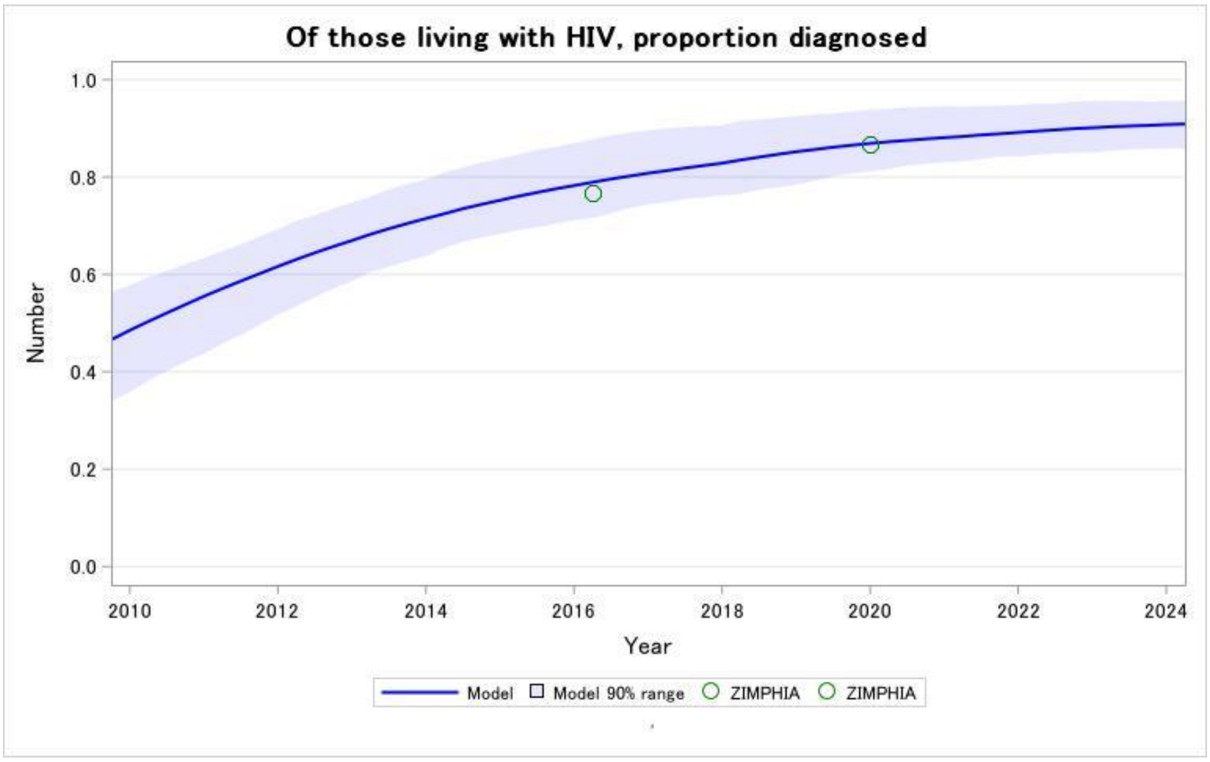

Of those living with HIV, proportion diagnosed
Number
1.0
0.8
0.6
0.4
0.2
0.0
2010
2012
2014
2016
2018
2020
2022
2024
Year
Model
Model 90% range
ZIMPHIA
ZIMPHIA

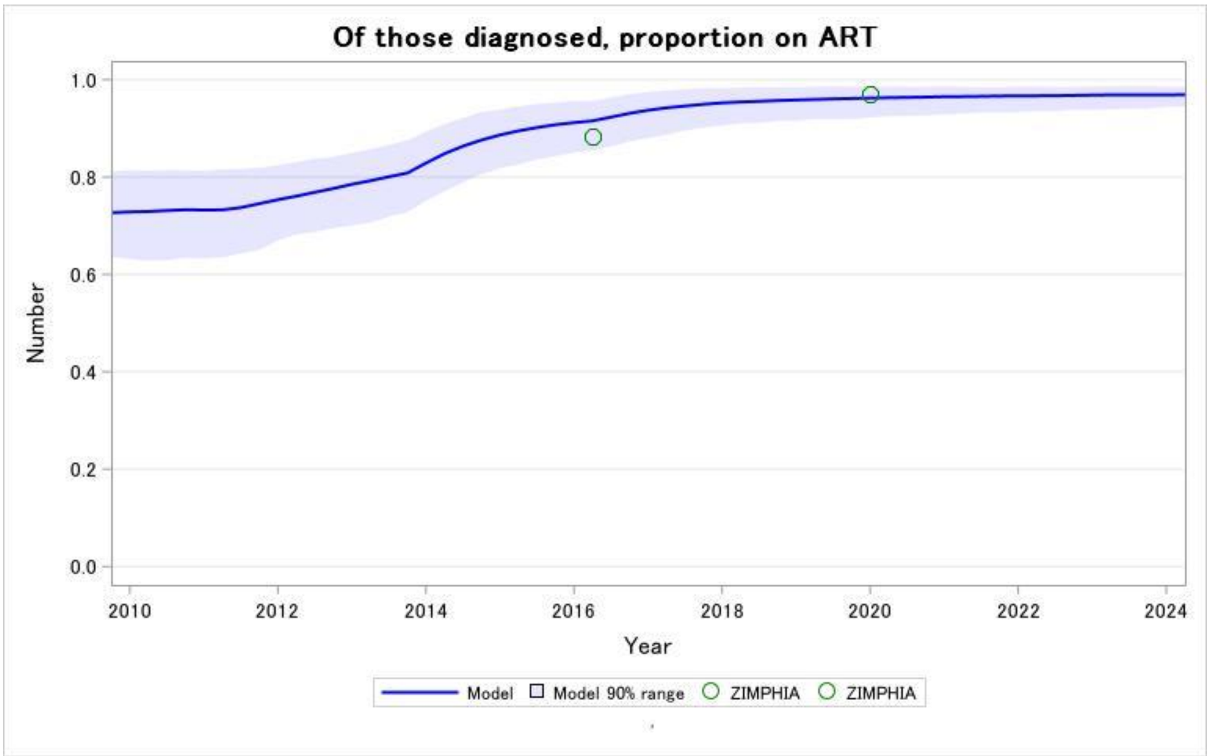

Of those diagnosed, proportion on ART
Number
1.0
0.8
0.6
0.4
0.2
0.0
2010
2012
2014
2016
2018
2020
2022
2024
Year
Model
Model 90% range
ZIMPHIA
ZIMPHIA

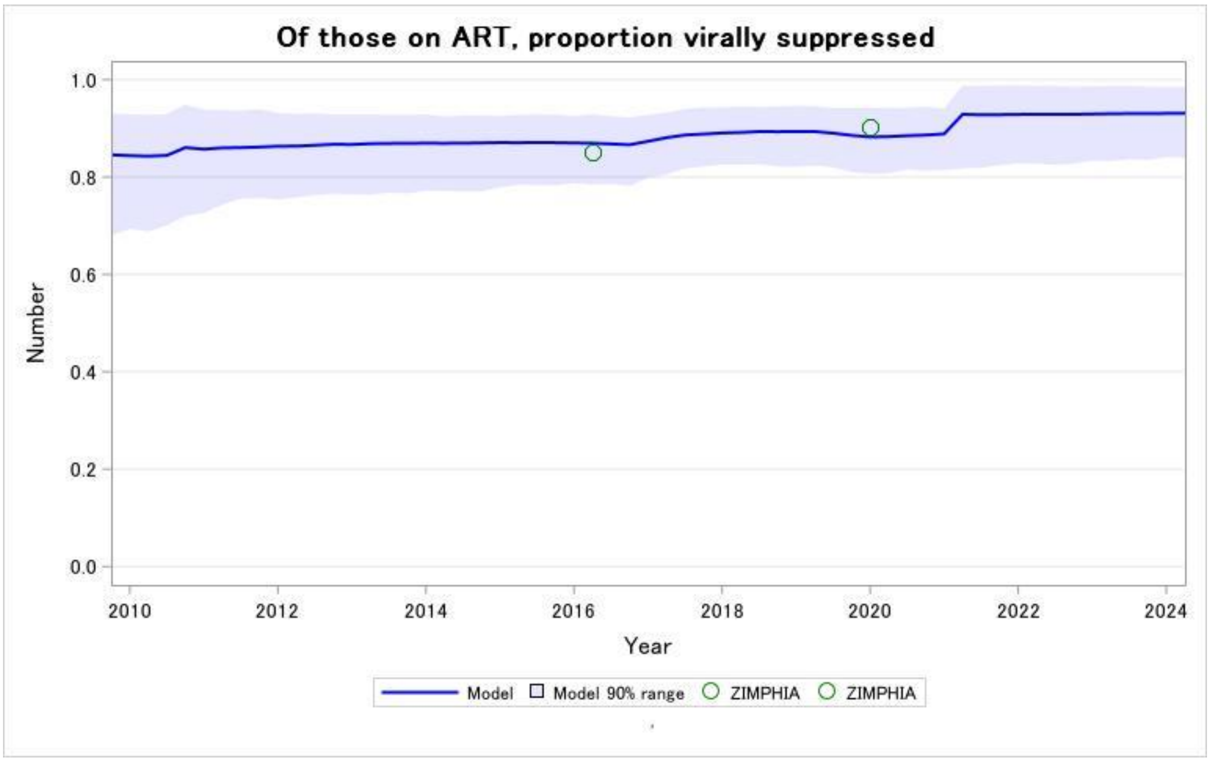
Of those on ART, proportion virally suppressed
Number
1.0
0.8
0.6
0.4
0.2
0.0
2010
2012
2014
2016
2018
2020
2022
2024
Year
Model
Model 90% range
ZIMPHIA
ZIMPHIA

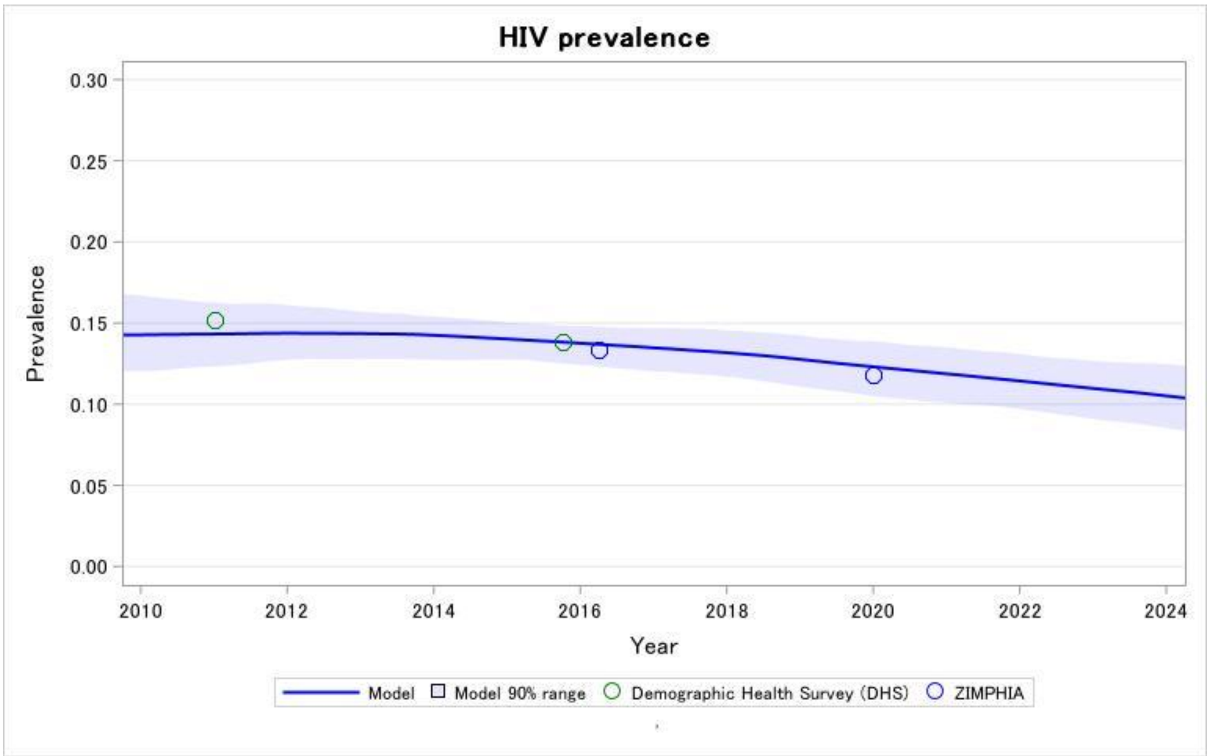
HIV prevalence
Prevalence
0.30
0.25
0.20
0.15
0.10
0.05
0.00
2010
2012
2014
2016
2018
2020
2022
2024
Year
Model
Model 90% range
Demographic Health Survey (DHS)
ZIMPHIA

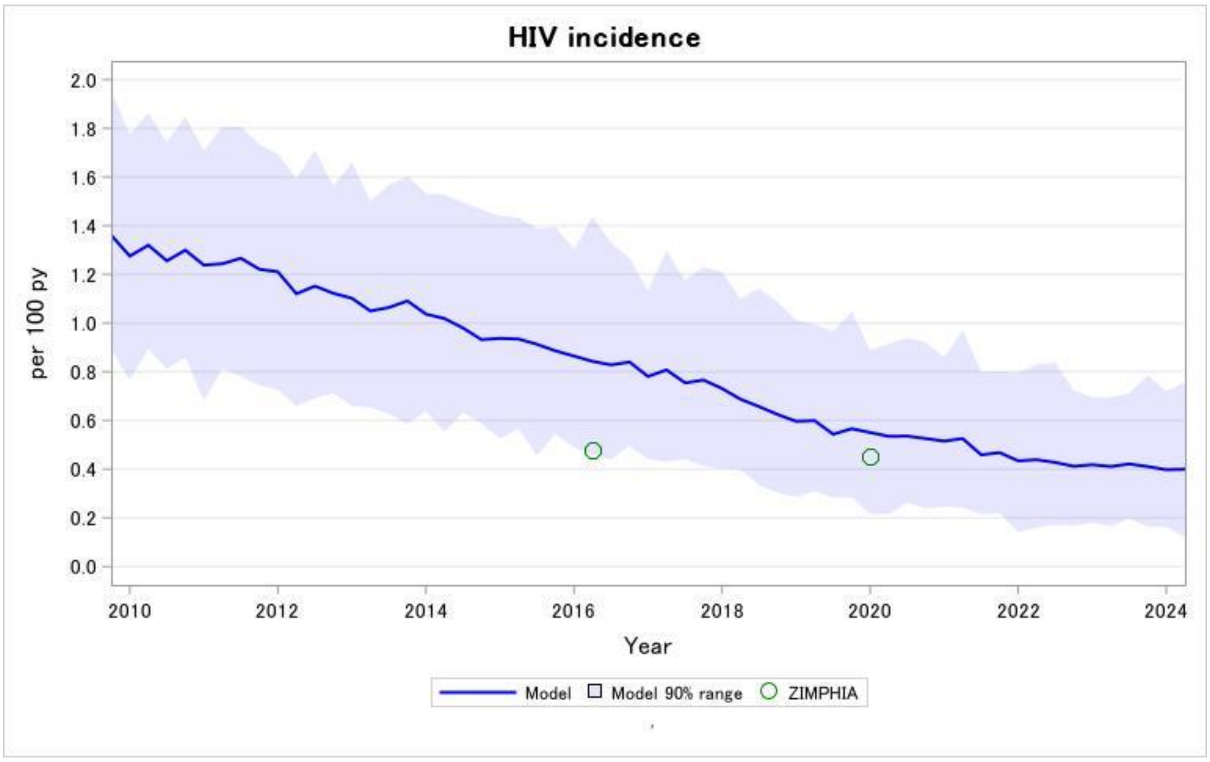
HIV incidence
per 100 py
2.0
1.8
1.6
1.4
1.2
1.0
0.8
0.6
0.4
0.2
0.0
2010
2012
2014
2016
2018
2020
2022
2024
Year
Model
Model 90% range
ZIMPHIA

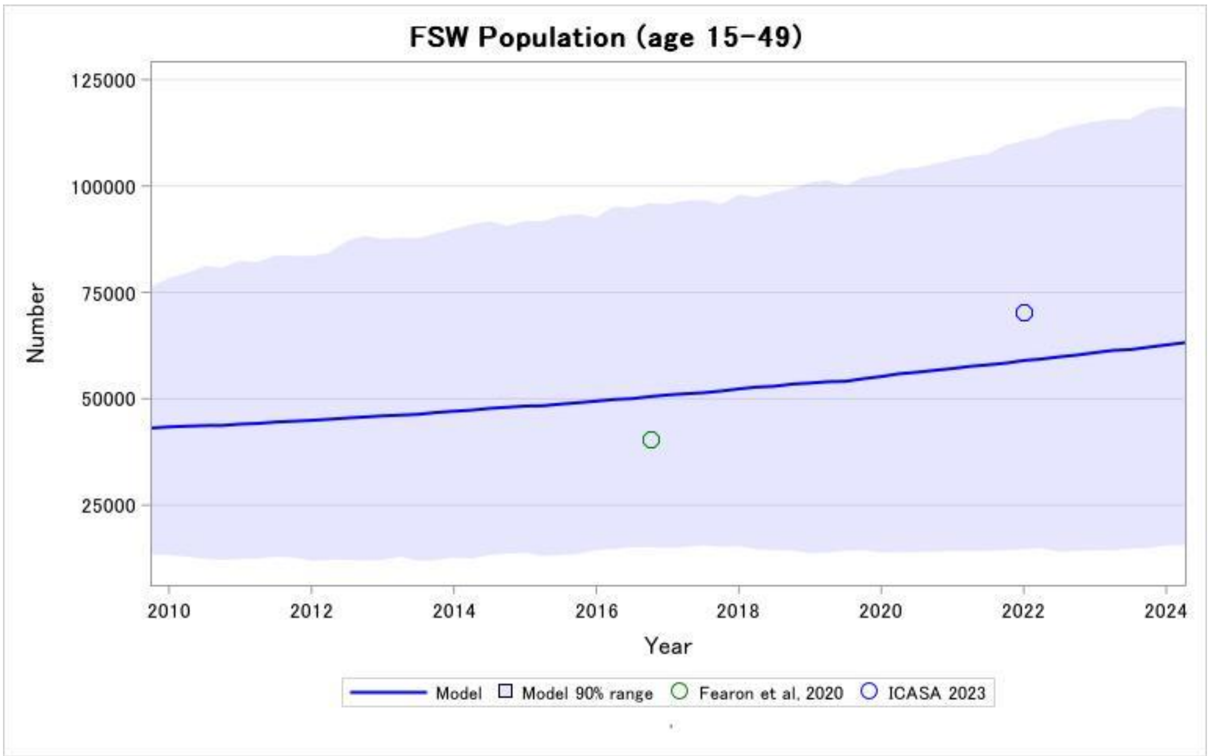
FSW Population (age 15-49)
Number
125000
100000
75000
50000
25000
2010
2012
2014
2016
2018
2020
2022
2024
Year
Model
Model 90% range
Fearon et al, 2020
ICASA 2023

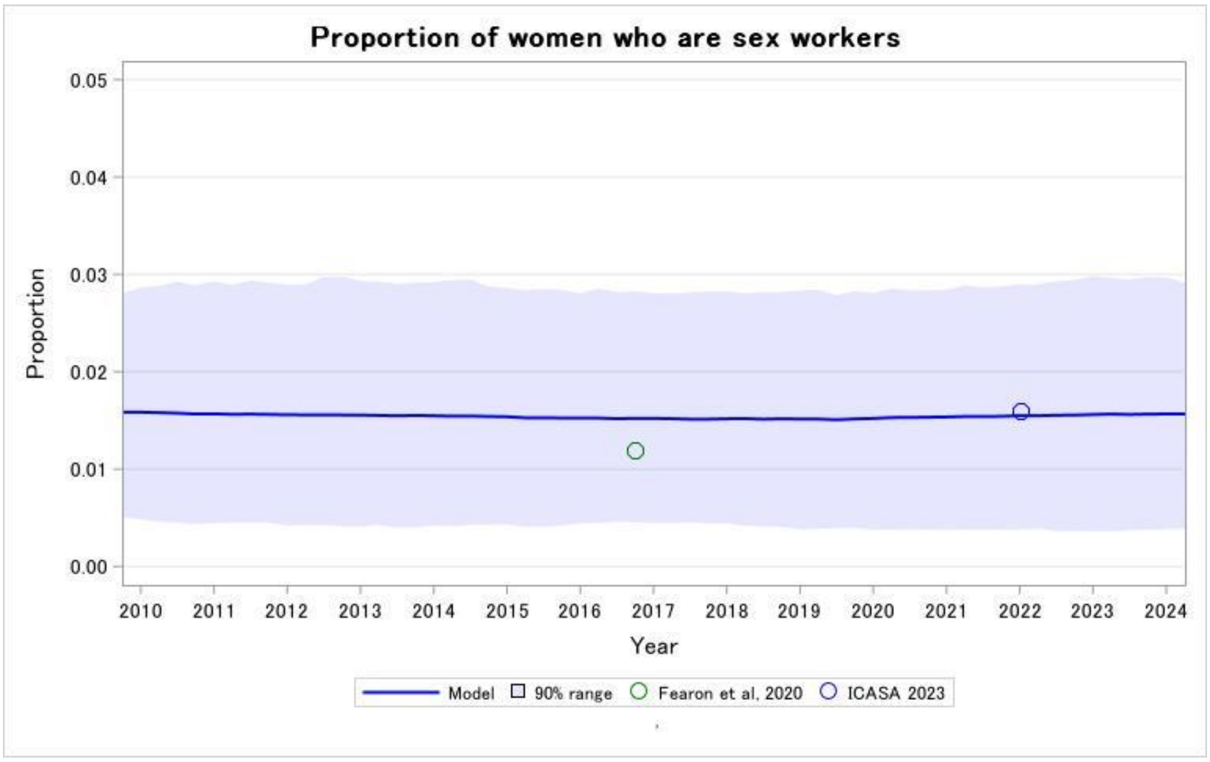

Proportion of women who are sex workers
Proportion
0.05
0.04
0.03
0.02
0.01
0.00
2010
2011
2012
2013
2014
2015
2016
2017
2018
2019
2020
2021
2022
2023
2024
Year
Model
90% range
Fearon et al, 2020
ICASA 2023

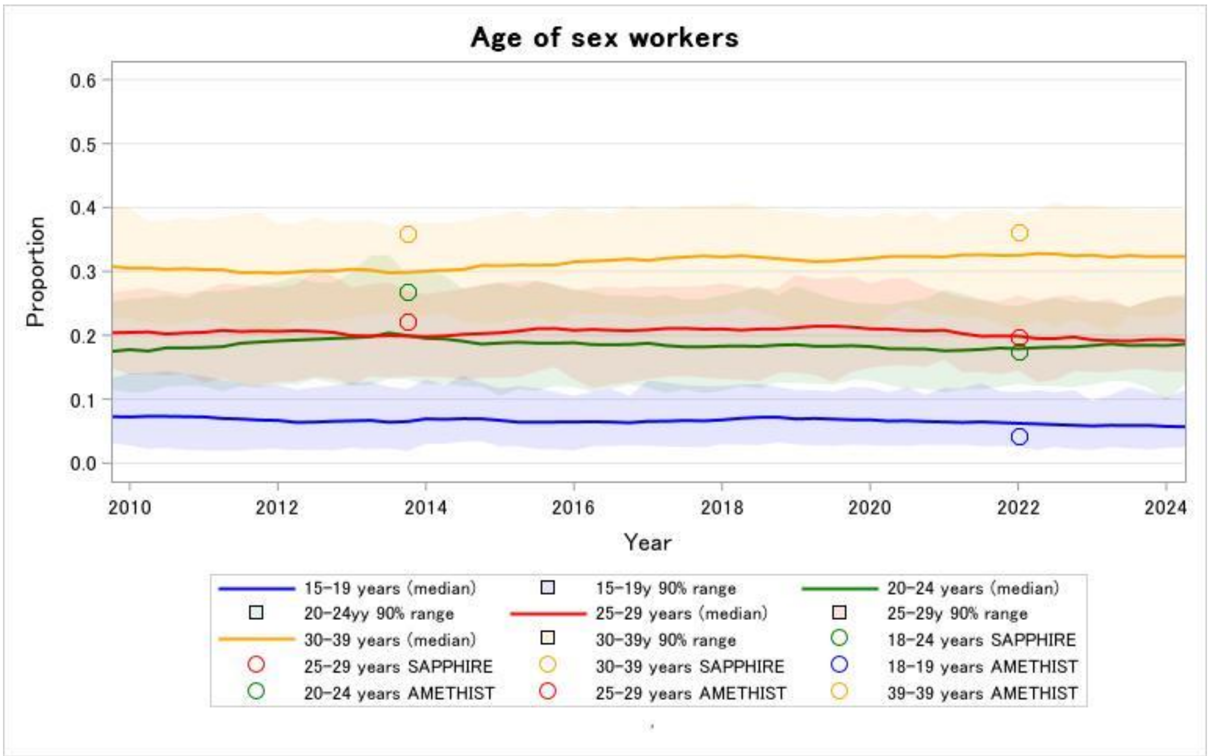

Age of sex workers
Proportion
0.6
0.5
0.4
0.3
0.2
0.1
0.0
2010
2012
2014
2016
2018
2020
2022
2024
Year
15-19 years (median)
15-19y 90% range
20-24 years (median)
20-24yy 90% range
25-29 years (median)
25-29y 90% range
30-39 years (median)
30-39y 90% range
18-24 years SAPPHIRE
25-29 years SAPPHIRE
30-39 years SAPPHIRE
18-19 years AMETHIST
20-24 years AMETHIST
25-29 years AMETHIST
39-39 years AMETHIST

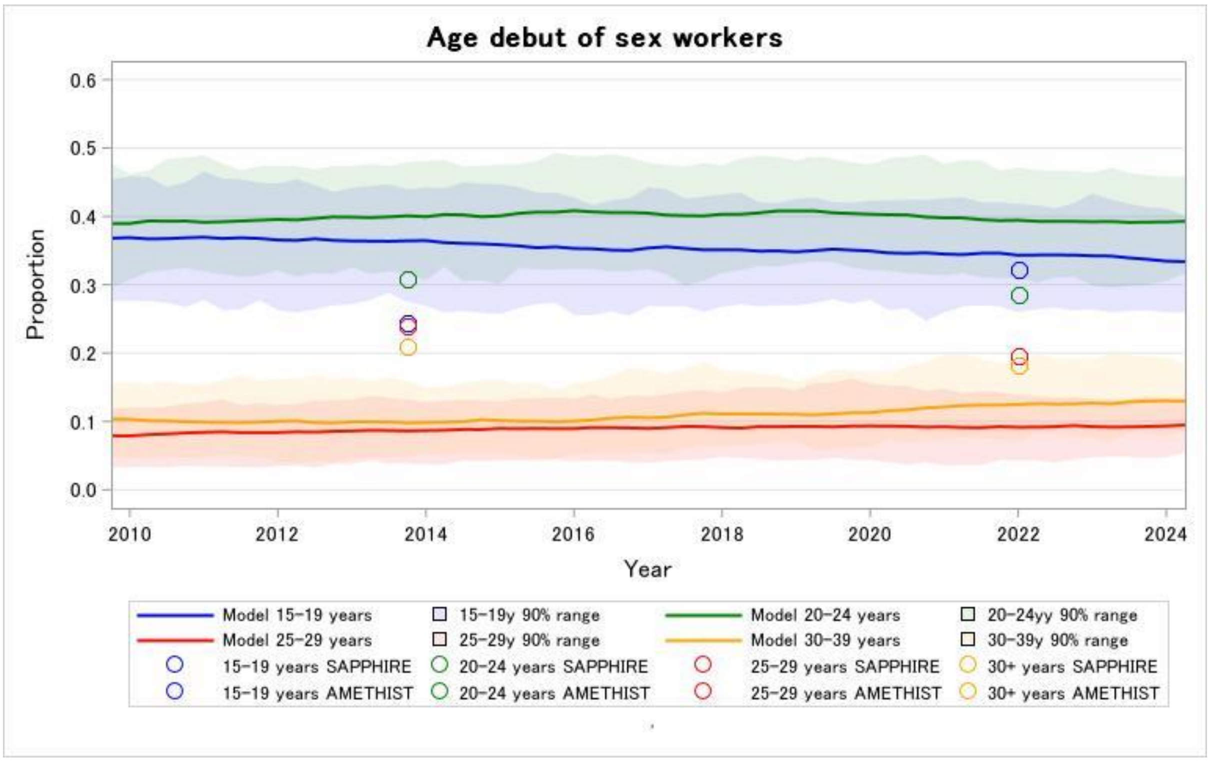
Age debut of sex workers
Proportion
Year
0.0
0.1
0.2
0.3
0.4
0.5
0.6
2010
2012
2014
2016
2018
2020
2022
2024
Model 15-19 years
15-19y 90% range
Model 20-24 years
20-24yy 90% range
Model 25-29 years
25-29y 90% range
Model 30-39 years
30-39y 90% range
15-19 years SAPPHIRE
20-24 years SAPPHIRE
25-29 years SAPPHIRE
30+ years SAPPHIRE
15-19 years AMETHIST
20-24 years AMETHIST
25-29 years AMETHIST
30+ years AMETHIST

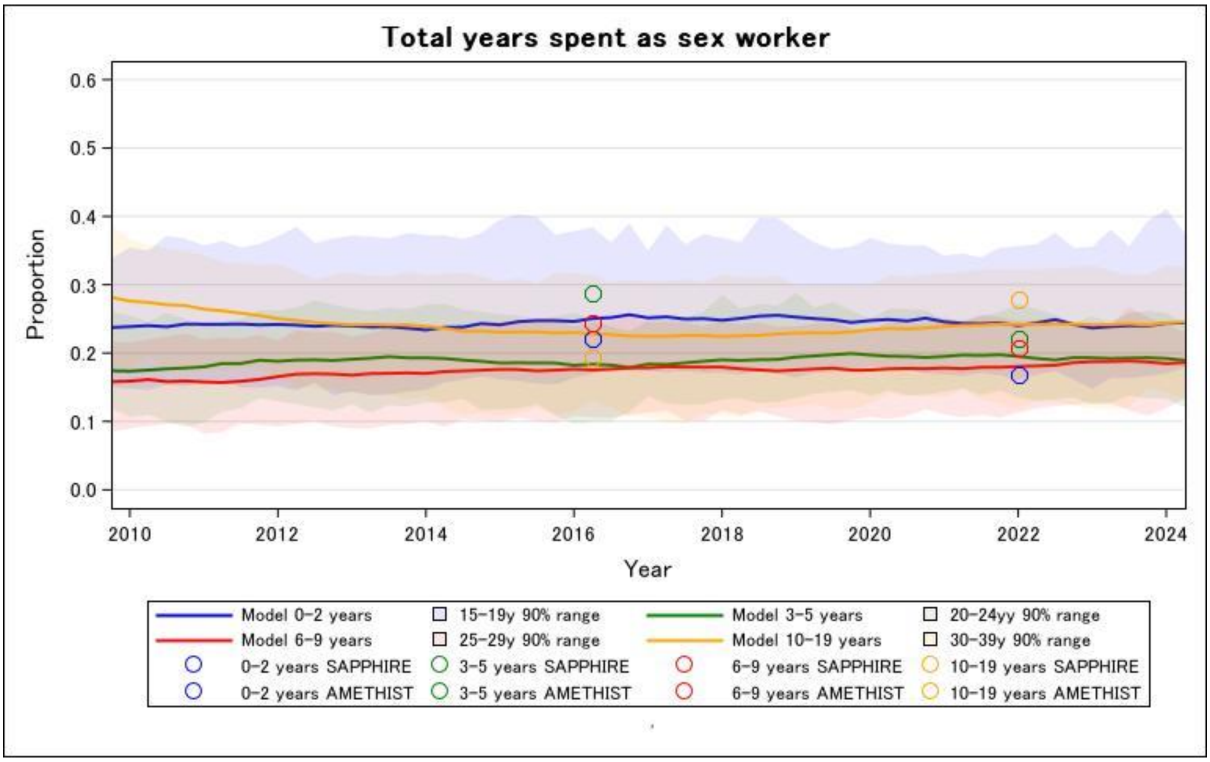
Total years spent as sex worker
Proportion
Year
0.0
0.1
0.2
0.3
0.4
0.5
0.6
2010
2012
2014
2016
2018
2020
2022
2024
Model 0-2 years
15-19y 90% range
Model 3-5 years
20-24yy 90% range
Model 6-9 years
25-29y 90% range
Model 10-19 years
30-39y 90% range
0-2 years SAPPHIRE
3-5 years SAPPHIRE
6-9 years SAPPHIRE
10-19 years SAPPHIRE
0-2 years AMETHIST
3-5 years AMETHIST
6-9 years AMETHIST
10-19 years AMETHIST

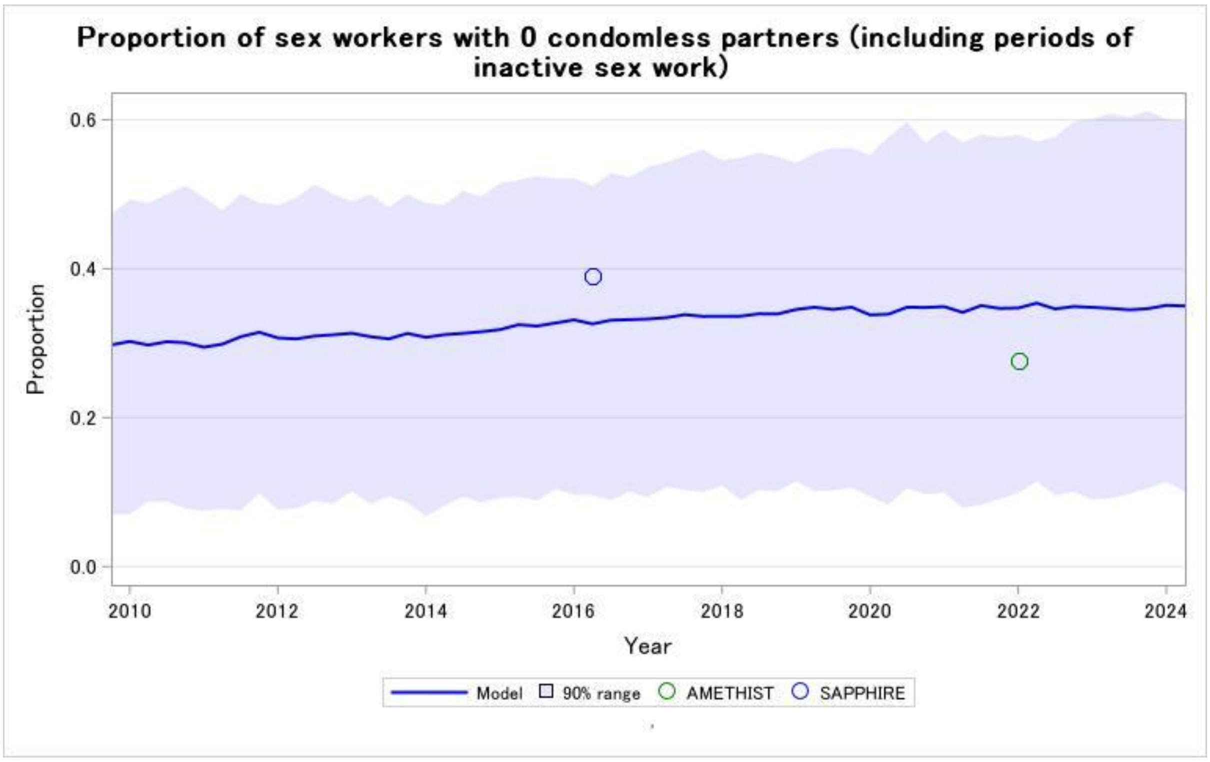

Proportion of sex workers with 0 condomless partners (including periods of inactive sex work)
Proportion
0.0
0.2
0.4
0.6
2010
2012
2014
2016
2018
2020
2022
2024
Year
Model
90% range
AMETHIST
SAPPHIRE

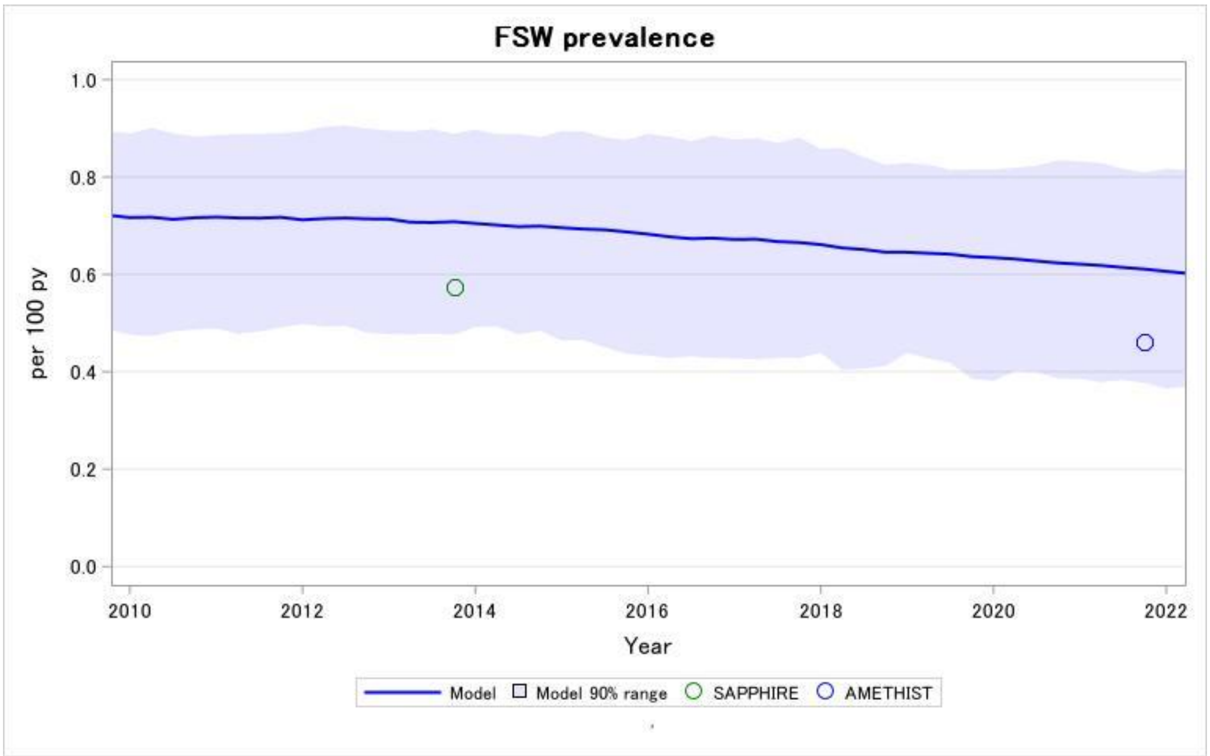

FSW prevalence
per 100 py
0.0
0.2
0.4
0.6
0.8
1.0
2010
2012
2014
2016
2018
2020
2022
Year
Model
Model 90% range
SAPPHIRE
AMETHIST

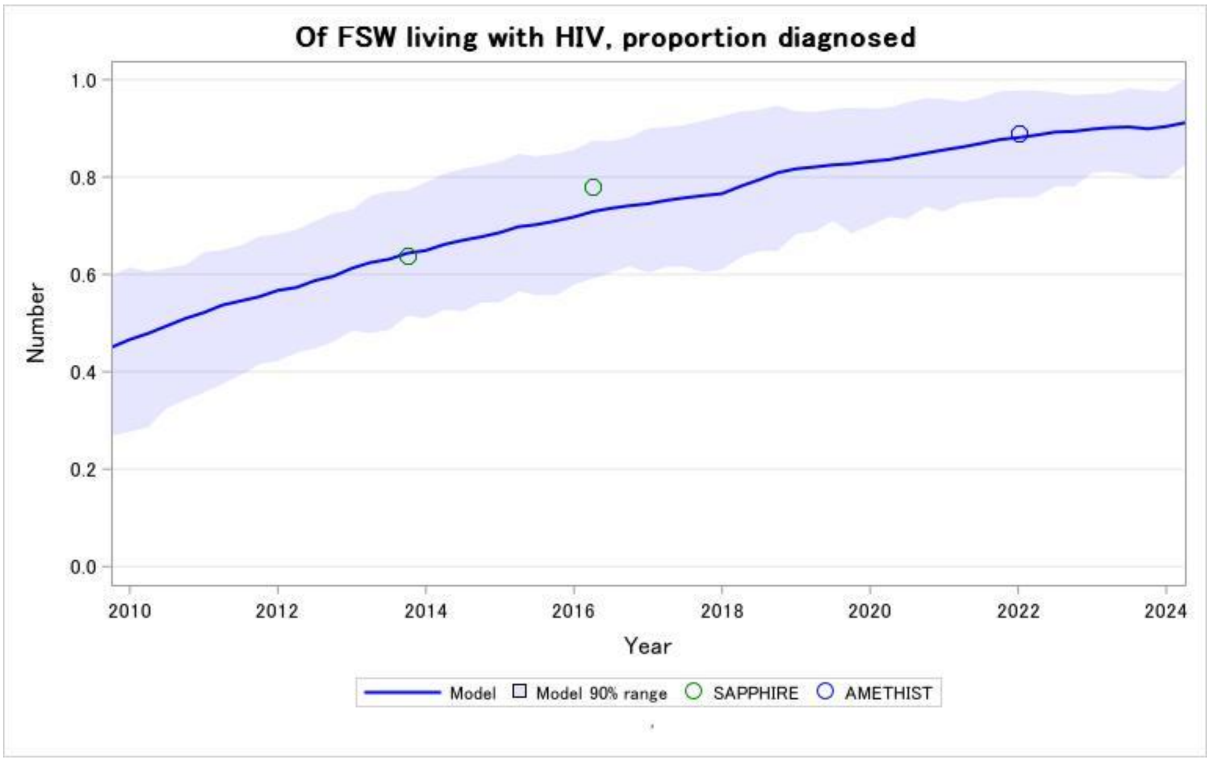
Of FSW living with HIV, proportion diagnosed
Number
Year
Model
Model 90% range
SAPPHIRE
AMETHIST

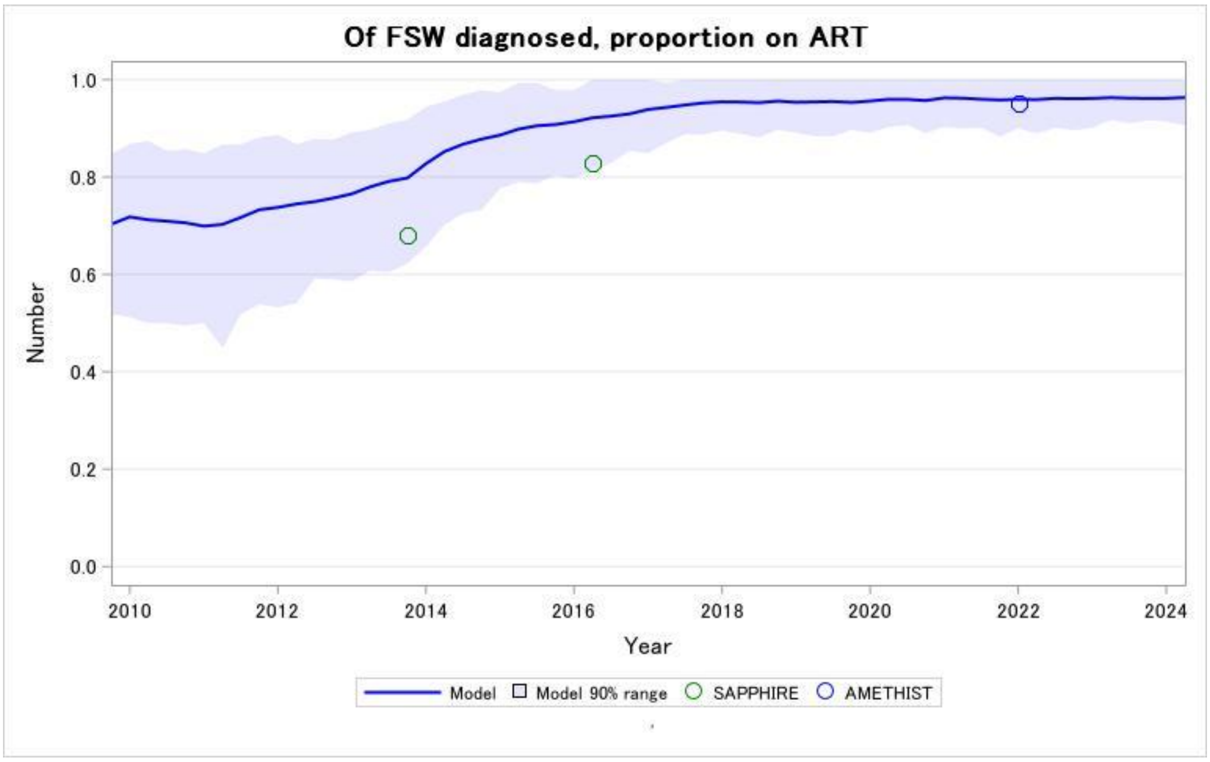
Of FSW diagnosed, proportion on ART
Number
Year
Model
Model 90% range
SAPPHIRE
AMETHIST

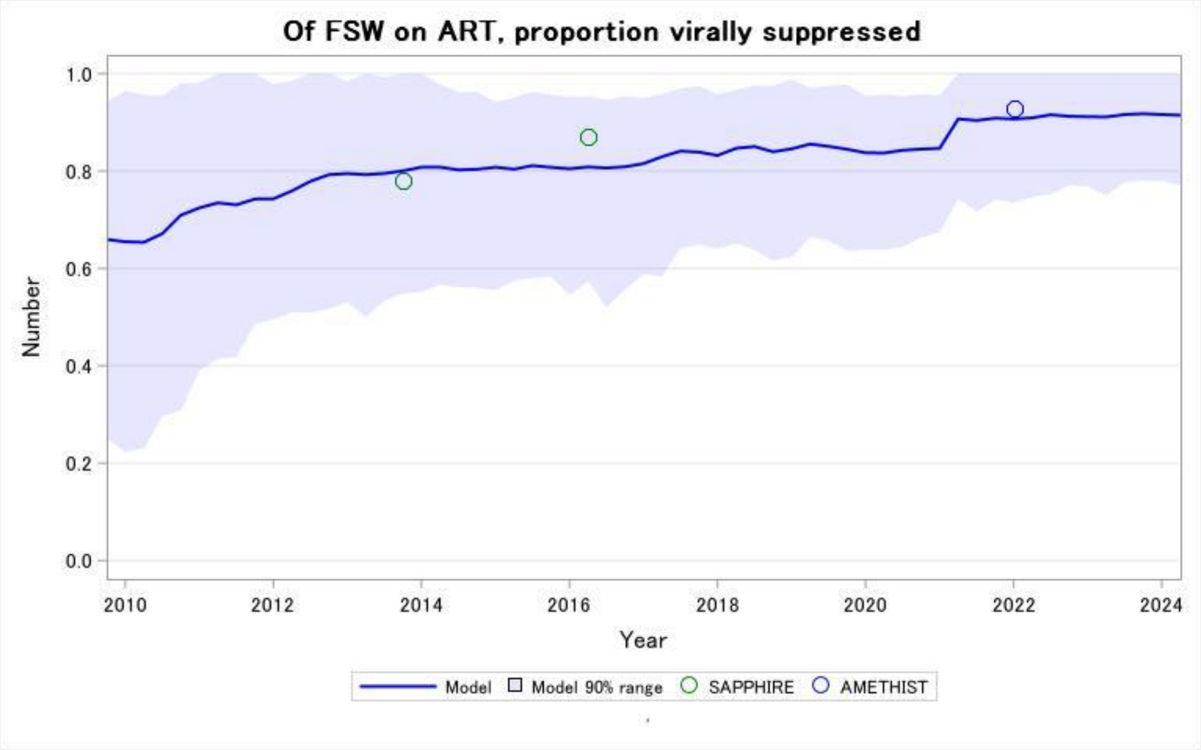

References
'SAPPHIRE' : Cowan FM, Davey CB, Fearon E, Mushati P, Dirawo J, Cambiano V, et al. The HIV Care Cascade Among Female Sex Workers in Zimbabwe: Results of a Population-Based Survey From the Sisters Antiretroviral Therapy Programme for Prevention of HIV, an Integrated Response (SAPPH-IRe) Trial. J Acquir Immune Defic Syndr. 2017;74(4):375-82.

'AMETHIST': Cowan FM, Machingura F, Ali MS, Chabata ST, Takaruza A, Dirawo J, et al. A risk-differentiated, community-led intervention to strengthen uptake and engagement with HIV prevention and care cascades among female sex workers in Zimbabwe (AMETHIST): a cluster randomised trial. Lancet Glob Health. 2024;12(9):e1424-e35.

Fearon et al: Elizabeth Fearon, Sungai T Chabata, Sitholubuhle Magutshwa, Tendayi Ndori-Mharadze, Sithembile Musemburi, Henry Chidawanyika et al. Estimating the Population Size of Female Sex Workers in Zimbabwe: Comparison of Estimates Obtained Using Different Methods in Twenty Sites and Development of a National-Level Estimate. J Acquir Immune Defic Syndr. 2020 Sep 1;85(1):30-38
UNAIDS: Accessed Aug 2025: HIV_estimates_from_1990-to-present.xlsx

Model details

In addition to the details provided below, we encourage anyone wishing to run the model to visit: **HIV Synthesis | HIV Modelling**, and in particular, to view the slideset titled '**Help with program'**: **explanation of hiv_synthesis model program.pptx**

Contents

1 Introduction to the approach taken 15

2 Demographic model 15

2.1 Determination of age in 1989 15

2.2 Pregnancy and parity 18

3 Sexual behaviour and risk of HIV acquisition 20

3.1 Determination of number of short term (condomless sex) partners at period t 21

3.2 Determination of having a long term (condomless sex) partner at period t 32

3.2.1 Starting a new long term condomless partnership at period t 32

3.2.2 Stopping a long term condomless partnership at period t 33

3.3 Determination of number of short term (condomless sex) partners who are HIV infected at time t 34

3.4 Determination of probability that a long term partner is HIV infected at time t 36

3.5 Determination of the risk of infection from a short term partner to the subject 37

3.6 Determination of the risk of infection from a long term partner to the subject 38

3.7 Determination of the risk of infection from the subject to a long term partner 39

3.8 Occurrence of an STI 40

4 Transmitted resistance 40

4.1 Transmitted resistance: details 41

4.2 Loss from majority virus of transmitted mutations 42

5 People being hard to reach for services 42

6 HIV testing and diagnosis of HIV infection 43

7 PrEP 45

7.1 Overview of modelling of (oral) PrEP 45

7.2 Long-acting injectable PrEP 47

8 Male Circumcision 45

9 HIV progression in absence of treatment 53

9.1 Determination of changes in viral load and CD4 count 54

10 Modelling the effect of ART 55

10.1 Modelling the effect of ART - Structure 56
10.2 Initiation of ART 58
10.3 Choice of ART regimen 59
10.4 Monitoring of people on ART 59
10.5 Switch to second line after failure of first line ART 59
10.6 Adherence pattern 60
10.7 Effect of current drug toxicity and current TB or WHO stage 4 condition on adherence 63
10.8 Effect of age and gender on adherence 64
10.9 Effective adherence 69
10.10 Effect of viral load measurement above 1000 cps/mL on adherence 69
10.11 ART interruption / discontinuation 70
10.12 Interruption of ART without clinic being aware 71
10.13 Re-initiation of ART after interrupting in patients still under clinic follow-up 71
10.14 Interruption due to drug stock-outs 72
10.15 Loss to follow-up while off ART (for reasons apart from drug stock-outs) 72
10.16 Effect of ART on viral load, CD4 count, resistance development and drug toxicity 73
10.17 Number of active drugs 73
10.18 Classification of adherence levels 74
10.19 Determination of viral load, CD4 count and risk of resistance in people on ART 74
10.20 Variable patient-specific tendency for CD4 count rise on ART 77
10.21 Accelerated rate of CD4 count loss if PI not present in regimen 77
10.22 Variability in individual (underlying) CD4 counts for people on ART 78
10.23 Viral load and CD4 count changes during ART interruption 82
10.24 Incidence of new current toxicity and continuation of existing toxicity 82
10.25 Switching of drugs due to toxicity 83
10.26 Emergence of specific resistance mutations and their effect on drug activity 84
10.27 New resistance to NNRTI arising as a result of ART interruption 85
10.28 Loss of acquired mutations from majority virus 85
10.29 Determination of level of resistance to each drug 85
10.30 Calculation of activity level of each drug 87
Occurrence of clinical disease and death in HIV infected people 87
11 Local community PEP/PrEP/TLD Access 92
12 Disadvantages in accessibility to care for sex workers and effect of a sex worker program 94
13 Distributions for parameters **Error! Bookmark not defined.**
14 Disability weights and costs 116

References 119

Introduction to the approach taken

The model is an individual-based stochastic simulation model including sexual behaviour, HIV transmission, HIV progression and effects of ART within a sub-Saharan African context (Phillips 2011, Cambiano 2013, Cambiano 2014). Being “individual-based” means that each time the model is run it generates a data set of the simulated lifetime adult experience of a population of people thought to reflect a setting (e.g. a country, district, town, or other small area). The model runs from 1989 (assumed to be the start of the epidemic) with variables updated in 3 month periods. Each run of the simulation program creates 100,000 simulated people who will be age 15 or above at some point between 1989 and 2072. For each simulated person in the data set, there are multiple variables created, such as age, condomless sex partners, male circumcision, oral PrEP use, HIV testing, and, if infected with HIV, HIV diagnosis, CD4 count, viral load, use of specific ART drugs, adherence, resistance. The values of variables are updated every 3 month period for each individual. Below we describe full details.

We apply the model by considering a series of "setting scenarios", each generated by one run of the model by sampling several parameter values to reflect the range of settings in SSA and also to incorporate uncertainty in assumptions. The sampled parameters, which are described in full below in Table S27, include male circumcision rates; initiation of sex work; HIV testing; linkage and retention; ART adherence; resistance emergence, transmission and persistence; ART interruption; extent of implementation of viral load monitoring; rate of switching to 2nd line after detected virologic failure. For each model run we scale up the outputs to a total adult population size in the current base year (i.e. usually the year we are living in at the time or the following year) of 10,000,000. The model is programmed in SAS.

Throughout the sections below we introduce parameters which are indicated in italics. For those parameters for which a value is sampled the distribution is indicated at the end of this document.

Demographic model

Determination of age in 1989

The initial age distribution for both males and females is sampled for each population simulation from three possible distributions representing three different population demographic structures (Table S1). These are chosen such that in the absence of HIV, and given the death rates, the resulting population pyramids and growth rates represent the range of those seen across the setting scenarios (CIA. The World Factbook). Thus a proportion of simulated people have an age below 15 in 1989 (and most are yet to be born). The only variable that is modelled and updated up to reaching the age of 15 (when becoming potentially sexually active) is age itself. The “youngest” person in 1989

is age -68 (i.e. will be born in 2057 and reach age 15 in 2072, just before the modelled period ends.

Table S1. Distribution of ages of simulated individuals in 1989

	Probability of being in age group in 1989		
Age group	Population demographic structure 1	Population demographic structure 2	Population demographic structure 3
-68 to -56	0.180	0.150	0.128
-55 to -46	0.165	0.130	0.119
-45 to -36	0.144	0.120	0.113
-35 to -26	0.114	0.110	0.104
-25 to -16	0.090	0.100	0.097
-15 to -6	0.080	0.090	0.090
-5 to 4	0.068	0.080	0.081
5 to 14	0.047	0.065	0.074
15 to 24	0.036	0.048	0.060
25 to 34	0.027	0.040	0.050
35 to 44	0.021	0.030	0.038
45 to 54	0.016	0.021	0.026
55 to 64	0.012	0.016	0.020

Age specific death rates for uninfected people are based on death rates in South Africa in 1997 (before the significant impact of HIV-related deaths). South Africa has a death registration system and hence provides a reliable setting from which to source death rates. These are given in Table S2. These death rates are modified slightly as described further below where we separate out deaths from non-HIV TB and cardiovascular disease.

Table S2. Age specific death rates (per year)

Age group	Annual death rate	Age group	Annual death rate
Males		Females	
15 – 19	0.0020	15 – 19	0.0015
20 – 24	0.0032	20 – 24	0.0028
25 – 29	0.0058	25 – 29	0.0040
30 – 34	0.0075	30 – 34	0.0040
35 – 39	0.0080	35 – 39	0.0042
40 – 44	0.0100	40 – 44	0.0055
45 – 49	0.0120	45 – 49	0.0075
50 – 54	0.0190	50 – 54	0.0110
55 – 59	0.0250	55 – 59	0.0150
60 – 64	0.0350	60 – 64	0.0210
65 – 69	0.0450	65 – 69	0.0300
70 – 74	0.0550	70 – 74	0.0380
75 – 79	0.0650	75 – 79	0.0500
80 – 84	0.1000	80 – 84	0.0700
≥85	0.4000	≥85	0.1500

Pregnancy, parity and mother to child transmission

In a given three-month period, a women has a probability of becoming pregnant if she has one or more short- or long-term condomless partners. The baseline probability per condomless partner of a woman being pregnant in each three-month period is fixed throughout the simulation. This can be modified by a number of population-level and individual-level factors, described in Table S3. Pregnancy probability is applied separately for each condomless partner. 95% of women are assumed to ever be able to become pregnant and each woman can have a maximum of 10 children (*can_be_pregnant*=0.95). A woman can become pregnant 6 months after their previous birth and the pregnancy lasts for 9 months. We only model pregnancies ending with live birth.

Table S3. Factors affecting risk of pregnancy

<table>
<tr><th>Factor</th><th>Value</th></tr>
<tr><td>Baseline pregnancy risk per three months, prob_pregnancy_base</td><td>0.06 + U(0,0.05). Based on fertility data from (4)</td></tr>
<tr><td>Modifier for overall population fertility</td><td>Overall population-level fertility rate can be higher or lower than the base assumption, sampled from distribution shown below.
<table>
<tr><td>Probability</td><td>33%</td><td>33%</td><td>33%</td></tr>
<tr><td>Factor applied to prob_pregnancy_base</td><td>1.75</td><td>1.0</td><td>1/1.75</td></tr>
</table></td></tr>
<tr><td>Age</td><td>Pregnancy probability is affected by a woman’s age. Based on fertility data from (4)
<table>
<tr><td>Age (years)</td><td>15-24</td><td>25-34</td><td>35-44</td><td>45-54</td><td>55+</td></tr>
<tr><td>Value of fold_preg</td><td>2</td><td>1.9</td><td>1.0</td><td>0.2</td><td>0</td></tr>
</table></td></tr>
<tr><td>Pregnancy risk from short-term condomless partner</td><td>If a woman had one or more short-term condomless partners, pregnancy risk is reduced (per partner) due to the assumed lower number of sex acts with a short-term compared to long-term partner. The factor fold_tr_newp is applied at population level and is the same reduction that also applies to HIV transmission risk from a short-term condomless partner (see Table S27).
<table>
<tr><td>Probability</td><td>33%</td><td>33%</td><td>33%</td></tr>
<tr><td>Value of fold_tr_newp</td><td>0.3</td><td>0.5</td><td>0.7</td></tr>
</table></td></tr>
<tr><td>Desire for more children</td><td>It is assumed that women aged 25-54 years stop desiring more children at a rate of 0.5% per 3 months (termed rate_want_no_more_children), regardless of parity. These women have an 80% reduction in pregnancy risk at each time step.</td></tr>
</table>

Risk of mother to child transmission of HIV is dependent on the viral load of the mother at birth and depends on the parameter *rate_birth_with_infected_child* (0.3: 5%, 0.4: 25%, 0.5: 60%, 0.6: 10%)

- viral load > 100,000: *rate_birth_with_infected_child**2,

- 10,000 – 100,000: *rate_birth_with_infected_child*,
- 1,000 – 10,000: *rate_birth_with_infected_child* /2,
- < 1,000: *rate_birth_with_infected_child/1000*.

For example, if the value of *rate_birth_with_infected_child is 0.5*

- viral load > 100,000: 100% risk,
- 10,000 – 100,000: 50%,
- 1,000 – 10,000: 25%,
- < 1,000: 0.05%.

In order to take into account the benefit of ART in mothers who started ART 3 months ago rate_birth_with_infected_child is divided by 10, if their adherence is above 80%.

Sexual behaviour and risk of HIV acquisition

Here we describe the approach to modelling sexual behaviour and HIV acquisition. The basic approach is summarized in Figure S1. The distributions for parameter values related to sexual behaviour were chosen to reflect uncertainty and variability between settings. Sexual behaviour is characterized by two variables representing, respectively, the number of short term condomless sex partners and whether the person has a current long term condomless sex partner in the 3 month period. The status of long term partners is tracked over time (i.e. if they are infected, diagnosed, on ART, have viral load suppression). Short term partners are not tracked over time, in that if a person has a short term partner in time period *t* who is infected with HIV, this is independent of the probability that any short term partner in time *t*+1 is infected with HIV.

Figure S1 Summary of modelling of sexual behaviour and HIV acquisition

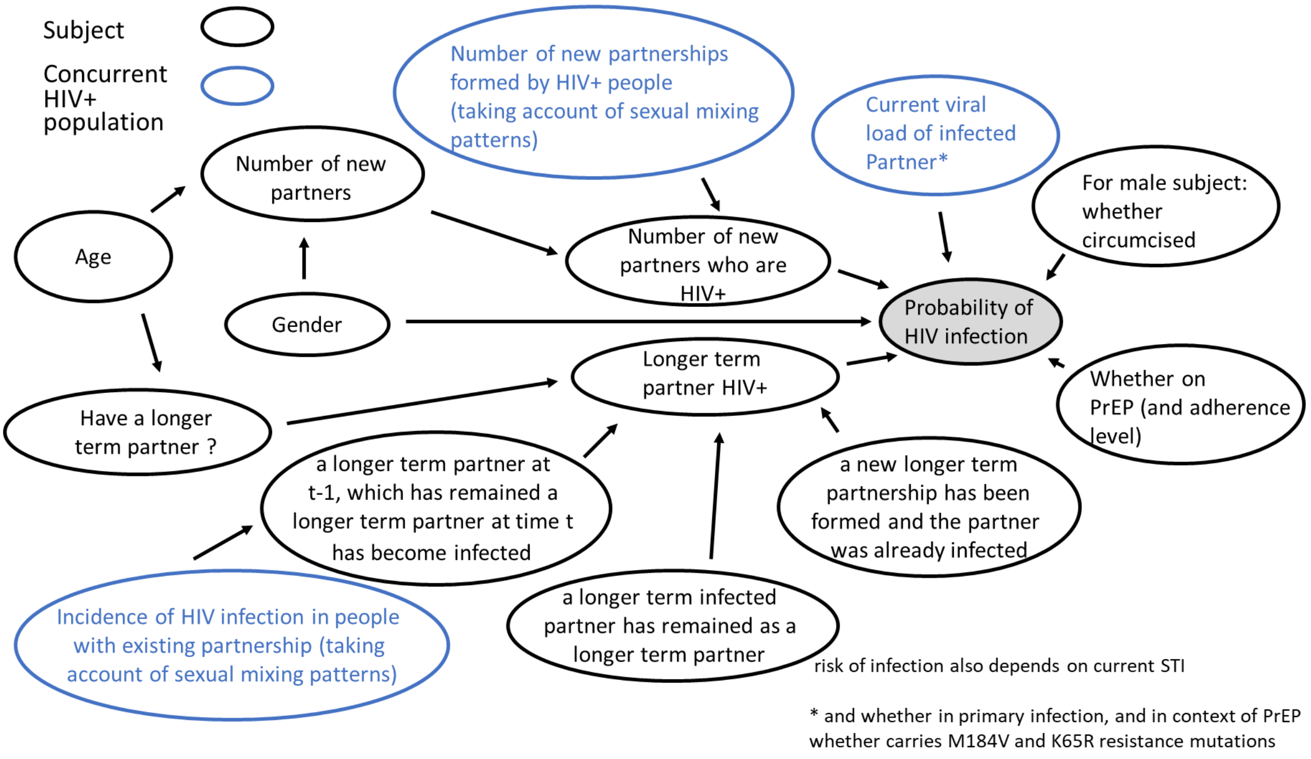

Determination of number of short term (condomless sex) partners at period t

The number of short term partners for an individual in a given period (termed *newp*) is generated at random, according to which sexual behaviour group the person is in for the period. Changes in the sexual behaviour group from time *t*-1 to time *t* are determined by transition probabilities between the groups. These differ for men, women (who are not sex workers) and female sex workers, and can be affected by multiple other factors described below.

For men, there are four sexual behaviour groups: (i) no short term condomless partners in 3 month period, (ii) low number of short term partners (*n*=1-3), (iii) medium number of short term partners (*n*=4-9), and (iv) high number of short term partners (*n*=10-35; Table S4).

For women (who are not sex workers), there are two sexual behaviour groups: (i) no short term condomless partners in 3 month period, (ii) one or more short term condomless partners in 3 month period (*n*=1-9; Table S5). Younger women (age 15-24) can have up to nine short-term condomless partners in a three month period and women aged 25 and older can have up to three.

The lifetime susceptibility of initiating sex work is classed as low, medium or high, conceptually reflecting different social circumstances. Amongst women with medium or high susceptibility for initiating sex work, the probability of initiating sex work in any 3- month period is dependent on four things in addition to the base rate of initiating sex work ((*base_rate_sw*, which is sampled from a distribution of possible values (See Table S27)); whether the susceptibility is medium or high (for high: rr_sw_life_sex_risk_3 = 10) , age (rr_sw_age_1519 = 0.80, rr_sw_age_2024 = 1.00, rr_sw_age_2534= 0.30, rr_sw_age_3549 = 0.03), the overall population levels of sexual risk behaviour and whether the woman has previously been a sex worker (rr_sw_prev_sw = 10). Once a woman has initiated sex work, the probability of stopping sex work is dependent on the base rate of stopping sex work, their age and overall population levels of sexual risk behaviour. These parameter values lead to a distribution of prevalence of sex workers consistent with observed data (Vandepitte 2006, Frascino, Fearon 2020, Cowan 2017, 2019, Lancaster 2016).

For female sex workers, there are five sexual behaviour groups: (i) no short term condomless partners in 3 month period, (ii) low number of short term partners (*n*=1-3), (iii) medium number of short term partners (*n*=4-20), (iv) high number of short term partners (*n*=21-51) and (v) very high number of short term partners (*n*=51-150; Table S6). Sex workers aged more than 30 years were limited to a maximum of 30 short-term condomless partners.

Table S4. Distribution of number of short-term condomless partners within each risk category for men

Risk category							
Zero (*n* = 0)	Number of short-term condomless partners	0					
	Probability	100%					
Low (*n* = 1-3)	Number of short-term condomless partners	1	2	3			
	Probability	50%	30%	25%			
Medium (*n* = 4-9)	Number of short-term condomless partners	4	5	6	7	8	9
	Probability	35%	21%	17%	13%	9%	5%
High (*n* = 10-35)	Number of short-term condomless partners	10	15	20	25	30	35
	Probability	60%	20%	10%	5%	4%	1%

Table S5. Distribution of number of short-term condomless partners within each risk category for women

Risk category										
Zero	Number of short-term condomless partners	0								
	Probability	100%								
Any (*n* = 1-9)	Number of short-term condomless partners	1	2	3	4	5	6	7	8	9
	Age 15-24									
	Probability	30%	20%	15%	12%	9%	6%	4%	2%	2%
	Age 25+									
	Probability	70%	15%	15%	0%	0%	0%	0%	0%	0%

Table S6. Distribution of number of short-term condomless partners within each risk category for female sex workers

Risk category				
Zero (*n* = 0)	Number of short-term condomless partners	0		
	Probability	100%		
Low (*n* = 1-3)	Number of short-term condomless partners	1	2	3
	Probability	70%	15%	15%
Medium (*n* = 4-20)	Number of short-term condomless partners	4-20		
	Probability	Uniform distribution across range		
High (*n* = 21-50)	Number of short-term condomless partners	21-50		
	Probability	Uniform distribution across range		
Very High (*n* = 51-150)	Number of short-term condomless partners	51-150		
	Probability	Uniform distribution across range		

All sex workers aged more than 30 years are limited to a maximum of 30 condomless partners in a three month period.

The transition probabilities p_{gija} of an individual moving from partner group *i* at *t*-1 to partner group *j* at *t* are given by

$$p_{gija} = \frac{f_{gij}}{\left(f_{gi1}+\sum_{j=2}^{k}(f_{gij}r_{ga})\right)} \text{ for j=1}$$

$$p_{gija} = \frac{f_{gij}r_{ga}}{\left(f_{gi1}+\sum_{j=2}^{k}(f_{gij}r_{ga})\right)} \text{ otherwise}$$

(equation 1)

where *g* = 1,2,3 for males, females, and female sex workers, respectively, *a* = 1-10 for age groups 15-, 20-, 25-, 30-, 35-, 40-, 45-, 50-, 55-, 60-, respectively, and *k* is the number of short-term partner categories defined for that population group.

We considered 15 sets of values of f_{gij} each for males and females (who are not sex workers) as shown in Tables S7 and S8, and 3 sets of values for female sex workers (Table S9). These are characterized by substantially different intra-person variability over time in sexual behaviour subgroups. We randomly sampled a matrix independently for each gender for each model run.

Values of r_{ga} are shown in Table S10. These can be modified at time *t* by multiple factors, described in Table S11.

For each individual, actual transitions between groups were determined by random sampling at each time step *t*. The initial allocation of the population across the different risk groups is also sampled from a distribution at the start of each model run.

The number of short-term condomless partners of each individual for that time step, *newp*, is then sampled from the distribution for the appropriate risk group (Tables S4-S6).

For sex workers, the number of short-term condomless partners may then by modified further. Firstly, if there is a reduction in the overall behaviour of the population, leading to a reduced propensity for forming condomless partnerships (*rred_rc*<1), there is a possibility of an additional reduction in sex worker condomless partner numbers.

Probability	33%	33%	33%
Value of *p_rred_sw_newp*	0.01	0.03	0.1

Secondly, if a sex worker is engaged in a low-intensity sex worker program there is a 10% or 5% (*effect_sw_prog_newp* randomly sampled from a uniform distribution) chance that their short-term condomless partners are reduced by two-thirds for that time period. If the sex-worker program is high-intensity, this probability is multiplied by a fold factor of 2 or 3 (*fold_hi_sw_prog_newp* sampled from a Uniform distribution).

Table S7. Values of $\boldsymbol{f_{1ij}}$ (values determining probability of transitioning between short term partner risk behaviour groups) for men.

	Short term partners group in period *t* (*j*)			
Short term partners group in period *t*-1 (*i*)	Zero (*n* = 0)	Low (*n* = 1-3)	Medium (*n* = 4-9)	High (*n* = 10-35)
Sexual behaviour transition matrix 1				
Zero	0.995	0.005	0.005	0.00005
Low	0.95	0.03	0.02	0.00005
Medium	0.03	0.07	0.90	0.00025
High	0	0	0.05	0.95
Sexual behaviour transition matrix 2				
Zero	0.98	0.01	0.01	0.00025
Low	0.98	0.01	0.01	0.00025
Medium	0.05	0.15	0.80	0.00125
High	0	0	0.20	0.80
Sexual behaviour transition matrix 3				
Zero	0.95	0.03	0.02	0.0005
Low	0.93	0.05	0.02	0.0005
Medium	0.20	0.20	0.60	0.0025
High	0	0	0.40	0.60
Sexual behaviour transition matrix 4				
Zero	0.995	0.005	0.005	0.0001
Low	0.95	0.03	0.02	0.0001
Medium	0.03	0.07	0.90	0.0005
High	0.04	0.04	0.09	0.83
Sexual behaviour transition matrix 5				
Zero	0.98	0.01	0.01	0.005

Low	0.98	0.01	0.01	0.0005
Medium	0.05	0.15	0.8	0.0025
High	0.025	0.06	0.17	0.75

Sexual behaviour transition matrix 6				
Zero	0.95	0.03	0.02	0.001
Low	0.93	0.05	0.02	0.001
Medium	0.20	0.20	0.60	0.005
High	0.04	0.08	0.21	0.67

Sexual behaviour transition matrix 7				
Zero	0.995	0.005	0.005	0.000025
Low	0.95	0.03	0.02	0.000025
Medium	0.03	0.07	0.90	0.000125
High	0	0	0.05	0.95

Sexual behaviour transition matrix 8				
Zero	0.98	0.01	0.01	0.000125
Low	0.98	0.01	0.01	0.000125
Medium	0.05	0.15	0.80	0.000625
High	0	0	0.20	0.80

Sexual behaviour transition matrix 9				
Zero	0.95	0.03	0.02	0.00025
Low	0.93	0.05	0.02	0.00025
Medium	0.20	0.20	0.60	0.00125
High	0	0	0.40	0.60

Sexual behaviour transition matrix 10				
Zero	0.90	0.06	0.04	0.0005
Low	0.99	0.005	0.005	0.0005
Medium	0.20	0.20	0.60	0.0025
High	0	0	0.40	0.60

Sexual behaviour transition matrix 11				
Zero	0.90	0.06	0.04	0.001
Low	0.99	0.005	0.005	0.001
Medium	0.20	0.20	0.60	0.005
High	0.04	0.08	0.21	0.67

Sexual behaviour transition matrix 12				
Zero	0.90	0.06	0.04	0.00025
Low	0.99	0.005	0.005	0.00025
Medium	0.20	0.20	0.60	0.00125
High	0	0	0	1.00

Sexual behaviour transition matrix 13				

Zero	0.75	0.15	0.10	0.0005
Low	0.99	0.005	0.005	0.0005
Medium	0.90	0.05	0.03	0.02
High	0.90	0.05	0.03	0.02
**Sexual behaviour transition matrix 14**				
Zero	0.75	0.15	0.10	0.001
Low	0.99	0.05	0.02	0.001
Medium	0.95	0.03	0.01	0.01
High	0.95	0.03	0.01	0.01
**Sexual behaviour transition matrix 15**				
Zero	0.75	0.15	0.10	0.00025
Low	0.93	0.05	0.02	0.00025
Medium	0.80	0.10	0.05	0.05
High	0.80	0.10	0.05	0.05

Table S8. Values of f_{2ij} (values determining probability of transitioning between short term partner risk behaviour groups) for women.

	Short term partners group in period *t* (*j*)	
Short term partners group in period *t*-1 (*i*)	Zero (*n* = 0)	Any (*n* = 1-9)
**Sexual behaviour transition matrix 1**		
Zero (*n* = 0)	0.995	0.005
Any (*n* = 1-9)	0.99	0.01
**Sexual behaviour transition matrix 2**		
Zero (*n* = 0)	0.995	0.005
Any (*n* = 1-9)	0.98	0.02
**Sexual behaviour transition matrix 3**		
Zero (*n* = 0)	0.995	0.005
Any (*n* = 1-9)	0.95	0.05
**Sexual behaviour transition matrix 4**		
Zero (*n* = 0)	0.995	0.005
Any (*n* = 1-9)	0.85	0.15
**Sexual behaviour transition matrix 5**		
Zero (*n* = 0)	0.995	0.005
Any (*n* = 1-9)	0.75	0.25

Sexual behaviour transition matrix 6		
Zero (n = 0)	0.99	0.01
Any (n = 1-9)	0.99	0.01
Sexual behaviour transition matrix 7		
Zero (n = 0)	0.99	0.01
Any (n = 1-9)	0.98	0.02
Sexual behaviour transition matrix 8		
Zero (n = 0)	0.99	0.01
Any (n = 1-9)	0.95	0.05
Sexual behaviour transition matrix 9		
Zero (n = 0)	0.99	0.01
Any (n = 1-9)	0.85	0.15
Sexual behaviour transition matrix 10		
Zero	0.99	0.01
Any (n = 1-9)	0.75	0.25
Sexual behaviour transition matrix 11		
Zero (n = 0)	0.98	0.02
Any (n = 1-9)	0.99	0.01
Sexual behaviour transition matrix 12		
Zero (n = 0)	0.98	0.02
Any (n = 1-9)	0.98	0.02
Sexual behaviour transition matrix 13		
Zero (n = 0)	0.98	0.02
Any (n = 1-9)	0.95	0.05
Sexual behaviour transition matrix 14		
Zero (n = 0)	0.98	0.02
Any (n = 1-9)	0.95	0.05
Sexual behaviour transition matrix 15		
Zero (n = 0)	0.98	0.02
Any (n = 1-9)	0.75	0.25

Table S9. Values of $\boldsymbol{f_{3ij}}$ (values determining probability of transitioning between short term partner risk behaviour groups) for female sex workers.

Short term partners group in period t (*j*)

Short term partners group in period *t*-1 (*i*)	Zero (*n* = 0)	Low (*n* = 1-3)	Medium (*n* = 4-20)	High (*n* = 21-50)	Very High (*n* = 51-150)
Sexual behaviour transition matrix 1					
Zero	0.75	0.24	0.01	0.00	0.00
Low	0.22	0.75	0.03	0.00	0.00
Medium	0.01	0.05	0.93	0.05	0.005
High	0.00	0.00	0.03	0.95	0.02
Very High	0.00	0.00	0.00	0.05	0.95
Sexual behaviour transition matrix 2					
Zero	0.90	0.01	0.00	0.00	0.00
Low	0.01	0.98	0.01	0.00	0.00
Medium	0.00	0.01	0.98	0.01	0.00
High	0.00	0.00	0.01	0.98	0.01
Very High	0.00	0.00	0.00	0.01	0.99
Sexual behaviour transition matrix 3					
Zero	0.20	0.80	0.00	0.00	0.00
Low	0.09	0.90	0.01	0.00	0.00
Medium	0.00	0.01	0.98	0.01	0.00
High	0.00	0.01	0.01	0.97	0.01
Very High	0.00	0.01	0.00	0.08	0.90

Table S10. Baseline values of $\boldsymbol{r_{ga}}$ (factor determining basic* relative level of sexual risk activity by age and gender)

Age group (a=1,10)	Pattern 1 Probability = 15%		Pattern 2 Probability = 15%		Pattern 3 Probability = 30%		Pattern 4 Probability = 30%	
	Males (g=1)	Females (g=2)	Males (g=1)	Females (g=2)	Males (g=1)	Females (g=2)	Males (g=1)	Females (g=2)
15-	0.30	1.80	0.65	1.10	0.05	2.50	0.05	3.00
20-	0.40	1.80	0.65	1.10	0.20	2.50	0.30	3.00
25-	0.85	1.00	1.20	1.00	1.00	1.00	0.80	1.00
30-	1.00	0.80	1.20	0.85	1.00	0.85	0.70	0.85
35-	0.85	0.50	0.65	0.55	0.65	0.55	0.65	0.40
40-	0.50	0.35	0.50	0.45	0.50	0.45	0.50	0.30
45-	0.40	0.30	0.45	0.35	0.45	0.35	0.45	0.15
50-	0.35	0.10	0.40	0.25	0.35	0.03	0.35	0.03
55-	0.20	0.03	0.35	0.20	0.25	0.01	0.25	0.01
60-	0.15	0.02	0.30	0.20	0.15	0.01	0.15	0.01

* Before factors defined below in Table S11 are considered

Table S11. Factors modifying transition probabilities between categories of short-term condomless partners. See also Table S27 below.

<table>
<tr><th>Factor</th><th>Description</th><th>Value</th></tr>
<tr><td>newp_factor</td><td>Underlying propensity of whole population to form short-term partnership with condomless sex.</td><td>Sampled at start of simulation from distribution:<table><tr><td>Probability</td><td>33%</td><td>33%</td><td>33%</td></tr><tr><td>Value of newp_factor</td><td>0.5</td><td>1</td><td>2</td></tr></table></td></tr>
<tr><td>rred_a</td><td>Age-related factor describing the relative propensity of each five-year age group to form short-term condomless partnerships. These are selected at the start of the model run and can be modified at each time step to balance the number of short-term partnerships with condomless sex between different age groups within the population.</td><td>The initial age-specific factors are sampled from the distribution shown in Table S10.
The balancing modifier is calculated every time step within model. Equals 1 if partnerships balance across the sexes, <1 if the number of age-specific partnerships formed by one sex outweighs that reported by the other and vice versa.</td></tr>
<tr><td>rred_p</td><td>Person-specific factor reflecting a person's propensity to be in a higher or lower risk category for short-term condomless partners</td><td>The population-level propensity for fewer short-term condomless partners (termed p_rred_p) is sampled at the start of the simulation from the distribution shown below. Individual-level values are then sampled with p_rred_p defining the probability that rred_p=0.00001; otherwise rred_p=1.<table><tr><td>Probability</td><td>33%</td><td>33%</td><td>33%</td></tr><tr><td>Value of p_rred_p</td><td>0.3</td><td>0.5</td><td>0.7</td></tr></table></td></tr>
<tr><td>rred_adc</td><td>Factor reducing the chance of transitioning to higher risk category for short-term partnerships with condomless sex for people with a AIDS-defining condition</td><td>0.2</td></tr>
<tr><td>rred_adhav</td><td>Whether there is a tendency for people with lower ART adherence to be people who tend to also have higher numbers of short-term condomless partners.</td><td>In 20% of runs, people with an adherence score of less than 0.8 will have rred_adhav=2.0.</td></tr>
<tr><td>rred_d</td><td>Possible reduction in condomless sex following a positive HIV test</td><td>Takes value ch_risk_diag_newp within six months of diagnosis and the square root of ch_risk_diag_newp thereafter. ch_risk_diag_newp is sampled at the start of the simulation for the whole population from the distribution shown below. Informed by Fonner et al (Fonner 2012)</td></tr>
</table>

<table>
<tr><td></td><td></td><td><table><tr><td>Probability</td><td>25%</td><td>25%</td><td>25%</td><td>25%</td></tr><tr><td>ch_risk_diag_newp</td><td>0.7</td><td>0.8</td><td>0.9</td><td>1.0</td></tr></table></td></tr>
<tr><td>rred_balance</td><td>Factor to balance the absolute number of short-term condomless partners between men and women across the population.</td><td>Calculated every time step within model. Equals 1 if total partnerships balance across the sexes, <1 if the number of partnerships formed by one sex outweighs that reported by the other and vice versa.</td></tr>
<tr><td>rred_rc</td><td>Factor representing population-level behaviour change through time with respect to the number of condomless sex partners. In addition, for sex workers, the existence and effectiveness of a sex worker program can affect sexual risk behaviour.</td><td>From 1995-2000, there is a general reduction in condomless sex among the whole population, determined by ych_risk_beh_newp according to the distribution, informed by [Desmond 2018, Glynn 2011]:<table><tr><td>Probability</td><td>20%</td><td>60%</td><td>20%</td></tr><tr><td>Value of ych_risk_beh_newp</td><td>0.6</td><td>0.7</td><td>0.8</td></tr></table>
From 2010-2015, there is the possibility of a further behavioural change, determined by ych2_risk_beh_newp according to the distribution:<table><tr><td>Probability</td><td>5%</td><td>5%</td><td>80%</td><td>5%</td><td>5%</td></tr><tr><td>Value of ych2_risk_beh_newp</td><td>0.95</td><td>0.99</td><td>1</td><td>1 / 0.99</td><td>1 / 0.95</td></tr></table>
For sex workers, the impact of any sex worker program (starting in 2010 in 20% of all model runs) can also modify risk. Sex workers who are engaged with the program (defined by sw_program_visit) have their population-level value for rred_rc further modified by the factor effect_sw_prog_newp.</td></tr>
<tr><td>rred_ep</td><td>Population-level factor modifying chance of transitioning to higher risk category for short-term condomless partnership for those in a long-term partnership.</td><td>Takes value conc_ep, sampled at the start of the simulation for the whole population from the distribution shown below.<table><tr><td>Probability</td><td>33%</td><td>33%</td><td>33%</td></tr><tr><td>Value of conc_ep</td><td>0.33</td><td>1.0</td><td>3.0</td></tr></table></td></tr>
</table>

Determination of having a long term (condomless sex) partner at period t

Note that only condomless sex partnerships are modelled. Thus if a person has a long term partner but condoms are used on all occasions of sexual intercourse then this is not counted as having a long term condomless sex partner, and rates of starting and stopping condomless partnerships can represent changing condom use within an existing long-term partnership as well as the initiation or termination of a new partnership itself.

Starting a new long term condomless partnership at period t

At each period, people with no current long term partner have an age-dependent probability of forming a new long term partnership, termed *eprate* (equation 2).

$$eprate = \frac{0.1e^{0.25*N(0,1)}}{a}$$

(equation 2)

Values of *a*, the age modifier for *eprate*

Age group	15-34	35-44	45-54	55-64
a	1	2	3	5

The probability of starting a new long-term condomless partnership at time *t* is modified by several other factors, described in above.

Table S12. Factors modifying the probability of starting a new long-term condomless partnership.

<table>
<tr><th>Factor</th><th>Description</th><th>Value</th></tr>
<tr><td>ch_risk_beh_ep</td><td>Factor representing possibility of population-level behaviour change through time with respect to condom use in long-term partnerships.</td><td>Calculation of ch_risk_beh_ep over time:
<table>
<tr><td>Time period</td><td>≤1995</td><td>1995-2000</td><td>>2000</td></tr>
<tr><td>Value of ch_risk_beh_ep</td><td>1</td><td>ych_risk_beh_ep ^ (t-1995)</td><td>ych_risk_beh_ep ^ (2000-1995)</td></tr>
</table>
Distribution of ych_risk_beh_ep:
<table>
<tr><td>Probability</td><td>25%</td><td>25%</td><td>25%</td><td>25%</td></tr>
<tr><td>Value of ych_risk_beh_ep</td><td>0.80</td><td>0.90</td><td>0.95</td><td>1.00</td></tr>
</table></td></tr>
<tr><td>ch_risk_diag</td><td>Possible adjustment in condomless sex in long-term partnerships following a positive HIV test.</td><td>Distribution of ch_risk_diag:
<table>
<tr><td>Probability</td><td>25%</td><td>25%</td><td>25%</td><td>25%</td></tr>
<tr><td>Value of ch_risk_diag</td><td>0.7</td><td>0.8</td><td>0.9</td><td>1.0</td></tr>
</table></td></tr>
</table>

<table>
<tr><td>Balancing partnerships</td><td>Adjustment to the probability of forming a new long-term condomless partnership in order to balance the total number reported by men and women within the simulated population, updated each time step.</td><td>Uses the ratio of long-term condomless partnerships reported by men compared to women, termed r_ep_mw, to adjust the probability of starting a new partnership by either sex.

<table>
<tr><td>Value of r_ep_mw</td><td><0.8</td><td>0.8-</td><td>0.9-</td><td>1.1-</td><td>>1.2</td></tr>
<tr><td>Adjustment to probability of partnership formation</td><td>x4 for men</td><td>x2 for men</td><td>No change</td><td>x2 for women</td><td>x4 for women</td></tr>
</table>
</td></tr>
</table>

At the time a long term partnership is started, it is classified into 3 duration groups, each with a different tendency to endure. The percent of people in each group is dependent on age and is shown in Table S13.

Table S13. Percent of newly formed long term partnerships classified into each of three duration groups, each of which has a different tendency to endure (higher class, more durable).

Age group	Partnership duration group		
	1	2	3
15-44	30%	30%	40%
45-54	30%	50%	20%
55-64	30%	70%	0%

Stopping a long term condomless partnership at period t

At time period *t*, for people with a long term partner, the probability of the condomless sex partnership continuing with respect to their partnership duration group is shown in Table S14. It is also modified by *ch_risk_beh_ep*, the parameter conveying the population level change in sexual behaviour with long term partners that occurs from 1995-2000, defined in Table S12. Further, the probability of the partnership ending is reduced by a factor *ch_risk_diag* in the 3 month period after an HIV diagnosis for either the individual or partner.

Table S14. Probability of long-term partnership continuing in time period *t*.

Duration category	1	2	3
Partnership continuation probability	$1 - \frac{0.25}{ch\_risk\_beh\_ep}$	$1 - \frac{0.05}{ch\_risk\_beh\_ep}$	$1 - \frac{0.02}{ch\_risk\_beh\_ep}$

The probability that a partnership continues is also modified by an age- and sex-specific factor that is updated each time step to ensure that the numbers of long-term condomless partners reported by each age group approximately mirror the reciprocal number reported by the opposite sex.

Note also that levels of sexual behaviour, in terms of numbers of short term partners and the probability of a long term partner are essentially determined by the levels of such sexual behaviour required in order to produce an epidemic as described, given rates of transmission with condomless sex partners. Sexual behaviour tends to be under-reported particularly in women and higher levels of behaviour have to be assumed both to be consistent with levels of risk behaviour reported in men, and to generate an epidemic of the proportions observed (e.g. Desmond 2018, Glynn 2011, Yeatman 2011, Gregson 2002, Johnson 2002).

Determination of number of short term (condomless sex) partners who are HIV infected at time t

For each short term partner that a subject has at time *t*, the probability that the partner is infected is calculated. This is dependent on the prevalence of HIV in those of the opposite gender themselves having short term partners, taking consideration of age mixing. If the subject is of gender *g* and age group *a*, then for each short term partner the first step is to determine by sampling at random, the age group of the short term partner, a^{newp} (in fact, for simplicity, all short term partners at time *t* are assumed to be in this same age group). The gender and age mixing probabilities used are sampled independently for each gender in each run from the matrices shown in Table S15.

Table S15. Sexual mixing matrices by age and gender. The proportion of short term partnerships formed by men in age group a_m which are with females of age group a_f and the proportion of short term partnerships formed by females in age group a_f which are with men of age group a_m.

A. Men					
	Female age groups (a_f)				
Male age groups (a_m)	15-24	25-34	35-44	45-54	55-65
Sex age mixing matrix 1					
15-24	0.865	0.11	0.025	0.00	0.00
25-34	0.47	0.43	0.10	0.00	0.00
35-44	0.30	0.50	0.20	0.00	0.00
45-54	0.43	0.30	0.23	0.03	0.01
55-64	0.18	0.18	0.27	0.27	0.10
Sex age mixing matrix 2					
15-24	0.865	0.11	0.025	0.00	0.00
25-34	0.47	0.43	0.10	0.00	0.00
35-44	0.20	0.35	0.40	0.05	0.00
45-54	0.15	0.23	0.25	0.30	0.07
55-64	0.05	0.08	0.25	0.30	0.32
Sex age mixing matrix 3					
15-24	0.90	0.05	0.02	0.02	0.01
25-34	0.44	0.43	0.10	0.02	0.01
35-44	0.20	0.34	0.40	0.05	0.01
45-54	0.15	0.23	0.25	0.30	0.07

55-64	0.05	0.08	0.25	0.30	0.32
Sex age mixing matrix 4					
15-24	0.93	0.05	0.01	0.01	0.00
25-34	0.50	0.40	0.08	0.01	0.01
35-44	0.20	0.34	0.41	0.05	0.00
45-54	0.15	0.20	0.25	0.37	0.03
55-64	0.05	0.08	0.20	0.40	0.27
Sex age mixing matrix 5					
15-24	0.94	0.05	0.01	0.00	0.00
25-34	0.50	0.40	0.08	0.01	0.01
35-44	0.40	0.40	0.15	0.04	0.01
45-54	0.30	0.30	0.25	0.10	0.05
55-64	0.30	0.30	0.30	0.05	0.05
Sex age mixing matrix 6					
15-24	0.94	0.05	0.01	0.00	0.00
25-34	0.50	0.40	0.08	0.02	0.00
35-44	0.50	0.35	0.10	0.05	0.00
45-54	0.50	0.35	0.10	0.05	0.00
55-64	0.50	0.35	0.10	0.05	0.00

B. Women					
	Male age groups (a_m)				
Female age groups (a_f)	15-24	25-34	35-44	45-54	55-65
Sex age mixing matrix 1					
15-24	0.43	0.34	0.12	0.10	0.01
25-34	0.09	0.49	0.30	0.10	0.02
35-44	0.03	0.25	0.34	0.25	0.13
45-54	0.00	0.00	0.05	0.70	0.25
55-64	0.00	0.00	0.00	0.10	0.90
Sex age mixing matrix 2					
15-24	0.43	0.415	0.12	0.03	0.005
25-34	0.09	0.50	0.35	0.05	0.01
35-44	0.03	0.25	0.34	0.25	0.13
45-54	0.00	0.00	0.05	0.70	0.25
55-64	0.00	0.00	0.00	0.10	0.90
Sex age mixing matrix 3					
15-24	0.25	0.55	0.15	0.03	0.02
25-34	0.09	0.50	0.35	0.05	0.01
35-44	0.03	0.25	0.34	0.25	0.13
45-54	0.00	0.00	0.05	0.70	0.25
55-64	0.00	0.00	0.00	0.10	0.90
Sex age mixing matrix 4					

15-24	0.05	0.55	0.35	0.03	0.02
25-34	0.03	0.52	0.40	0.03	0.02
35-44	0.03	0.05	0.57	0.30	0.05
45-54	0.00	0.00	0.05	0.70	0.25
55-64	0.00	0.00	0.00	0.10	0.90

Sex age mixing matrix 5					
15-24	0.05	0.45	0.30	0.15	0.05
25-34	0.01	0.40	0.39	0.15	0.05
35-44	0.01	0.07	0.47	0.30	0.15
45-54	0.00	0.00	0.05	0.70	0.25
55-64	0.00	0.00	0.00	0.10	0.90

Sex age mixing matrix 6					
15-24	0.20	0.20	0.20	0.20	0.20
25-34	0.00	0.25	0.25	0.25	0.25
35-44	0.01	0.01	0.32	0.33	0.33
45-54	0.00	0.00	0.05	0.70	0.25
55-64	0.00	0.00	0.00	0.10	0.90

Then, for the given partner (of gender 1-*g* and age group a^{newp}), the risk that the partner is infected is then given by

$$h_{gat} = \frac{\sum_{a^{\text{newp}},(g-1)} L_{(t-1)}^{\text{inf}}}{\sum_{a^{\text{newp}},(g-1)} L_{(t-1)}}$$

(equation 3)
where $L_{(t-1)}^{\text{inf}}$ is the total number of infected short-term partners at time (*t*-1), and $L_{(t-1)}$ is the total number of short term partners at time *t*-1. The numerator is therefore the total number of infected short term partnerships of the opposite gender in age group a^{newp}..
Since we assume that all short term partners at time *t* are in this same age group, the total number of infected short term partners that the subject has at time *t*, L_t^{inf}, is then given by

$$L_t^{\text{inf}} = \text{Min}(\text{Poisson}(h_t \cdot L_t), L_t)$$

(equation 4)

Determination of probability that a long term partner is HIV infected at time t

E_t^{inf} indicates whether the subject has a long term (condomless sex) partner who is infected ($E_t^{\text{inf}} = 1$ if infected, else $E_t^{\text{inf}} = 0$). A long term partner at time *t* can be infected either because (i) a new long term partnership has been formed and the partner was already infected, (ii) because a long term partner at *t*-1, which has remained a long term partner at time *t*, has become infected, or (iii) because an infected long term partner has remained as a long term partner.
For (i):
It is assumed that 50% of new long-term condomless partners were previously a long-term condomless partner of the individual (for example, if condom use has started and then stopped within an ongoing partnership); for these individuals, if the partner was

previously recorded as being HIV-infected then they remain HIV-infected. For new partners who are not known to be already infected, the probability of infection is based on the HIV prevalence of infection in the previous time step among subjects of age group *a* and gender 1-*g* (equation 5).

$$\begin{cases} E_t^{\text{inf}} = 1, \; U < p_{a(1-g)(t-1)} & \text{where } U \text{ randomly sampled from } Uniform(0,1) \\ E_t^{\text{inf}} = 0, \; \text{otherwise} & \end{cases}$$

(equation 5)

where $p_{a(1-g)(t-1)}$ is the HIV prevalence.

For (ii):

The probability that a long term partner of a subject of age group *a* and gender *g* becomes infected from a different partner is derived from the HIV incidence at *t*-1 for age group *a* (i.e. the same age group) and gender 1-*g*, $i_{a(1-g)(t-1)}$ among the sexually active population who have both a long term partner and at least one short term partner (equation 6).

$$\begin{cases} E_t^{\text{inf}} = 1, & U < i_{a(1-g)(t-1)} \text{ where } U \text{ randomly sampled from } Uniform(0,1) \\ E_t^{\text{inf}} = 0, & \text{otherwise} \end{cases}$$

(equation 6)

In order to maintain balance, for each gender, between the number of uninfected people with a long term partner who is infected, and the number of infected people with a long term partner who is uninfected, this incidence $i_{a(1-g)(t-1)}$ is modified at time *t* dependent on the degree of balance at time *t*-1.

For (iii):

If $E_{(t-1)}^{\text{inf}} = 1$ and $E_t \geq 1$ then assign $E_t^{\text{inf}} = 1$

(equation 7)

Determination of the risk of infection from a short term partner to the subject

For each HIV infected short term partner of a subject of gender *g* and age group *a* the viral load group, *v*, of the partner is obtained by sampling from the viral load distribution of those of the opposite gender. Thus we sample from Uniform(0,1), where the probability of the partner having viral load in group *v* is given by

$$\frac{\sum_v L_{(t-1)}^{\text{inf}}}{\sum L_{(t-1)}^{\text{inf}}}$$

(equation 8)

where the numerator is the total number of short-term partnerships had by infected people in viral load group *v* and the denominator is the total number of short-term partnerships had by infected people (in any viral load group).

Viral load groups are:

(1) < 2.7 log cps/mL
(2) 2.7-3.7 log cps/mL
(3) 3.7-4.7 log cps/mL
(4) 4.7-5.7 log cps/mL
(5) ≥ 5.7 log cps/mL
(6) primary infection.

Once the viral load group, v, of the infected partner is determined, the probability, t_v, of the subject being infected by the partner is then given according to:

(1) t_1 = Normal (tr_rate_undetec_vl**fold_tr_newp*, 0.000025^2)
(2) t_2 = Normal (0.01**fold_tr_newp*fold_tr*, 0.0025^2)
(3) t_3 = Normal (0.03**fold_tr_newp*fold_tr*, 0.0075^2)
(4) t_4 = Normal (0.06**fold_tr_newp*fold_tr*, 0.015^2)
(5) t_5 = Normal (0.10**fold_tr_newp*fold_tr*, 0.025^2)
(6) t_6 = Normal (0.16**fold_tr_newp*, 0.075^2)

These are based on Hollingsworth et al (2008) and Bellan (2015), which are estimated for a longer term partner.
The transmission risk from a short term partner is multipled by *fold_tr_newp* due to the assumed lower number of sex acts in short-term partnerships and *fold_tr* for viral load groups 2-5 only to represent underlying variability in transmissibility across the whole population. These probabilities are increased by *fold_change_w*-fold for female subjects aged $\geq$ 20, by *fold_change_yw*-fold for female subjects aged < 20, by *fold_change_sti*-fold if the person has an existing STI (risk of a new STI in any one three month period is given by the number of short term condomless partners / 20, and risk of an STI persisting to each subsequent time step is given by the number of short term condomless partners / 5) (Cohen 1998), and decreased by 60% if a male partner is circumcised (Bailey RC, Auvert 2005, Gray 2012). The risk is decreased by 90% or 95% (determined by random sampling) if the subject is on PrEP with > 80% adherence (details of PrEP use are given below) (Heffron 2018) Full details of the effects of PrEP are described in Section 6. Each of these probabilities is sampled once for the whole simulation from the distributions shown in Table S27 below.

In 20% of model runs, it is assumed that short-term partners can have a lower level of viral load suppression (*exp_setting_lower_p_vl1000*) due to short-term migration; in these runs, for individuals aged 20-49 years, the probability of a short-term condomless partner being virally suppressed <1000 cells/mL is adjusted by 1/(1+uniform(0,1)) (termed '*external_exp_factor*') for 1% of men and 0.05% of women (*rate_exp_set_lower_p_vl1000*).
We assume that super-infection can occur (i.e. a person can be re-infected with HIV with consequent risk of acquiring new mutations).
Realization of whether the subject is infected by each short term partner is determined by sampling from Uniform(0,1).

Determination of the risk of infection from a long term partner to the subject

Infected long term partners at time t are classified by whether they are in primary infection (if infection occurred at t-1), whether they are diagnosed with HIV, whether they are on ART, and whether their current viral load is < 2.7 cps/mL or not.

The probability of a long term partner with HIV being diagnosed at time t, $p_t^{e,diag}$, is determined by the proportion of all HIV-infected long-term condomless partners who are diagnosed at time t-1, $p_{t-1}^{e,diag}$, adjusted according to the difference between this value and the proportion of subjects with HIV who are diagnosed, $\frac{T_{(t-1)}^{diag}}{T_{(t-1)}^{inf}}$ (equation 9).

$$\begin{cases} \text{if } d_{(t-1)}^{e,diag} < 0 & \text{then } p_t^{e,diag} = 0 \\ \text{if } 0 < d_{(t-1)}^{e,diag} \leq 0.05 & \text{then } p_t^{e,diag} = \frac{p_{t-1}^{e,diag}}{5} \\ \text{if } 0.05 < d_{(t-1)}^{e,diag} < 0.10 & \text{then } p_t^{e,diag} = \frac{p_{t-1}^{e,diag}}{2} \\ \text{if } 0.10 < d_{(t-1)}^{e,diag} & \text{then } p_t^{e,diag} = p_{t-1}^{e,diag} \end{cases}$$

$$\text{where } d_{(t-1)}^{e,diag} = \frac{T_{(t-1)}^{diag}}{T_{(t-1)}^{inf}} - p_{(t-1)}^{e,diag}$$

(equation 9)

$T_{(t-1)}^{diag}$ is the total number of subjects diagnosed with HIV at time t-1 and $T_{(t-1)}^{inf}$ is the total number of subjects with HIV (diagnosed and undiagnosed) at time t-1.

The proportion of those diagnosed who are on ART, and the proportion of those on ART who have viral load < 2.7 log cps/mL are determined in a similar manner. In this way the proportions diagnosed with HIV, on ART, and with current viral load is < 2.7 log cps/mL are kept similar for the long term partners as in the simulated subjects themselves.

Risk of infection from a long term infected partner is determined by Normal (0.001, 0.075^2) if the existing partner is in primary infection (ie. infected at t-1), Normal (0.16, 0.000025^2) if the existing partner has viral load < 2.7 log cps/mL, and Normal (0.05**fold_tr*, 0.0125^2) otherwise.

Similar to short-term partners, the transmission risk from a long-term partner is multipled by *fold_tr* for partners who are not in primary infection or virally suppressed to represent underlying variability in transmissibility across the whole population; by *fold_change_w*-fold for female subjects aged $\geq$ 20 and by *fold_change_yw*-fold for female subjects aged < 20; by *fold_change_sti*-fold if the person has an existing STI; and is decreased, exactly as for short term partners above, if a male partner is circumcised or the subject is on PrEP.

Determination of the risk of infection from the subject to a long term partner

If the subject is infected and a long-term condomless partner is not, there is the possibility of HIV transmission from the subject to the partner. First, the probability that the subject is the long-term partner's only sexual partner is estimated from the proportion of subjects of that age group who only have a long-term partner (compared to those who have a long-term and short-term condomless partners) in that time step. For long-term partners in a monogamous partnership with the subject, the risk of HIV infection is based on the subject's viral load group, modified by *fold_tr*, *fold_change_w*, *fold_change_yw* and *fold_change_sti* (based on the subject rather than the partner's STI status) as described above.

For long-term partners who are assumed to also have other short-term condomless partners (who not explicitly modelled), risk of infection each time step is based on the HIV incidence for all subjects of that gender and age group who have a long-term partner and at least one short-term partners.
Balancing of infection rates from subjects to long-term partners is achieved by comparing the number discordant partnerships from the perspective of infected men with an uninfected female partner compared to uninfected women with an infected male partner (and vice versa) and adjusting the number of transmissions accordingly. A further balancing adjustment is made to equalize the number of concordant positive partnerships reported by men and women by dissolving some partnerships if the ratio differs from one.

Occurrence of an STI

The risk of developing an STI in a given 3 month period is given by *rate_sti* (0.05) times the value of newp. For people with an STI in one period the probability of persistence to the next is given by *rate_persist_sti* (0.20).

Transmitted resistance

The modelling of transmission of drug resistance is summarized in Figure S2. The presence or not of resistance mutations does not influence the risk of transmission (i.e. virus with resistance mutations present is assumed equally transmissible as virus without such mutations, for a given viral load). The probability that resistance mutations present in majority virus of the source partner are transmitted to the newly infected person is dependent on the specific mutation. Once a resistance mutation is transmitted to the new host it is assumed to have a certain probability of being lost from majority virus over time (Castro).   Even after being lost from majority virus, it is assumed to remain in minority virus and is selected back as majority virus if an antiretroviral drug selecting for that mutation is initiated.   We also consider the possibility of a person who is already infected become super-infected, including with drug resistant HIV (Smith 2005), although there is assumed to be at most a 20% chance (80% chance in 10% of model runs, determined by *super_inf_res*) that a person super-infected by a person with HIV resistance then has virus with those resistance mutations as a result.

Figure S2. Overview of modelling of transmission of drug resistance

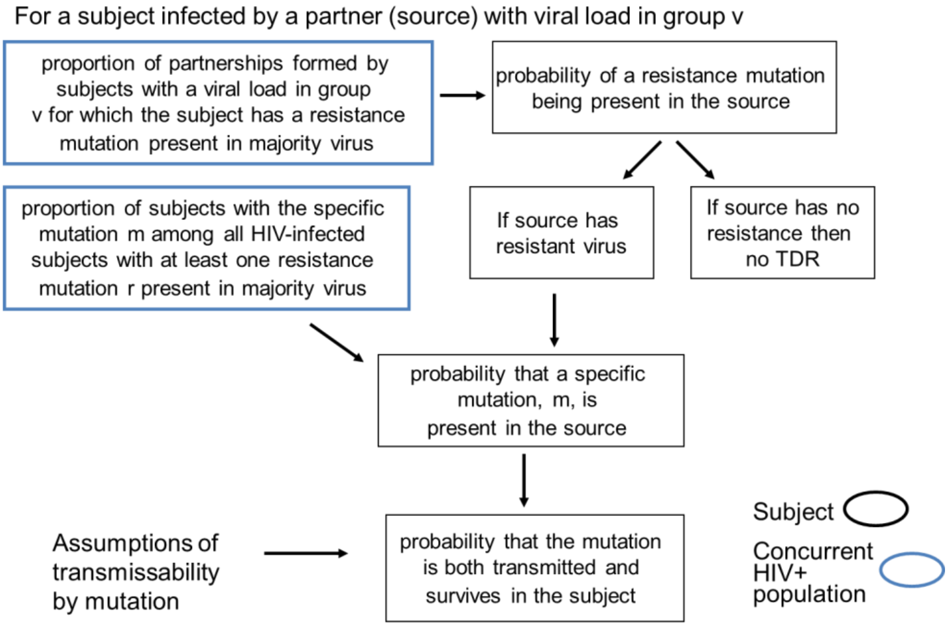

Transmitted resistance: details

The viral load group of the person who infected the subject is known, as indicated above. For a subject infected by a person in viral load group v the probability of a resistance mutation being present in the infected person is given by

$$\frac{\sum_{v,\ \text{and mutation present}} L_{(t-1)}^{\text{inf}}}{\sum_{v} L_{(t-1)}^{\text{inf}}}$$

where $\sum_{v,\ \text{and mutation present}}$ is the sum over all partnerships had by HIV-infected people in viral load group v for whom a resistance mutation is present in majority virus and $\sum_{v}$ is the sum over all HIV-infected subjects in viral load group v. Again, realization of whether the subject is infected by a person with at least one resistance mutation in majority virus is determined by sampling from Uniform(0,1).

For subjects infected from a source partner with a resistance mutation, the probability that a specific mutation, m, is present in the source is given by

$$\frac{\sum_{\text{mutation } m \text{ present}} L_{(t-1)}^{\text{inf}}}{\sum_{\text{mutation present } v} L_{(t-1)}^{\text{inf}}}$$

Where $\sum_{\text{mutation } m \text{ present}}$ is the sum over all HIV-infected subjects with mutation m present in majority virus and $\sum_{\text{mutation present}}$ is the sum over all HIV-infected subjects with at least one resistance mutation in majority virus.

If a given resistance mutation, m, is present in the source partner, the probability that the mutation is both transmitted and survives in the subject (i.e. that its presence will affect future response to drugs for which the mutation confers reduced sensitivity) is mutation specific (Table S16). We consider uncertainty in the extent to which NNRTI and INSTI transmitted resistance mutations are effectively immediately lost (even from minority virus) by sampling from a distribution for parameters *res_trans_factor_nnrti and res_trans_factor_ii*, which are sampled at the start of each run.

Table S16. Table of probabilities that for a given mutation present in the source partner the mutation is both transmitted and survives in the subject (based on evidence from studies comparing distribution of resistance mutations between treated and antiretroviral naïve populations; (e.g. Corvasce 2006, Turner 2004) and modelling of HIV in MSM in the UK (Phillips 2013)

M184V	0.2
K65R	0.2
Q151M	0.5
Thymidine analogue mutations (TAMS)	0.5
NNRTI mutations (K103N, G190A, Y181C)	1 - (0.20**res_trans_factor_nn*)
PI mutations	0.5
Integrase inhibitor mutations (*res_trans_factor_ii*)	sampled from 0.2, 0.4, 0.6 0.8

Loss from majority virus of transmitted mutations

There is a probability per 3 months of loss of persistence of transmitted mutations from majority virus to minority virus (same for each mutation) *rate_loss_persistence,* which is one of the parameters sampled at the start of each model run (see Table S27 below). This is again informed by fitting of a model of HIV in MSM to UK data UK (Phillips 2013)

People being hard to reach for services

A proportion of people have a long term propensity not to take up HIV services (including testing, PrEP, VMMC), for various possible reasons including stigma, physical barriers, etc. (Grimsrud 2020) ("hard to reach"). The proportion of women that have a propensity to be hard to reach with prevention and testing services is given by *p_hard_reach_w* (sampled from Uniform(0.05, 0.20) for each run / setting scenario; see Table S27). The extent to which this is higher in men is given by a parameter *hard_reach_higher_in_men* (sampled from Uniform(0,0.1).
Limited data are available to inform this parameter (proxy variables are the proportion who reported never being tested for HIV and the proportion who refuse HIV testing), nevertheless we considered it important to take this into account, given the evidence that not everyone accepts HIV testing for various reasons. The level of acceptability of provider initiated HIV testing and counselling (PITC) in resource limited settings is extremely variable from levels of 99%, observed in inpatients in Uganda (Wanyenze 2011) to 31% among outpatients in South Africa (Bassett 2007). Among pregnant women the level of acceptability of PITC seems to be higher, varying from 76 to 99.9% (Henson 2012), while the estimated acceptability of home-based counselling and testing has been estimated in a meta-analysis to be 83% (Sabapathy 2012). This variability seems to be related mainly to the quality of the intervention delivered and calendar time.

HIV testing and diagnosis of HIV infection

HIV testing in the general population

HIV testing was assumed introduced in mid-2003 (*date_start_testing*) for testing of symptomatic people and in antenatal clinics and in 2009 for the general population. People who are hard to reach have no possibility of getting tested for HIV unless symptomatic. For the remainder of the population (not hard to reach), increasing rates of HIV testing (for the 1st time and for repeat testing) since 2009 were assumed (parameter *an_lin_incr_test* sampled at the start of each model run), to reflect the range of levels of testing observed in countries in SSA. In particular, women experience a 50% higher testing rate (*rr_testing_female*=1.5). This increase in testing is assumed to stop at a certain calendar year (*date_test_rate_plateau*), so that testing rates reach a plateau. From mid-2022, it is assumed that the rate of testing declines by a factor obtained as the product of the rate of increase (*an_lin_incr_test*) and *fold_rate_decr_test_future,* up to a minimum equal to the rate of testing in 2011. We assume some targeting of testing such that those having a condomless sex partner since last test are more likely to test – the degree of such targeting is conveyed by the parameter test_targeting. However, the maximum frequency with which a person (non sex-worker) without AIDS or WHO stage 3 disease can test for HIV is determined by the parameter *max_freq_testing*.

HIV testing for pregnant women and prevention of mother to child transmission

HIV testing in antenatal clinics was assumed to be introduced in mid-2003 (*date_start_testing*). Pregnant women experience an additional probability of being tested. This is assumed to occur in all women attending ANCs. The probability of attending ANC (*prob_anc*) increases over calendar time from a value of 0.1 in mid-2003 (when testing in ANC is assumed to have been introduce) up to the value reached in 2015 or when the value of 0.975 is reached. The probability increases in each three month period by *rate_testanc_inc*. Women who attend ANC (this is determined 3 months into the pregnancy) are assumed to have a 50% chance of getting tested for HIV at this time point, 100% chance at 9 months and 33% chance at 3 months after giving birth. This is to reflect the fact that in the Zimbabwe Operational and Service Delivery Manual revised in 2022 it is recommended that HIV-negative pregnant women and lactating women test in the first trimester of pregnancy; if negative that they re-test again in the third trimester or at delivery and then at 6 weeks postnatal and 6 monthly during the breastfeeding period. The re-testing six monthly during the breastfeeding period is not modelled, as there are limited data to inform it.

Prevention of mother to child transmission is assumed to be introduced in 2004 (*date_pmtct*). The probability of receiving PMTCT increases by *pmtct_inc_rate* (0.20) per year up to a maximum of 0.975. Women who attend ANC and have never been initiated on treatment experience this probability. Before mid-2010, nevirapine as a single dose was used in women to prevent mother to child transmission. The rate of acquiring NNRTI resistance in these circumstances if given by *prob_nnresmaj_sd_nvp* = 0.35.

From 2012 nevirapine was used with zidovudine and here *prob_nnresmaj_dual_nvp* = 0.045 (Arrive 2007). If an NNRTI mutation has arisen in these circumstances the rate of loss of the mutation from majority virus is given by *rate_loss_nnres_pmtct_maj* = 0.75. There is evidence of the mutation being lost entirely as responses to subsequent NNRTI based regimens were undiminished so there is a rate of loss entirely of *rate_loss_nnres_pmtct_min* = *rate_loss_nnres_pmtct_maj*.
Since 2012, women giving birth experience an increase chance of starting of re-starting treatment (see section 10.2 on initiation).

HIV testing in people presenting with symptoms

People with acute symptoms (WHO stage 4, 3 or active TB) are assumed to have a higher chance of testing for HIV in that 3 month period and a higher chance of being linked to care once diagnosed. The testing probability for people with WHO stage 4 (*test_rate_who4*) increases over time starting from a value of 0.1. In each 3 month period this is multiplied by *incr_test_rate_sympt* (See Table S27 below), up to a maximum of 0.9. For people with TB (*test_rate_tb*) the same applies but up to a maximum of 0.8 and for people with WHO stage 3 excluding TB (*test_rate_non_tb_who3*) it starts from 0.05 up to a maximum of 0.7.
People with TB without HIV also are assumed to have the same chance of testing as people with TB with HIV (*test_rate_tb)*.
There is also a probability of non-HIV-related symptoms (resembling HIV symptoms) arising in people regardless of HIV status (rate_non_hiv_symptoms=0.005) leading to HIV testing.

HIV testing in female sex workers programmes

Female sex workers can engage in a female sex workers program. Among those who engage, a certain proportion (*effect_sw_prog_6mtest* sampled for each simulation) will test every 6 months (indicated by sw_test_6mthly=1).

Accuracy of HIV tests

The default for HIV tests modelled is 3rd generation antibody tests (used indicated by *hivtest_type*=3). The table below summarize the type of tests considered and their accuracy.

**Type of test**	**hivtest_type**	**Sensitivity (*sens_vct*)**	**Window period**	**Sensitivity during 3 months since infection**	**Specificity (*spec_vct*)**
PCR (RNA VL)	1	0.98	10 days	*sens_primary*: 0.86	1
3rd gen (Ab)	3	0.98	20 days – 45 days	*sens_primary_testtype3*: 0.5 50%; 0.75 50%	0.992
4th gen (Ag/Ab)	4	0.98	20 days	*sens_primary*: 0.75	1

Testing related to PrEP and circumcision is described respectively in the following section and in section 8.

PrEP

Overview of modelling of (oral) PrEP

Use of (oral) PreP

We consider various possible policies by which oral PrEP (tenofovir and lamivudine (which is not distinguished from emtricitabine in the model)) is provided / available. By default, we assume that women and men will only use PrEP during 3 month time periods in which they have condomless sex with at least one short-term partner (*newp*), when they have a long term partner (ep) who is known to have HIV but is not on ART, or when a woman feels there is a high risk her long term partner is in this position (which is implemented as women aged under 50 without HIV and with a long term condomless sex partner who is not on ART having a 5% chance that she will be considered as fulfilling the criteria for *risk-informed* PrEP, which becomes 50% if her partner has HIV, based on the assumption that for women who suspect they are at risk are indeed at substantially higher risk that their partner has HIV). We assume in this default approach that PrEP will not be used at other times (although this possibility is explored in sensitivity analyses, with the approach determined by the parameter *prep_any_strategy*). It is assumed that the aim is for daily PrEP use during such 3 month periods, although we account for < 100% adherence.

While in our main analyses we assume that all PrEP use will be risk-informed as described above, we do not assume that everyone fulfilling the above criteria for risk-informed PrEP will take it. Only a proportion of people are considered willing to take PrEP even when they fulfill the above risk-informed PrEP criteria (variable name: *prep_oral_willing*). Whether *prep_oral_willing* = 1 or 0 depends on the value of the variable pref_prep_oral which represents the degree to which the person would be likely to take oral PrEP should it be indicated. It is sampled for each individual from a distribution beta(*pref_prep_oral_beta_s1*, 5), where *pref_prep_oral_beta_s1* currently = 2. If pref_prep_oral is above a lower threshold (*prep_willingness_threshold*, currently = 0.2) then prep_oral_willing = 1, otherwise it is 0.

The subset of people considered “hard to reach”, as described in section 5, also will not start PrEP.

First (oral) PrEP initiation

People willing to take PrEP and that fulfill the above risk-informed PrEP criteria can start PrEP through two routes: they go for PrEP (and are tested before starting) or they are offered PrEP when testing negative for HIV.

When initiating PrEP through the first route, a parameter *rate_test_startprep_any* represents the additional rate of being tested for HIV because of interested in PrEP in people who have never been on PrEP. If a person is willing to take PrEP (prep_oral_willing = 1) and has tested negative and meets the criteria for risk-informed PrEP and is not “hard to reach” then they will start PrEP in the period with probability 1.

When initiating PrEP through the second route, they will start PreP with probability *pr_prep_oral_b* (with the exception that during the scale-up period (dur_prep_oral_scaleup = 4) lower values are used) if they have never been on PREP and they are not "hard to reach".

(Oral) PrEP continuation

PrEP continuation requires 3 monthly confirmation of HIV negative status in addition to continuing to fulfil the criteria for risk-informed PrEP. We also consider that people may stop oral PrEP despite continuing to fulfil the criteria for risk-informed PrEP. There is assumed to be a probability (*rate_choose_stop_prep_oral*) of discontinuation per 3 months. If this is the case, there is assumed to be a *prob_prep_any_restart_choice* chance of HIV testing and resumption per 3 month period in which the criteria for risk-informed PrEP are met thereafter. If a person stops oral PrEP due to no longer fulfilling the criteria for risk-informed PrEP then PrEP is restarted if the person tests HIV negative and again fulfils the criteria.

(Oral) PrEP efficacy

We assume that oral PrEP has efficacy (*prep_oral_efficacy*) of 90% or 95% (each in 50% of setting scenarios) (Heffron 2018) and the partner with HIV does not carry virus resistant to both emtricitabine/ lamivudine and TDF. Oral PrEP adherence level for an individual, quantified on a scale of 0-1 is the proportion of the drug target level that is attained with condomless sex partners in a given 3-month period (determined by *adh_pattern_prep_oral*). We assume that in 50% of 3- month periods on oral PrEP adolescents and young adults (age 15-24 years) will have an oral PrEP adherence *rel_prep_oral_adh_younger* 0.8 times that for older adults. The assumptions described result on average in a median of 86% of people on PrEP having > 80% adherence. Oral PrEP effectiveness, as opposed to efficacy, is what is measured in real life conditions and is assumed to be proportional (i.e .0.90 or 0.95-fold) to the PrEP adherence level. In a person with current PrEP adherence of 80% when assuming efficacy of 0.95 the current effectiveness would be 0.95 x 80% = 76%. The median effectiveness over setting scenarios 71%. Oral PrEP is assumed to be only 25% / 50% (determined by sampling *oral_prep_eff_3tc_ten_res*) as effective when the partner has virus with both K65R and M184V resistance mutations that affect tenofovir and emtricitabine or lamivudine. (Gibas 2019)

Inadvertent use of (oral) PrEP in people living with HIV and risk of resistance development while on (oral) PrEP

Oral PrEP can inadvertently be used in people with HIV in two situations: (a) due to starting oral PrEP when already infected with HIV or (b) due to people becoming infected while taking oral PrEP.
People can start oral PrEP when already infected with HIV due to <100% HIV test sensitivity. The accuracy of 3rd generation is described at the end of section 6.
Inadvertent use of PrEP in people with HIV can also occur due to them becoming infected while taking oral PrEP (due to sub-optimal adherence, less than 100% PrEP efficacy, or infection with tenofovir/FTC drug resistant virus). There is a risk of resistance emergence for persons who inadvertently take oral PrEP while having (drug

sensitive) HIV (Lehman 2015, Parikh 2016), determined by the same mechanism as if they were on tenofovir/FTC as treatment, except when in primary infection when the risk is directly determined by parameters *pr_184m_oral_prep_primary* (= 0.3) and *pr_65m_oral_prep_primary* (= 0.1). The approach to determining the risk of resistance in people on ART is described in section 0 . In brief, risk of resistance depends on overall activity of the drug regimen being taken (sum of the activity levels of the drugs, accounting for presence of drug resistance mutations), current adherence and current viral load. Resistance risk is highest when the overall regimen activity is low and adherence is moderate or high. For a fully active regimen, resistance risk is highest when adherence is moderate. (see details in section entitled Modelling the effect of ART).

Long-acting injectable PrEP

Recent trials have shown that long-acting injectable cabotegravir (cab-la PrEP), an integrase inhibitor, is highly efficacious in reducing HIV acquisition risk through sex among women and men having sex with men (Delany-Moretlwe et al 2022, Landovitz et al 2021). Assumptions below are informed by data from these and other studies of cabotegravir (e.g. Eshleman 2022 x 2, Marzinke 2021 x 2, Radzio-Basu J 2019). For modelling of cab-la PrEP, parameters *pr_prep_inj_b, prep_inj_efficacy, rate_choose_stop_prep_inj, pref_prep_inj_beta_s1* have the equivalent role as the corresponding parameters for oral PrEP. pref_prep_inj is the degree to which the person would be likely to take cab-la should PrEP be indicated. It is given by sampling from beta(*pref_prep_inj_beta_s1*, 5) for each individual where *pref_prep_inj_beta_s1* is sampled from values of 2, 3, 4, 5, 6 each with equal probability, to reflect uncertainty. When cab-la PrEP is available then a person starting PrEP will start the PrEP type according to which between pref_prep_oral and pref_prep_inj has the highest value. The scale up of cab-la takes *dur_prep_inj_scaleup* = 2 years.

Cab-la administration and risk of resistance

Cab-la PrEP is administered by injection every 2 months (note that our 3 month model time step means that we have to consider periods of cabotegravir use of 3 months at a time which does not reflect the actual time interval) and by definition they have the intended full drug level, except during the tail when it is lower. Because of the drug levels of cab-la (whether taken by a person with HIV as treatment or by a person with HIV on PrEP who does not realise they have HIV) do not depend on human behaviour once they received the injection, the variable adh_dl is used instead of adh. For oral drugs adh_dl is the same as adh was, for those currently on cab-la adh_dl = 1. Integrase inhibitor drug resistance mutations modelled are at the following codon positions on the integrase gene: 118, 140, 148, 155, 263 (https://hivdb.stanford.edu/dr-summary/resistance-notes/INSTI/). A parameter *pr_inm_inj_prep_primary* determines the risk that a person in primary infection while dosed with cabotegravir (due to cab-la PrEP being started when in the primary infection window period in which HIV was not detected, or due to breakthrough infection during cab-la) develops resistance to cabotegravir in this 3 month period of primary infection. After the period of primary infection the risk of resistance is determined as described in the section 0as if cabotegravir was being intentionally used as treatment, with the exception that risk of integrase inhibitor resistance in the 3 month period after primary infection is directly

determined by a parameter *incr_res_risk_cab_inf_3m* (values 1 3 5 10 20 50 to express the high uncertainty). This parameter is included to ensure that we consider fully the potential risk of integrase inhibitor resistance due to starting cab-la when in early infection.

After a person on cab-la PrEP stops the injections their drug level will fall, with a trajectory that can be very gradual. Of interest in the model is the period during which the level remains at a sufficiently high level that if the person becomes infected, there is a substantive risk of drug resistance (integrase inhibitor drug resistance). There is also some ongoing inhibitory effect on viral replication. The length of this period is likely to differ by individuals. We currently consider two scenarios of the pattern of drug level in this "early tail" period (determined by *cab_time_to_lower_threshold_g*).

A parameter *rel_pr_inm_inj_prep_tail_primary* determines the relative risk (compared with *pr_inm_inj_prep_primary* mentioned above) that a person who becomes infected with HIV who is in the early cabotegravir tail period develops resistance to cabotegravir. As above, after the period of primary infection the risk of resistance is determined exactly as described in the section 0 with the "adherence" being the drug level (adh_dl).

Cab-la PrEP is assumed to be less efficacious (parameter *prep_inj_effect_inm_partner*) when the sexual partner with HIV to whom the subject is exposed has virus with an integrase inhibitor resistance mutation.

HIV testing for people on Cab-la PrEP

HIV testing to discern whether HIV is present is particularly challenging in the context of people taking cab-la PrEP, because cabotegravir exerts a strong effect on viral replication so virus levels can be low and antibody levels slow to develop. Rapid 3rd generation antibody tests have particularly low sensitivity when testing people on Cab-la PrEP. For this reason, it has been proposed that it may be necessary to use RNA-based tests in people starting cab-la PrEP to rule out HIV (if variable hivtest_type_1_init_prep_inj=1), and perhaps to also continue using such tests for the regular 3 monthly tests (if variable hivtest_type_1_prep_inj=1). We sample sensitivity of such tests in people exposed to cab-la PrEP. This is determined by the parameter *sens_tests_prep_inj,* which determines the combination of sensitivity of such tests . Each of these involves specification of sensitivity of antibody (referred to as type3) and RNA-based tests (referred to as type1) according to the time since HIV infection (primary, 3-6 months, 6-9 months or more;) in people on cab-LA. Separately we specify the sensitivity of antibody testing in a person with HIV in the cab-la "early tail" (*sens_vct_testtype3_cab_tail*). Due to earlier detection of HIV and more rapid ART initiation, RNA testing for people on PrEP throughout is assumed to lead to (*testt1_prep_inj_eff_on_res_prim* = 0.25 / 0.5 / 0.75 (with equal probability) times the probability of acquiring a given integrase resistance mutation for a person in primary infection while on Cab-LA.

Table S17. HIV test sensitivity in the context of cab-la use. Range of HIV test sensitivity values sampled for various cab-la use situations. See Taylor et al, Eshleman et al 2022 x 2, Marzinke et al 2021x 2.

Cab-la presence	Time since HIV infection	Sensitivity value (or range from which value selected for each model run / setting-scenario)<br>3rd generation rapid tests	RNA-based tests
Not on cab-la	in 3 month period of primary infection	0.5 / 0.75 *	0.86
Not on cab-la	3-6 months	0.5 / 0.75	0.98
on cab-la	within 3-month period of primary infection	sens_ttype3_prep_inj_primary<br>0 / 0.1	0.7 if option=1<br>0.5 if option=2<br>0.3 if option=3<br>0.2 if option=4
on cab-la	3-6 months	sens_ttype3_prep_inj_inf3m<br>0 / 0.2	0.85 if option=1<br>0.7 if option=2<br>0.5 if option=3<br>0.3 if option=4
on cab-la	6 months or more<br>*sens_tests_prep_inj*	sens_ttype3_prep_inj_infge6m<br>0.1 / 0.25 / 0.5	0.95 if option=1<br>0.8 if option=2<br>0.7 if option=3<br>0.5 if option=4
	…in early cab-la tail period	*sens_vct_testtype3_cab_tail*<br>0.5 / 0.8 / 0.98	0.98
Not on cab-la	6 months or more?	0.98	0.98

Note: In this table option refers to the parameter sens_tests_prep_inj which is distributed as Option 1:25%   option 2:25%   option 3:25% option 4:25%

* Since the model updates in 3 month chunks, this value reflects an average of sensitivity across the 3 months (zero sensitivity in initial few days of infection followed by gradual increase over the first month and then high sensitivity in the second and third months).

Male Circumcision

In the model we assumed males have a one off probability of being circumcised using traditional approach (prob_birth_circ, see Table S27 for values from which we sample) before they reach age 15.
Voluntary medical male circumcision (VMMC) is introduced in 2008 (*mc_int*) in young boy and men aged 10 to 49. The probability of VMMC (prob_circ) is influenced by the underlying VMMC rates (*circ_inc_rate,* see Table S27 for values from which we sample), age (*circ_inc_15_19, circ_inc_20_30, circ_inc_30_50*) and calendar year (*rel_incr_circ_post_2013*). The range of probabilities of VMMC according to age and calendar year reflects the range of VMMC prevalence across the region. Men in which the probability of VMMC realize are tested for HIV and if negative receive VMMC. Additionally, there can be the possibility (if `test_link_circ=1`) that uncircumcised men testing negative for HIV will link to VMMC, with probability indicated by *test_link_circ_prob =0.05* . VMMC is assumed to lead to a 60% lower risk of HIV acquisition from a given partner with HIV (Bailey 2007, Auvert 2005, Gray 2012).
In addition, it is assumed circumcision can occur at birth with probability *prob_birth_circ*.

HIV progression in absence of treatment

Figure S3 gives an overview of the modelling of HIV natural history. The model of the natural history of HIV and the effect of antiretroviral therapy has been derived previously and compared with a range of observed data (see Phillips et al Lancet 2008, AIDS 2011, Nakagawa et al 2012, 2015 and associated supplementary material). Below we set out the structure of the model and explain what parameters represent.

Figure S3. Overview of modelling of natural history of HIV infection

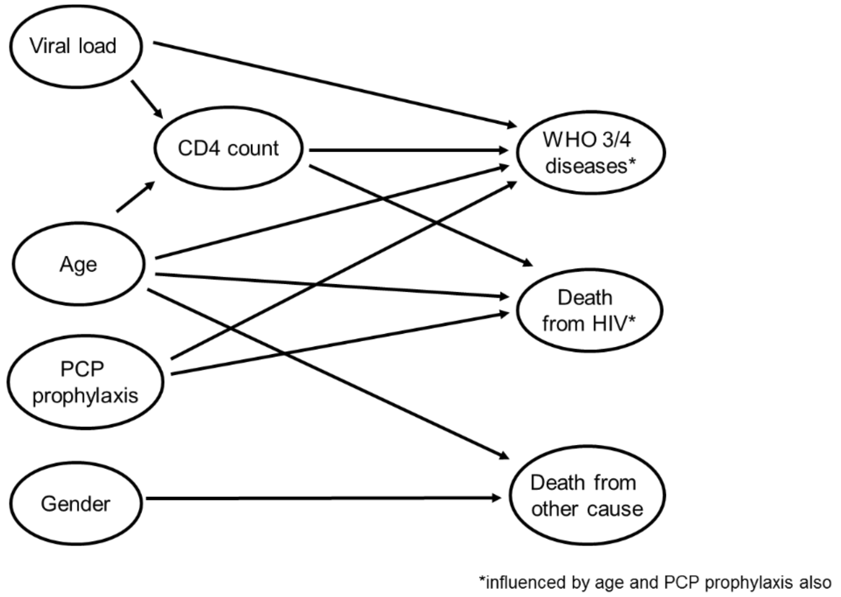

Determination of changes in viral load and CD4 count

Initial $\log_{10}$ viral load (V_{set}) is dependent on age and sex and is sampled from Normal(4.075,0.5^2) + ((age(t)-35)*0.005) for men and Normal(3.875,0.5^2) + ((age(t)-35)*0.005) for men

This viral load (V_{set}) is assumed to be that reached after primary infection. It is not used to determine the risk of transmission in primary infection itself.

Initial CD4 count, modelled on the square root scale, is partially dependent on initial viral load and given by

Square root CD4 count = *mean_sqrtcd4_inf* (= 27.5) - (1.5 x V_{set}) + Normal(0,2^2) – ((age – 35) x 0.05)

Initial virus is assumed to be R5-tropic. Shift to presence of X4 virus is assumed to depend on viral load. Probability of a shift per 3 months is given by 10^v x 0.0000004, where v is the current $\log_{10}$ viral load.

Viral load change (vc) from period t-1 to period t (i.e. in 3 months) is given by

vc(t-1(= (gx x 0.02275 + Normal(0, 0.05^2) + ((age(t-1) - 35) x 0.00075)

gx is sampled as described in Table S27. viral load at t (v(t)) = v(t-1)+ vc(t-1)

CD4 count changes from period t-1 to t are dependent on the current viral load (i.e. viral load at time t-1) and are given by sampling from a Normal distribution with standard deviation *sd_cd4*=1.2 and mean *fx* (sampled as described in Table S27) times the values as follows:

Viral load at t-1	Change in square root CD4 count (per 3 mths)
<3.0	+0.000
3.0-	+0.022
3.5-	+0.085
4.0-	-0.400
4.5-	-0.400
5.0-	-0.850
5.5-	-1.300
6.0-	-1.750

People with X4 virus present experience an additional change in square root CD4 count of -0.25.

These estimates were derived based on consideration of evidence from natural history studies (Pantazis 2005, Sabib 2000, Hubert 2000, O'Brien 1998, Henrard 1995, Lyles 2000, Touloumi 2004, Mellors 1997, Koot 1993) and were selected in conjunction with other relevant parameter values to provide a good fit to the incubation period distribution. Differences that have been found in initial viral load by sex, age and risk group are not currently incorporated in the model.

Table S18. Example model outputs of incubation period by age. Kaplan-Meier percent with WHO 4 Event. Compare with Darby 1996. This varies by model run due to the sampling of the value of the parameter *fx*.

Age at infection	5	10	15	20
15-	10%	30%	38%	39%
25-	16%	36%	43%	43%
35-	21%	42%	48%	51%
45-	28%	46%	55%	50%
55-	35%	53%	56%	100%

If a CD4 count is being measured then the measured value is the underlying value (on the square root scale) plus measurement error (obtained by sampling from a Normal distribution with standard deviation (*sd_measured_cd4*) 1.7.

Modelling the effect of ART

Here we describe details of the modelling in relation to drug resistance and the effect of ART. Before giving full details we show (Figure S4) outputs of the model relating to outcomes by 1, 3 and 10 years from initiation of first line ART with either an efavirenz or dolutegravir based regimen (each with tenofovir and 3TC) in the absence of any switching in drug regimen.

This illustrates the combined effects of the model assumptions which are described below.   This is in the context of adherence profile 2 (see below for different adherence profiles considered), and it is for a situation with which there is no pre-ART NNRTI resistance.

Figure S4. Illustration of assumptions on effectiveness of efavirenz and dolutegravir-containing 1st line regimens. Outcomes at 1, 3 and 10 years in absence of any switching to second line. In the context of adherence profile 2

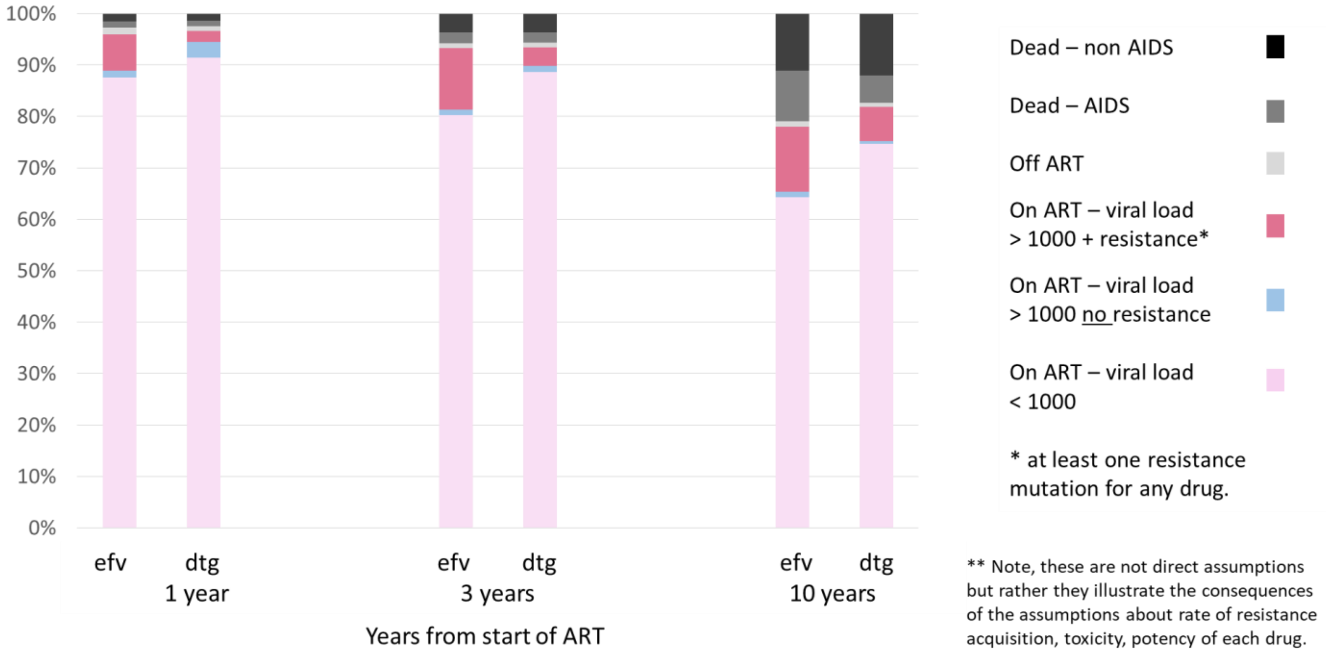

Modelling the effect of ART - Structure

The structure of how the relationship between ART adherence, viral load, development of resistance, CD4 count and risk of death is modelled is illustrated in Figure S5 below. The adherence level - the determination of which is described in detail below - influences the risk of acquisition of new mutations as well as having a direct effect on the viral load and CD4 count. Acquisition of resistance mutations impacts on the total activity level of the regimen, calculated as the sum of the activity level of the drugs, akin to what is sometimes referred as a “genotypic sensitivity score”. This, in turn, is a further determinant of the risk of new mutations arising. Distinction is made for each resistance mutation as to whether it is only present in minority virus (which can occur if the patient has a mutation present but is not taking a drug that selects for that mutation), so the mutation is assumed not transmissible, or if it is present in majority virus. Failure of the current line of ART is determined by CD4 count or viral load or clinical disease, depending on the monitoring strategy being implemented, and this triggers a switch to the next line of ART at a certain rate (*pr_switch_line*). The following sections provide further details, including how adherence levels are determined and how they influence the viral load, risk of resistance and the CD4 count.   We also explain the modelling of ART interruption and loss to follow-up.   We provide references to papers that have been used to inform the approach. It should be noted though that

parameter values used in the model are rarely extracted directly from any one paper, they are values that are arrived at based on their ability to generally reproduce outputs that are consistent with observed estimates.

Figure S5. Overview of the modelling of the effect of ART.

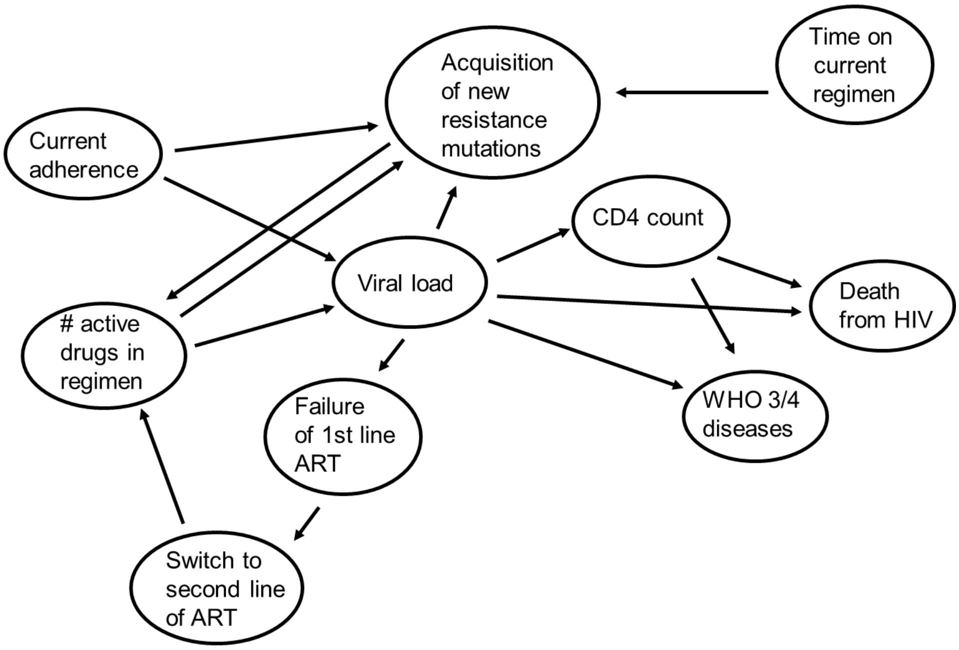

Initiation of ART

It is assumed ART became available in 2004. Eligibility for ART initiation in people diagnosed with HIV before mid-2008 is determined by the development of a WHO 4 or TB event and therefore until this point individuals diagnosed with HIV, not initiated yet on ART, are monitored for the presence of a WHO 4 or TB event (*hiv_monitoring_strategy*=1) . From mid 2008 to mid-2011, eligibility for ART initiation is gradually switched (*rate_ch_art_init_str_4*=0.4; it applies per 3 months) to be determined by a measured CD4 count < 200 (in the last year) or the development of a WHO 4 event or TB; from mid-2011 to 2014 is gradually switched (*rate_ch_art_init_str_9*=0.4; it applies per 3 months [0.1 in Zimbabwe])) so that people is eligible to be initiated on ART if they have a measured a CD4 count <350 or a WHO 4 event. , or if they are pregnant (option B+) . From 2014, ART initiation was gradually switched (*rate_ch_art_init_str_10*=0.4; it applies per 3 months) indicated also based on a CD4 count < 500. Therefore, from mid-2008 to mid-2016 CD4 in individuals diagnosed with HIV not yet on ART is measured every 6 months. From mid-2016 onwards, gradually (*rate_ch_art_init_str_3*=0.4; it applies per 3 months) all people diagnosed with HIV are eligible for treatment and therefore once diagnosed they are started on treatment relatively quickly and we assume there is only monitoring for the presence of a WHO 4 or TB event (*hiv_monitoring_strategy*=1). For people that are eligible to be initiated on treatment the probability that ART initiation occurs is

determined by sampling from a Uniform (0,1) distribution and determining whether this is below the value for *pr_art_init*. The ART initiation policy at any point in time in the model is specified by the variable art_initiation_strategy.

Choice of ART regimen

We model a representative range of specific drugs, but not all drugs. Drugs included are lamivudine (also representing FTC/emtricitabine), zidovudine, tenofovir (no distinction between TDF and TAF), nevirapine, efavirenz, atazanavir/r, lopinavir/r, darunavir/r, dolutegravir and cabotegravir (with the latter only used as long-acting PrEP). Regimen choice at a given point in time is largely reflective of WHO guidelines at the time. For several years until 2017 there has been a recommendation to use efavirenz/tenofovir/lamivudine. With increases in NNRTI drug resistance there has been a recent WHO recommendation to initiate ART with a first line regimen of dolutegravir/3TC/tenofovir (initially this applied only if pre-treatment NNRTI resistance levels were above 10%, but from 2019 is a general recommendation). There has not been a WHO recommendation to switch efavirenz to dolutegravir in people already taking efavirenz, except in the context of detected virologic failure. However, several countries have in fact switched nearly all people on ART to dolutegravir/3TC/tenofovir. We sample for each model run a parameter to determine whether there has been such a complete switch to dolutegravir, or only for ART initiators (*reg_option_104:* 50% probability of each).

Monitoring of people on ART

Early guidance from WHO for monitoring of people on 1st line ART and deciding whether to switch to second line ART included monitoring for occurrence of new clinical conditions or regular measurement of the CD4 count to detect if this was declining. From around 2015 the recommendation changed to use of viral load monitoring. Our model reflects these changes, we assume clinical monitoring before 2016. The monitoring strategy in use at any point in time is conveyed by the variable art_monitoring_strategy. If viral load monitoring is in place a parameter *prob_vl_meas_done* determines the probability that any measure due is done as intended.

Switch to second line after failure of first line ART

The probability of switching per 3 month period after the criterion for failure of first line ART is met is *pr_switch_line*. The switch rate is likely to vary substantially by setting (Fox 2012, Johnston 2012, Rohr et al, Narainsamy et al, Petersen et al, Ramadhani et al, Murphy et al). The 2nd line regimen has tended to consist of a boosted protease inhibitor, lamivudine and zidovudine. Recently dolutegravir has been used as part of 2nd line regimens in people who had not previously experienced this drug. There is not a

consensus on whether the 2nd line regimen should replace tenofovir with zidovudine or not. As discussed above, we aim to reflect this variability.

Adherence pattern

The model specifies a current adherence level (i.e. for the current 3 month period) for people on ART, a value between 0%-100%. We first give a brief description of the approach and then give further detail. Since the model updates in 3 month periods, short term interruptions of days or a few weeks are treated as sub-optimal average adherence during the 3 month period. Interruption of ART over periods of 3 months or greater are referred to as ART interruption/discontinuation and modelled explicitly. ART interruption/discontinuation is usually concomitant with disengagement from clinic attendance. Average adherence in each 3 month period for an individual is determined from the underlying tendency to adhere (which is a lifelong value for the individual, unless changed as a result of an adherence intervention) with within-person period-to-period variability. Each patient thus has a certain higher or lower tendency to adhere but their actual adherence varies over time, both at random and according to factors such as age, gender, presence of symptoms and experiencing an enhanced adherence intervention as a result of a viral load measured > 1000 copies/mL, as detailed below. Effects of adherence on viral load and resistance acquisition risk are modelled by classifying levels into < 50%, 50-79%, ≥ 80%, with effects of ART on viral load suppression being maximal in the ≥ 80% adherence level (Cheng 2018) and the resistance acquisition risk being highest in the 50%-79% category. We do not distinguish between patterns of adherence at a level more granular than the 3 monthly average level and hence cannot explicitly take into account the specific pattern within the 3 month period, which could be important (e.g. whether 80% adherence consists of missing drug one day in every five or a 1 week interruption in every 5 weeks). Thus the adherence level in each period should be conceived of as conveying the degree to which the pattern of adherence means that drug levels are maintained at intended therapeutic levels, rather than simply the average adherence over the period. The distribution of adherence levels was primarily determined by the adherence levels required for the model outputs to mimic observed data. This includes data on rates of resistance development and virologic failure and also data on the proportion of patients at first virologic failure who have no resistance mutations present (Fox 2008, Bangsberg 2004, Bangsberg 2006, Bangsberg 2006, Hamers 2011, Hassan 2014, Hoffmann 2009, Hoffmann 2014, Kobin 2011, Li 2014, Mackie 2010, Rosenblum 2009, Tran 2014, Usitalo 2014, von Wyl 2013, Johannesen 2009, Musengimana 2022, Agegnehu 2022)

Consistent with evidence that people tend to have different tendencies to adhere, adherence is modelled using two components. Each patient has a certain greater or lesser tendency to adhere (*adhav,* measured on a scale of 0-100%) but, as described above, their actual adherence in a given period varies over time. Adherence in a given 3 month period is referred to as *adh*. *adhvar* is the standard deviation representing the

within-person period-to-period variability over time. Thus, adherence at any one period is initially determined as follows (although with modifications explained below):- adh = *adhav* + Normal(0,*adhvar*^{2}). An example of how the the distribution of the values of *adhav* and *adhvar* are specified as follows and as illustrated in Figure S6. We consider a range of such patterns and sample at random from the distribution of *adh_pattern* for each model run. The different adherence profiles from which we sample are as follows:

1

1% probability	*adhav* = 10%	*adhvar* = 20%
1% probability	*adhav* = 79%	*adhvar* = 20%
18% probability	*adhav* = 95%	*adhvar* = 5%
80% probability	*adhav* = 95%	*adhvar* = 2%

2

3% probability	*adhav* = 10%	*adhvar* = 20%
3% probability	*adhav* = 79%	*adhvar* = 20%
14% probability	*adhav* = 90%	*adhvar* = 6%
80% probability	*adhav* = 95%	*adhvar* = 5%

3

5% probability	*adhav* = 10%	*adhvar* = 20%
7% probability	*adhav* = 79%	*adhvar* = 20%
8% probability	*adhav* = 90%	*adhvar* = 6%
80% probability	*adhav* = 95%	*adhvar* = 5%

4

5% probability	*adhav* = 10%	*adhvar* = 20%
10% probability	*adhav* = 79%	*adhvar* = 20%
27% probability	*adhav* = 90%	*adhvar* = 6%
38% probability	*adhav* = 90%	*adhvar* = 5%
20% probability	*adhav* = 95%	*adhvar* = 5%

5

15% probability	*adhav* = 10%	*adhvar* = 20%
15% probability	*adhav* = 70%	*adhvar* = 20%
50% probability	*adhav* = 90%	*adhvar* = 6%
20% probability	*adhav* = 95%	*adhvar* = 5%

6

20% probability	*adhav* = 10%	*adhvar* = 20%
20% probability	*adhav* = 79%	*adhvar* = 20%
40% probability	*adhav* = 90%	*adhvar* = 6%
20% probability	*adhav* = 95%	*adhvar* = 5%

7

30% probability	*adhav* = 10%	*adhvar* = 20%
30% probability	*adhav* = 60%	*adhvar* = 20%
10% probability	*adhav* = 70%	*adhvar* = 6%
30% probability	*adhav* = 90%	*adhvar* = 5%

Figure S6. Illustration of adherence pattern assumptions. This is for adherence pattern 2. 3% of the population have the adherence as shown in the top left, 14% as shown in the bottom left right, etc. While adherence is generally high in the majority of people on ART (hence the high proportion of people on ART with viral suppression), most probably experience at least some periods of poorer adherence (e.g Muyingo, 2008)

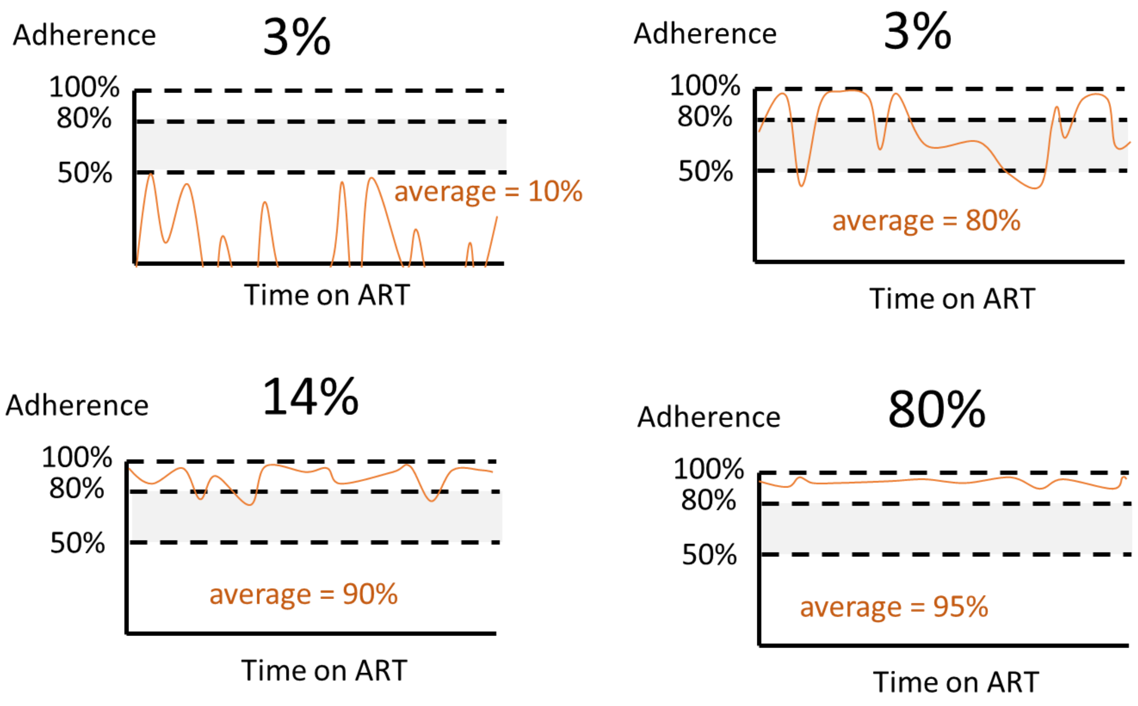

The above describes how we determine the person-specific underlying adherence (*adhav*) and variation in adherence (*advar*). *adhav* remains constant over a person's lifetime, with the exception that it can increase as a result of an adherence intervention due to viral load measurement above 1000 cps/mL (see below), or when starting 2nd line ART (due to the fact that there is the emphasis that this is likely the last line of ART available – a last chance to be adherent, albeit that this effect can be counteracted by the lower adherence described below due to toxicity of atazanavir). The increase in *adhav* when starting second line is person-specific and given by 5% x exp(Normal(0, 1)).

Given *adhav* and *adhvar*, the adherence in any one period is determined as follows. First adh = *adhav* + Normal(0,*adhvar*^{2}). Then adh can be modified according to presence of a drug toxicity, a current WHO stage 4 condition or TB, or gender and age.

Effect of current drug toxicity and current TB or WHO stage 4 condition on adherence

The effect of drug toxicity on adherence is person-specific and given by 5% x exp(Normal(0, 0.3)). In any one 3 month period in which a toxicity is present there is

30% chance of this effect operating. The effect of drug toxicity on adherence is most clearly seen in randomized double-blind trials in which, for example, discontinuation rates are higher for efavirenz-based 1st line compared with a dolutegravir-based regimen (Walmsley 2013, Walmsley 2015). During the time of a WHO stage 4 or TB disease, adherence is assume to be reduced by 10%..

Effect of age and gender on adherence

There is an effect of age on adherence, partly evidenced by differences in viral suppression levels, most notably with lower adherence in the 15-20 year age group compared with older ages (O'Connor 2016, Filimão 2019, Haberer 2019, Ligeois 2019, Jiamsakui 2017, Malawi MoH). Gender is also an influence, as suggested by the higher proportion of women on ART with viral suppression in the PHIA surveys (PHIA surveys).

For men age 15-19 / 20-24 / 25-29, if initially there is adh_dl in a period above 80% there is a 30% / 20% / 10% chance of the adherence being reduced: 65% in two thirds of men and 10% in one third.

For women age 15-19, if initially there is adh_dl in a period above 80% there is a 20% chance of the adherence being reduced: 65% in two thirds of men and 10% in one third. For women age 20-24 / 25-29 / 30-34 / 35--49 / 50+, if initially there is adh_dl in a period below 80% there is a 10% / 30% / 50% / 80% / 90% chance of the adherence being increased to 90%.

Comparisons between model outputs and data from the literature in Figure S7-S13 illustrate the extent to which the model captures various aspects of virologic responses to ART (efavirenz based regimens).

Figure S7. Model outputs of the risk of virologic failure while on ART according to adherence level

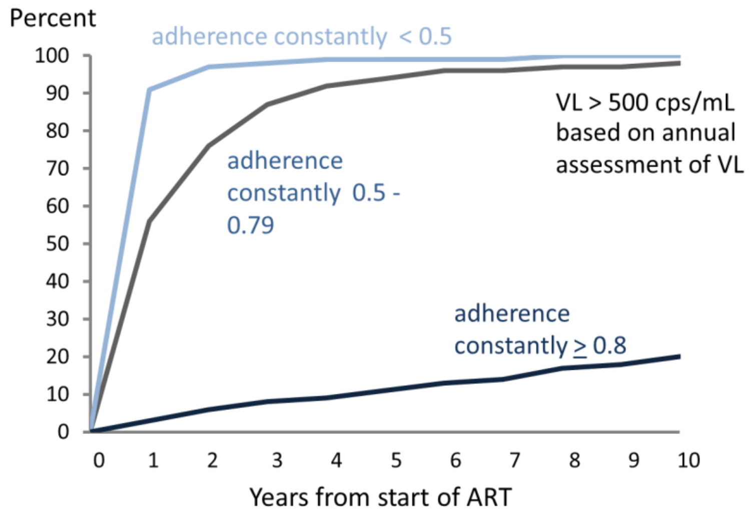

Figure S8. Model outputs of the risk of NNRTI resistance with virologic failure while on ART, according to adherence level

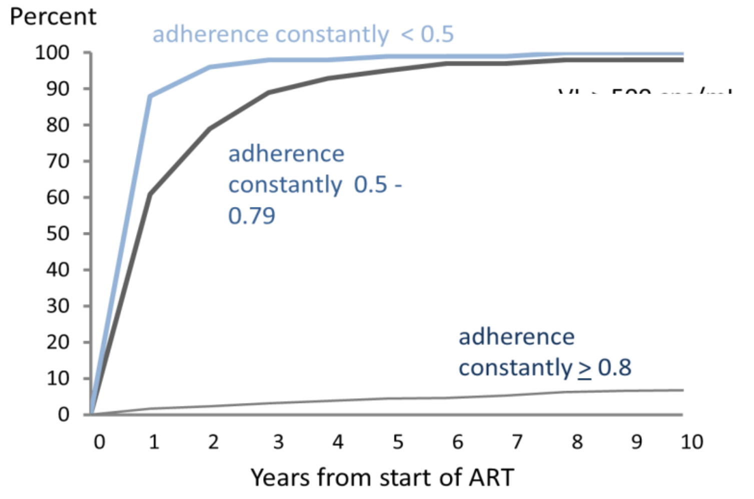

The distribution of adherence over the first year of ART has been compared with data from a large programme in Zambia (see Figure S9; (Chi 2009)). Viral load suppression at one year from start of ART is shown in Figure S10. These are reconstructed outcomes for all people who have initiated ART in Zimbabwe (the overall mean CD4 count at initiation is 145 /mm^3). Figure S11 and Figure S12 compare Kaplan-Meier estimates of time to virologic failure and resistance, respectively, between the model and observed data, in the latter case from the UK due to the lack of data from sub-Saharan Africa (although noting that a substantial minority of people in the UK database originate from sub-Saharan Africa). Figure S13 illustrates the proportion of people with resistance

(amongst those on ART with non-suppressed viral load) and corresponds to estimates from the large WHO resistance surveillance.

Figure S9. Distribution of average adherence level over first year of ART (for those on ART at 1 year) (Chi 2009).

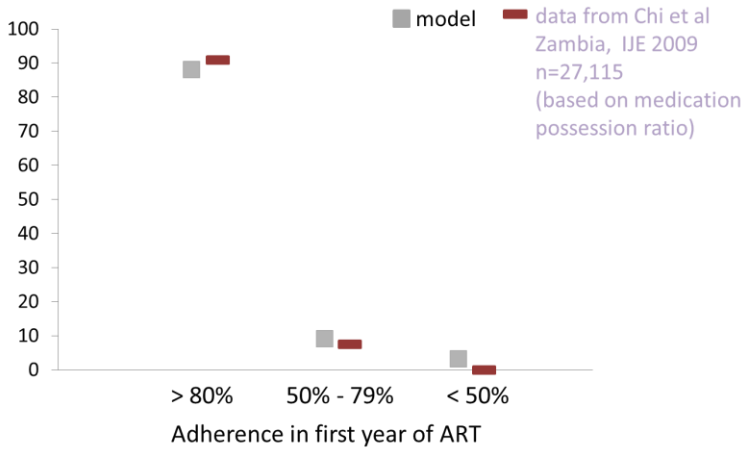

Figure S10. (a) Percent of people alive at given time points from start of ART who have viral load suppression and (b) percent of people alive and on ART at given time points from start of ART who have viral load suppression (WHO resistance report 2012).

(a)

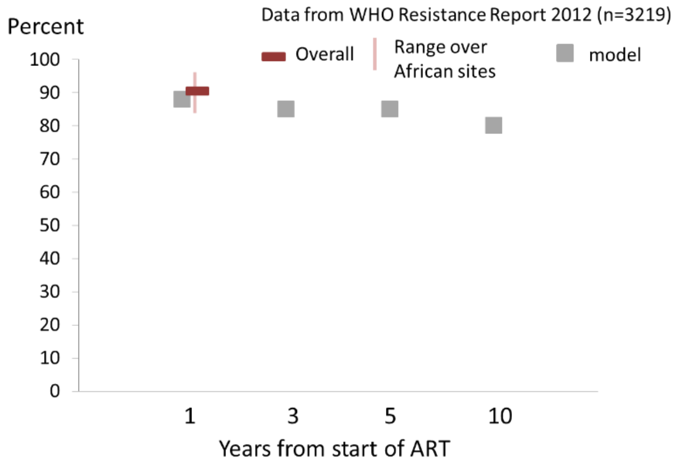

(b)

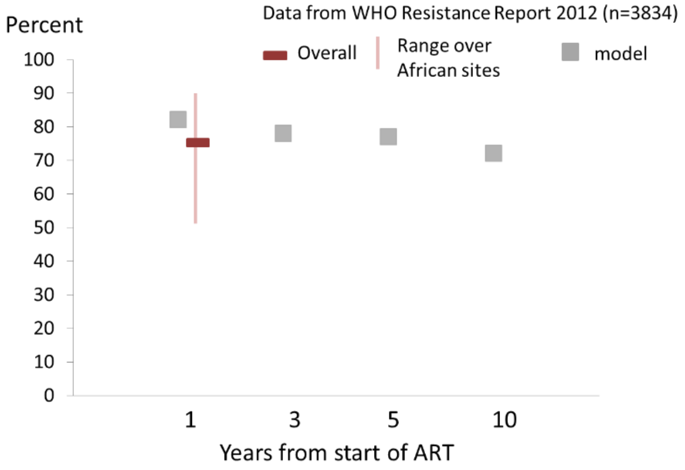

Figure S11 Kaplan Meier estimates of risk of virologic failure while on ART, by time from start of ART (Fox 2012).

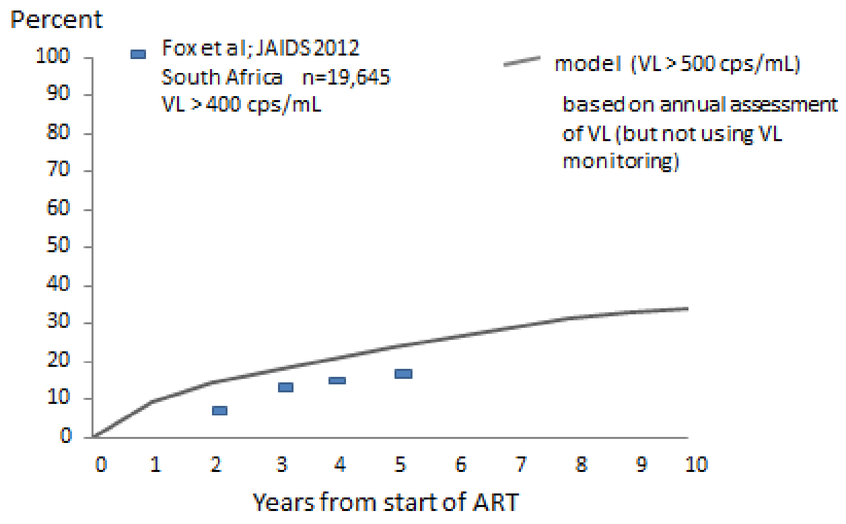

Figure S12. Kaplan Meier estimates of risk of NNRTI resistance with virologic failure while on ART, by time from start of ART (Cozzi-Lepri 2010).

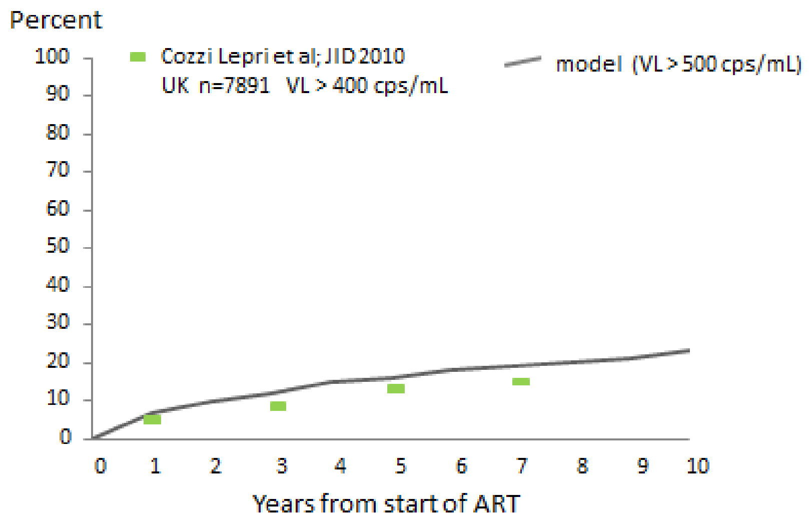

Figure S13. Of people with viral load > 500 at 1 year from start of ART, percent who have NNRTI drug resistance (WHO resistance report 2012).

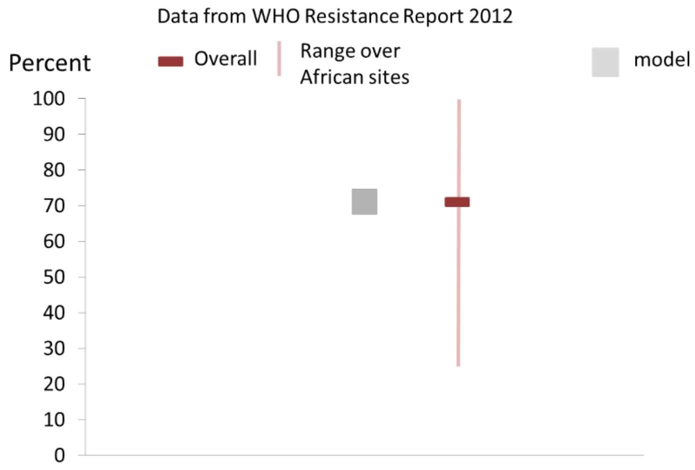

Effective adherence

We also considered the concept of *effective* adherence, which reflects predicted adequacy of drug levels, whereby for those on regimens that do not include an NNRTI the effective adherence is as the adherence itself, but for those on NNRTI-containing regimens the effective adherence is the adherence + *add_eff_adh_nnrti* (base value Log normal(ln 0.10, 0.30)), reflecting the long half life of NNRTI drugs (Cheeseman 1993) which is an advantage as it means such regimens are more forgiving of periods of poor adherence (Bangsberg 2004, Bangsberg 2006, Bangsberg 2006, Kobin 2011, Gardner 2009, Gross 2008, Meresse 2014, Parienti 2007). Additionally,-it is assumed that patients on ART are susceptible to occasional (rate 0.02 per 3-months severe temporary drops in drug level (i.e. effective adherence level), leaving them susceptible to viral rebound (but with low risk of resistance as the effective adherence drop is so profound). This phenomenon is assumed to be 100 times more frequent among those on protease inhibitor regimens than in those on other regimens. This latter assumption is the only plausible means (at least within our model framework) to explain why virologic failure occurring on boosted protease inhibitor regimens often occurs in the absence of resistance (Hill 2013).

Effect of viral load measurement above 1000 cps/mL on adherence

As mentioned, adherence can be affected by experience of an enhanced adherence intervention after initial measurement of viral load > 1000 copies/mL which is assumed to lead to an increase in adherence in 70% of people, consistent with data showing that a significant proportion of people with measured viral load > 1000 copies/mL who undergo an adherence intervention subsequently achieve viral suppression without a change in ART (Hoffmann 2009, Hoffmann 2014, Orrell 2007, Rutstein 2015) and broadly consistent with a meta-analysis (Bonner 2013). Although the appropriate duration to assume for this effect is uncertain (Hoffmann 2014), the impact of

adherence interventions has often been shown to diminish with time (Bärnighausen 2011). Based on this overall body of data, we assume that the adherence intervention is effective only the first time it is performed and that for 40% the effect is permanent (i.e. 70% x 40%= 28% of those with a viral load >1000; in this case the value of *adhav* is reduced from this point), but that in the remaining 60% (i.e. 70% x 60% = 42% of those with viral load>1000) it lasts only 6 months.

ART interruption / discontinuation

People can interrupt ART, and this may be due to not continuing with clinic visits (disengagement, modelled as simultaneous interruption and loss to clinic follow up) but ART can be interrupted also in those still attending clinical visits. The basic rate of interruption due to patient factors (referred to as *rate_int_choice*, although recognising that this is often not a free choice) is greater in people with current toxicity (2-fold) and those with a greater tendency to be non-adherent (1.5-fold if adherence average *adhav* 50 – 79% and 2-fold if adherence average *adhav* < 50%). In a systematic review, drug toxicity, adverse events and side effects have been found to be the most commonly given reasons for drug discontinuation (Kranzer 2011, Dear 2022).
On the other hand, women who gave birth in the last 3 months are assumed 100 times less likely to interrupt.

The rate of interruption also reduces with time on ART, decreasing after 1 years (Kranzer 2011, Kranzer 2010, Tassie 2010, Wandeler 2012, Dear 2022). If adherence average (*adhav*) $\geq$ 80% then the chance that interruption coincides with interrupting/stopping visits to the clinic is equal to *prob_lost_art*; if 50 <= *adhav* < 80% then *prob_lost_art* is multiplied by 1.5, if *adhav* < 50% then *prob_lost_art* is multiplied by 2. This is due to an assumption that factors leading to poor adherence are also likely to be associated with interruption. The rate of interruption and disengagement with care is likely to vary by setting. Figure S14 shows a comparison between modelled and observed (from a study by Kranzer et al 2010). Kaplan Meier estimates of the percent of people having interrupted or discontinued ART by time from ART initiation.

Figure S14. Percent who have interrupted or discontinued ART by time from initiation (87).

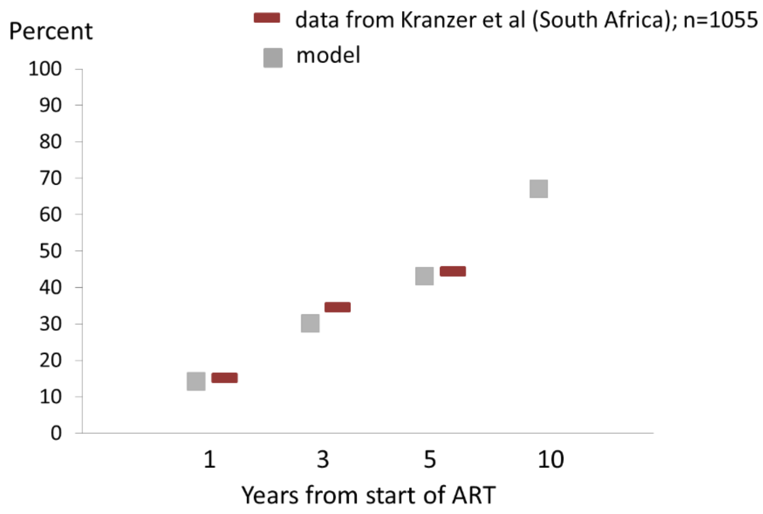

Interruption of ART without clinic being aware

It is known that in some instances people on ART have such poor adherence that they have in fact interrupted or stopped ART entirely but, in the same way that the clinic is not always aware of the true adherence level, they are also not always aware when the person has completely interrupted ART. This means that the clinic may think a patient is virologically failing, because viral load is high, when in fact this is due to interruption rather than resistance. This can be seen from studies on people with virologic failure in which a proportion have no identified resistance mutations (Hamers 2011, Wallis 2010). Thus, when a person interrupts ART (but remains under care) we introduce a variable that indicates whether the clinic is unaware. *clinic_not_aw_int_frac* (base value Beta (6,4), median=0.61). This distribution was chosen to produce realistic model outputs for the proportion of people with detectable viral load who have resistance. If a patient has interrupted ART with the clinic unaware then not only is the patient (wrongly) classified (by the clinic) as virologically failing (if viral load has been measured), but a switch to second line can occur. Figure S15 compares the proportion of people with resistance between our model and WHO survey data.

Re-initiation of ART after interrupting in patients still under clinic follow-up

For patients who have interrupted ART due to "choice" but are still under clinic follow-up, the probability of restarting ART per 3 months in the base model is *rate_restart*. This probability is increased 3-fold if a new WHO 3 condition has occurred at t-1, and 5-fold if a new WHO 4 condition has occurred at t-1 since occurrence of clinical disease in a person seen at clinic is likely to prompt ART re-initiation and 5-fold if they gave birth in the last three months. This will vary by setting but is informed by studies showing that of people who have initiated ART who are still seen at clinic a very high proportion are

on ART at 12 months from start of ART (McMahon 2013). Kranzer et al found a rate of restarting ART amongst those that interrupted or discontinued of 21 per 100 person-years but this figure is an overall figure which includes in the denominator those who are not attending the clinic (loss to follow-up and return to care are described below) (Kranzer 2010).

Interruption due to drug stock-outs

The basic rate of interruption due to interruption of the drug supply is *prob_supply_interrupted* per 3 months. This will vary over time and by setting but we assume low rates in current and future years (0.003 per 3 months per person). For patients who have interrupted ART due to interruption of supply the probability of restarting ART per 3 months is *prob_supply_resumed* (McMahon 2013).

Loss to follow-up while off ART (for reasons apart from drug stock-outs)

The probability per 3 months of interrupting/stopping clinic visits (i.e. being lost to follow-up) is *rate_lost* if adherence average *adhav* ≥ 80%. This is increased by 1.5 fold if 50% ≤ *adhav* < 80% and by 2-fold if *adhav* < 50%. This high rate is informed by the fact that low numbers of people attending clinics after having been initiated on ART are not still on ART (e.g. WHO resistance report 2012). Interruption of ART and loss to follow-up are assumed correlated with the underlying tendency to adhere when on ART because we assume that the same underlying social, practical and economic factors will be an underlying cause of these behaviours.

For people lost to follow-up who are asymptomatic, the probability of returning to clinic per 3 months is *rate_return* if adherence average *adhav* ≥ 80%. This is decreased by 2-fold if 50% ≤ *adhav* < 80% and by 3-fold if *adhav* < 50%. If a person develops a new WHO 3 or 4 event then they are assumed to return to the clinic with probability 1. As mentioned above, this leads to an overall rate of restarting of ART after interruption (including having been loss to follow-up in many cases) consistent with the estimates from South Africa from Kranzer et al, although these will vary by setting (Fox 2012, Gross 2008, Charurat 2010).

Figure S15. Status at 1 year from start of ART. Data is from WHO Drug Resistance Surveillance Report (2012) (74).

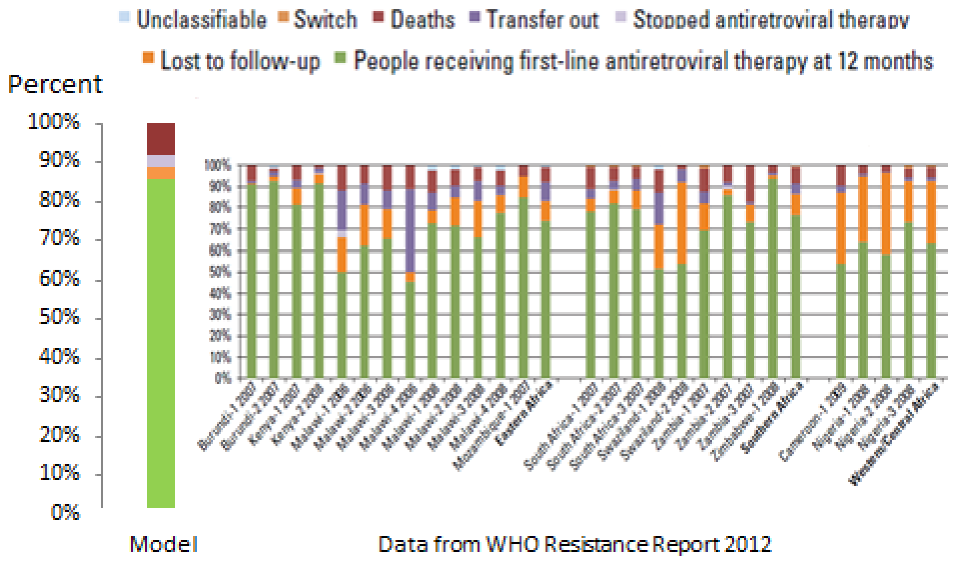

Effect of ART on viral load, CD4 count, resistance development and drug toxicity

This section describes the determination of updated viral load, CD4 count, and acquisition of new resistance mutations in a given time period for people on ART. The updated viral load, CD4 count and risk of new resistance mutations appearing all depend on the effective adherence in the previous and current period, the number of active drugs (*nactive*) and the current viral load, as well as the time period from the last time ART was started or restarted. The values of viral load, CD4 count, and resistance mutation risk for any combination of these factors are given in Table S19-S21 below. The rationale behind this approach and how the specific values in the table were chosen is explained below. The choice of values is directly informed by studies in this area and by comparison of model outputs with data. For the new resistance mutation risk, the number in the table is multiplied by the viral load (mean of values at t-1 and t) to give a value for the variable *newmut*, which is used when assessing whether a new mutation or mutations have arisen (see below).

Number of active drugs

We use the concept of the number of drugs that are active, based on presence of resistance mutations to the drugs being used. The level of resistance is determined by the presence of drug resistance mutations, with a given set of mutations being translated into a level of resistance to a given drug on a scale of 0 to 1 in the same way as is done for common resistance interpretation systems. The activity level of a drug is

then calculated as 1 minus the level of resistance to the drug. The ability of the number of active drugs, or the genotypic sensitivity score, to predict the viral load outcome is well established (DeGruttola 2000), and the concept of using a genotypic score to define "optimised background therapy" has been common to the design of several trials in treatment experienced patients (e.g. Grinsztejn 2007). While some studies of NRTIs have suggested that this relationship does not hold, these are confounded by adherence level (Paton 2017). This is the basic concept but note that below we explain consider that drugs, such as boosted PIs, can have higher potency (since they can virtually sustain viral suppression alone) and thus contribute a value greater than 1.

Classification of adherence levels

While we model the adherence level for each individual at each three month time period as a value between 0 and 100%, to determine the viral load, CD4 count and resistance risk, as noted above, we classify adherence into three levels. This is the simplest approach that allows inclusion of the fact that the relationship between adherence and resistance risk is not linear, since the risk of resistance tends to be lower when the adherence is either low or high, and the risk of resistance is highest when adherence is moderate, allowing enough replication for mutations to occur and enough drug present to allow selection of virus with resistance mutations (Bangsberg 2004, Gardner 2009, Rosenbloom 2012).

As mentioned, the cut-offs used to define the three adherence levels are 50% and 80%. Adherence-resistance and adherence-viral load relationships differ by regimen type and even specific regimen within a class and any overall breakdown into groups is necessarily a simplification. A cut off of 80% is chosen as the upper level as (unlike for unboosted PI regimens) at adherence levels of at least 80%, NNRTI, integrase inhibitor, and boosted PI regimens are likely to have maximal or close to maximal effects on viral load and minimal risk of resistance selection (Parienti 2007, Cheng 2018). Actual risk of resistance probably depends on the pattern of adherence, not just the average over a three month period, so that a treatment interruption of over 1 week during the three month period, while maintaining an overall average adherence of 80%, could lead to a higher level of risk of resistance emergence than a situation in which the adherence was more uniform over the period (Genberg 2012), although in people who have ongoing viral suppression NNRTI regimens seem to be generally robust to even relatively low levels of adherence (Gross 2008, Meresse 2014, Parienti 2007, Cambiano 2010). A level below 50% is one that that has been associated with raised risk of detectable viral load (Genberg 2012, Arnsten 2001)

Determination of viral load, CD4 count and risk of resistance in people on ART

Viral load, CD4 count and risk of resistance in the first 3 months after (re-)starting ART

Table S19 shows how the viral load, CD4 count and risk of resistance is determined for people in the first 3 months after starting ART or re-starting ART after an interruption of at least 3 months. Since in this early period on ART, the viral load will depend on the initial value the updated viral load is given as a reduction from the pre-ART maximum viral load. If the number of active drugs is three or more then at a high adherence level (above 0.8) the mean viral load change from the pre-ART maximum is 3 log copies/mL. To reflect the fact that there is variability in the response (Montaner 1998), the value for a given person is sampled from a Normal distribution with standard deviation 0.5. This viral load response diminishes both with decreasing number of active drugs in the regimen being started (which is informed by data from studies relating GSS to virologic outcome, as well as by studies of mono and dual therapy regimens (DeGruttola, Eron 1995, Havlir 1995, Kuritzkes 1996, Larder 1995, Phillips 1997, Wittkop 2011, Wittkop 2013). The viral load response also diminishes with decreasing level of adherence (see Figure 16 and for example Genberg et al). As is well established, the CD4 count response generally mirrors the viral load response, although with very low numbers of active drugs and low adherence there is a mean decrease in CD4 count and still a small decrease in viral load from the maximum.   Note that we do not incorporate the known more rapid decline in viral load seen with integrase inhibitors.

Figure S16. Model output: of people on ART, percent with current VL >500 according to current adherence.   Comparison with data from Genberg at el on electronic monitoring-based adherence measures (Genberg 2012).

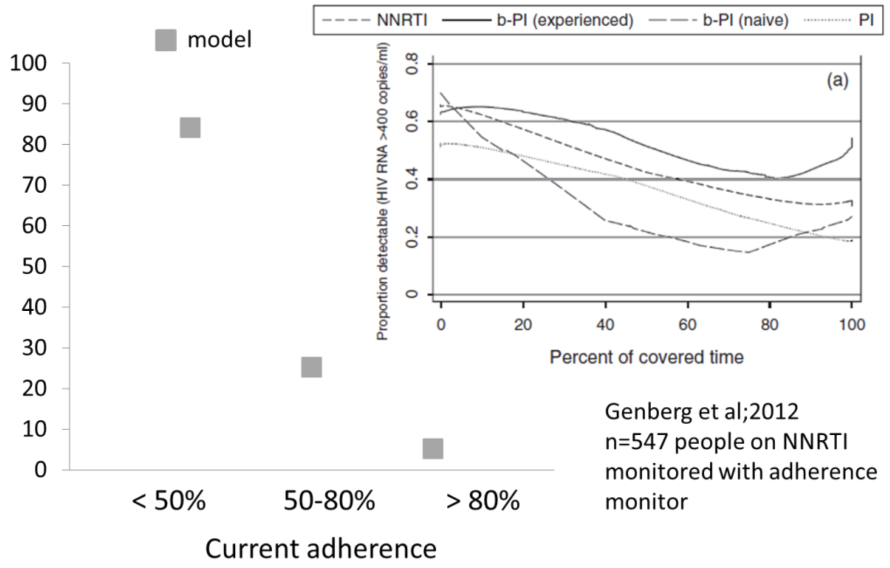

Regarding the risk of new drug resistant mutations arising, Tables S19-S21 provide a number for “new mutation risk” that is multiplied by the viral load (mean of values at t-1 and t) to give a probability used when assessing whether a new mutation(s) has/have

arisen. Values of the new mutations risk have been chosen in conjunction with the translation of presence of mutations into reduced drug activity to provide estimates of resistance accumulation consistent with those observed in clinical practice (Johannessen 2009, Gallant 2004, Harrigan 2005, Ledergerber 1999, Phillips 2001, Phillips 2005, Staszewski 1999).
Risk of new resistance mutations arising increases with decreasing number of active drugs, reflecting the known greater risk of resistance with regimens less able to suppress viral replication, most clearly seen in the fact that early mono and dual therapy regimens were highly susceptible to resistance development (Charurat 2010, DeGruttola 2000, Grinsztejn 2007). At low adherence levels, the risk of resistance development is generally low regardless of the number of active drugs, as drug selection pressure is low. However, for those on NNRTI regimens the new resistance mutation risk is assumed to be that for the effective adherence category of 50 – 80% (i.e. maximal) even if the effective adherence is below 50%, reflecting the fact that NNRTI resistance develops easily, even when drug exposure is very low (Bangsberg 2004, Bangsberg 2006).

Viral load, CD4 count and risk of resistance between 3-6 months from (re-)starting ART

For the period 3-6 months from (re-)start of ART (Table S20; to reduce the table content we do not provide the matrices of values for the resistance risk or CD4 count, only for the viral load (the full table is available in Cambiano et al 2014 (Van Leth 2004). We consider the adherence in both the current and previous 3 month period, since the likelihood of reaching viral suppression by 6 months will depend on adherence throughout the whole 6 month period from start of ART, although the adherence in the current period is assumed to be the stronger factor. By 6 months after starting ART, those on 3 or more active drugs with consistently high adherence generally reach a relatively high level of viral suppression, regardless of pre-ART maximal viral load, so a person's viral load is no longer given by the change from baseline but the absolute level of viral load which it is likely they have reached. In these optimal conditions of high adherence and maximal active drugs we assume the viral load has a mean value of 0.5 log, again with variability between individuals. Since most viral load assays have a lower limit of quantification of 40 or 50 copies per mL, it is not actually known what the viral load level is, although highly sensitive assays suggest that a proportion of patients reach below 11 copies/mL. At lower numbers of active drugs and lower adherence, the viral load is still related to the maximal pre-ART viral load rather than being an absolute value, as the person's viral load has not become so low that the initial value loses relevance. The viral load response decreases with a lower number of active drugs, lower current adherence, and lower adherence in the previous 3 month period. Values for the viral load response between those known from studies (high level of suppression for 3 active drugs and maximal adherence, and only around 0.5 log viral suppression when adherence is < 0.5 even with three active drugs (Phillips 1997, Ruggiero 2018) are imputed assuming a monotonic relationship. CD4 count responses again mirror the

viral load response, as has been extensively studied in patients with ongoing viraemia on ART (Gross 2001). Risk of new resistance mutations again increases with decreasing number of active drugs, if current adherence is in the middle or highest group. The only situation in which risk of new mutations is extremely low is when the number of active drugs is 3 or above and the current adherence is in the high category.

Viral load, CD4 count and risk of resistance after 6 months of (re-)starting ART

Table S21 shows how the viral load, CD4 count and risk of resistance is determined for the situation where a person has been on ART for more than 6 months and the viral load is suppressed or partially suppressed (< 4 log copies/mL). These values are similar to those used for the period 3-6 months from start of ART except that there is assumed to dependence on the adherence in the current 3 month period only.

The situation where the viral load is above 4 log copies /mL, 10,000 copies/mL is treated the same as that in the period 3-6 months from start of ART (described above), with adherence in the current and previous period having some influence.

Variable patient-specific tendency for CD4 count rise on ART

There is variability in the tendency for the CD4 count to rise on ART, for a given level of viral load suppression. For scenarios in the above Tables (S19 – S21) in which the CD4 count change is positive the CD4 count change is multiplied by this patient-specific factor (i.e. it is fixed for each patient), which is given by sampling for each patient from Exp (N(0, $(sd\_patient\_cd4\_rise\_art)^2$) where sd_patient_cd4_rise_art = 0.2. To reflect the fact that the rate of CD4 count increase on ART tends to diminish with time, for those with patient-specific factor determining the CD4 count rise on ART > 1, this factor is divided by 1.25 after 1 year of continuous treatment, by 3 after 3 years of continuous treatment and by 10 after 5 years. In addition there is a dependence of CD4 increase on current CD4 level with a multiplying factor of 0.85-fold if the CD4 count is 100-200 and 0.7-fold if > 200. Comparisons of resultant outputs with observed data are described below.

Accelerated rate of CD4 count loss if PI not present in regimen

The rate of change in CD4 count in people on failing regimens is largely based on data from the PLATO collaboration, for which patients were mainly on regimens containing a PI (Ledergerber 2004). If the regimen does not contain a PI the change in CD4 count per 3 months is modified (in the base model) by *poorer_cd4rise_fail_nni* (= -6 /mm^3). This applies regardless of viral load level, so PIs are assumed to lead to a more beneficial

CD4 count change than NNRTIs (Ledergerber 2004). We assume in 50% of setting scenarios that this applies also for dolutegravir and in 50% that it does not (determined by *poorer_cd4rise_fail_ii*).

Variability in individual (underlying) CD4 counts for people on ART

Once the mean of the underlying CD4 count is obtained as described above for people on ART, to obtain the CD4 count, variability (*sd_cd4* = 1.2) is added on the square root scale. The estimate was based on unpublished analyses. The ongoing CD4 count increases in people with viral suppression are informed by Mocroft et al 2007. As a result of these assumptions, model outputs for mean changes in CD4 count from start of (efavirenz-based) ART in people with ongoing viral suppression at years 1, 3, 5, 10 were 200, 338, 442 and 633. Amongst people starting ART with CD4 < 100 at start of ART, the proportion of people with a CD4 count above 500/mm3 by 5 years from start of ART was 62%. Similar figures for people with baseline CD4 count 100-200, and 200-300 were 75% and 78% respectively. In comparison, Bishop et al report CD4 count changes of 206, 278, 419, 509 at 1, 3, 5 and 10 years respectively (Bishop 2016). Geng et al report a CD4 count increase of 365 /mm3 in people in southern Africa starting with a CD4 count of 150/mm3 (Geng 2015). At 5 years from start of ART O'Connor et al report a proportion with CD4 count > 500 of 60% for people starting ART with CD4 count < 100 (O'Connor 2014). In the Single trial the CD4 count rise at 1 year was 208/mm^3 and at 3 years was 332/mm^3 (Walmsley 2013, Walmsley 2015).

Each person has a maximum attainable CD4 count given by sampling from exp(Normal(7.3, 0.25^2)).

Table S19. Viral load (mean change from viral load max), CD4 count change (mean change between t-1 and t), and new mutation risk in first 3 months. For 0 active drugs, these are the changes regardless of time from start of ART. For viral load this is the mean of a Normal distribution with standard deviation 0.5 (*sd_v_art*), from which the patient's value/change is sampled. For the CD4 count patients vary in their underlying propensity for CD4 rise on ART (given by sampling from lognormal($1,0.5^2$) and the CD4 count change given here is multiplied by this factor. For the new mutation risk, this is a number that is multiplied by the viral load (mean of values at t-1 and t). The resulting probability is used when assessing whether a new mutation or mutations have arisen.

	Effective adherence between t-1 & t	Number of active drugs												
		3	2.75	2.5	2.25	2.0	1.75	1.5	1.25	1	0.75	0.5	0.25	0
Viral load	≥ 80%	-3.0	-2.6	-2.2	-1.8	-1.5	-1.25	-0.9	-0.8	-0.7	-0.55	-0.4	-0.3	-0.3
(log change	≥ 50%, <80%	-2.0	-1.6	-1.2	-1.1	-0.9	-0.8	-0.6	-0.5	-0.4	-0.25	-0.1	-0.05	-0.1
from vmax)	< 50%	-0.5	-0.4	-0.3	-0.25	-0.2	-0.15	0.0	+0.05	+0.1	+0.1	+0.1	+0.1	0.0
CD4 count	≥ 80%	+50	+45	+40	+35	+30	+25	+20	+17	+13	+10	+5	-2	-15
change	≥ 50%, <80%	+30	+30	+23	+20	+15	+13	+10	+8	+5	+3	0	-7	-17
(t-1 to t)	< 50%	+5	+4	+3	+2	+1	-1	-3	-6	-10	-11	-12	-13	-18
New mutation	≥ 80%	0.002	0.01	0.03	0.05	0.1	0.15	0.2	0.3	0.4	0.45	0.5	0.5	0.5
Risk	≥ 50%, <80%	0.15	0.15	0.2	0.25	0.3	0.3	0.3	0.35	0.4	0.45	0.5	0.5	0.5
(x log viral load)	< 50%*	0.15	0.15	0.2	0.25	0.3	0.3	0.3	0.35	0.4	0.45	0.5	0.5	0.5
	< 50%**	0.05	0.05	0.05	0.05	0.05	0.05	0.05	0.05	0.05	0.05	0.05	0.05	0.05

* for NNRTI-based regimen, ** for boosted PI or integrase inhibitor based regimen.

Table S20. Summary of viral load (mean absolute value or mean change from viral load max) between 3-6 months, and after 6 months if viral load at t-1 > 4 logs. This is the mean of a Normal distribution with standard deviation 0.2, from which the patient's value/change is sampled.

Effective adherence between t-2 & t-1	Effective adherence between t-1 & t	Number of active drugs											
		3	2.75	2.5	2.25	2.0	1.75	1.5	1.25	1	0.75	0.5	0.25
≥ 80%	≥ 80%	0.5	0.8	1.2	1.4	2.0	2.7	-1.7	-1.15	-0.9	-0.75	-0.6	-0.4
≥ 50%, <80%	≥ 80%	1.2	1.2	1.2	1.4	-2.0	-1.6	-1.2	-1.05	-0.9	-0.7	-0.5	-0.35
< 50%	≥ 80%	1.2	1.2	1.2	1.4	-2.0	-1.6	-1.2	-1.0	-0.9	-0.7	-0.5	-0.2
≥ 80%	≥ 50%, <80%	1.2	1.6	1.8	2.2	2.4	-2.4	-1.5	-0.9	-0.7	-0.55	-0.4	-0.3
≥ 50%, <80%	≥ 50%, <80%	2.5	2.5	2.5	2.5	-1.2	-1.1	-0.8	-0.65	-0.5	-0.35	-0.2	-0.05
< 50%	≥ 50%, <80%	-2.0	-1.8	-1.5	-1.35	-1.2	-1.1	-0.8	-0.65	-0.5	-0.2	-0.2	-0.05
≥ 80%	< 50%	-0.5	-0.4	-0.3	-0.25	-0.2	-0.15	-0.10	-0.05	+0.0	+0.0	+0.0	+0.0
≥ 50%, <80%	< 50%	-0.5	-0.4	-0.3	-0.25	-0.2	-0.15	-0.10	-0.05	+0.0	+0.0	+0.0	+0.0
< 50%	< 50%	-0.5	-0.4	-0.3	-0.25	-0.2	-0.15	-0.10	-0.05	+0.0	+0.0	+0.0	+0.0

Table S21. Summary of viral load (mean change from viral load max), CD4 count change (mean change between t-1 and t), and new mutation risk after 6 months, where viral load at t-1 < 4 logs. For viral load this is the mean of a Normal distribution with standard deviation 0.2, from which the patient's value/change is sampled. For the CD4 count patients vary in their underlying propensity for CD4 rise on ART (given by sampling from lognormal(1,0.5^2) and the CD4 count change given here is multiplied by this factor. For the new mutation number, this is a number that is multiplied by the viral load (mean of values at t-1 and t). The resulting probability is used when assessing whether a new mutation or mutations have arisen.

	Effective adherence between t-1 & t	Number of active drugs											
		3	2.75	2.5	2.25	2.0	1.75	1.5	1.25	1	0.75	0.5	0.25
Viral load	≥ 80%	0.5	0.0	1.2	1.6	-2.5	-2.0	-1.4	-1.15	-0.9	-0.75	-0.6	-0.3
(absolute value	≥ 50%, <80%	1.2	1.2	1.2	1.4	-1.2	-1.0	-0.7	-0.6	-0.5	-0.4	-0.3	-0.1
or log change from vmax)	< 50%	-0.5	-0.4	-0.3	-0.25	-0.2	-0.2	-0.1	-0.1	-0.1	-0.1	-0.1	-0.0
CD4 count	≥ 80%	+30	+28	+25	+23	+21	+19	+3	-5	-9	-10.5	-12	-12
Change	≥ 50%, <80%	+15	+13	+10	+8	-4.5	-7.5	-10	-12	-13	-14	-15	-15
(t-1 to t)	< 50%	-13	-14	-15	-15.5	-16	-16.5	-17	-17	-18	-17	-17	-17
New mutation	≥ 80%	0.002	0.01	0.03	0.08	0.10	0.15	0.2	0.3	0.4	0.45	0.5	0.5
risk	≥ 50%, <80%	0.15	0.18	0.2	0.25	0.3	0.3	0.3	0.35	0.4	0.45	0.5	0.5
(x log viral load)	< 50%*	0.15	0.18	0.2	0.25	0.3	0.3	0.3	0.35	0.4	0.45	0.5	0.5
	< 50%**	0.05	0.05	0.05	0.05	0.05	0.05	0.05	0.05	0.05	0.05	0.05	0.05

* for NNRTI-based regimen, ** for boosted PI or integrase inhibitor based regimen.

Viral load and CD4 count changes during ART interruption

Viral load returns to previous maximum viral load (vmax) in 3 months and adopts natural history changes thereafter.

CD4 rate of decline returns to natural history changes (ie those in ART naïve patients) after 9 months, unless the count remains > 200 above the CD4 nadir

Rate of CD4 count decline depends on current viral load. c(t) is the CD4 count at time t, cmin(t) is the CD4 count nadir measured by time t and cc(t-1) is the change in CD4 count from t-1 to t.

if time off ART = 3 months or if time off ART > 3 months and CD4 in previous period is > 300 above the minimum CD4 count to date

v(t) = vmax(t-1)
if v(t) ≥ 5 then cc(t-1) = Normal (-200,10^2)
if 4.5 <= v(t) < 5 then cc(t-1) = Normal (-160,10^2)
if v(t) < 4.5 then cc(t-1) = Normal (-120,10^2)

If this leads to c(t) < cmin(t) (CD4 nadir) then c(t) is set to cmin(t)
if time off ART = 6 months:-

if v(t) ≥ 5 then cc(t-1) = Normal (-100,10^2)
if 4.5 <= v(t) < 5 then cc(t-1) = Normal (-90,10^2)
if v(t) < 4.5 then cc(t-1) = Normal (-80,10^2)

if time off ART = 9 months:-

if v(t) ≥ 5 then cc(t-1) = Normal (-80,10^2)
if 4.5 <= v(t) < 5 then cc(t-1) = Normal (-70,10^2)
if v(t) < 4.5 then cc(t-1) = Normal (-60,10^2)

This is broadly based on evidence from a number of analyses of the effects of ART interruption (e.g. (d'Arminio Monforte 2005, Li 2005, Mocroft 2001, Wit 2005)

Incidence of new current toxicity and continuation of existing toxicity

Toxicities including gastrointestinal symptoms, rash, hepatoxicity, CNS toxicity, lipodystrophy, hypersensitvity reaction, peripheral neuropathy and nephrolithiasis can occur with certain probability on certain specific drugs (Table S22). These probabilities are based broadly on evidence from trials and cohort studies, although there are no common definitions for some conditions which complicates this.

Table S22. Risk of development of specific drug toxicities and probability of switching away from the drug if toxicity occurs. Note toxicity also increase the risk of treatment interruption and affects drug adherence.

Toxicity	Drug	Risk of development per 3 months	Probability of continuation if pre-existing	Probability of switching away from drug per 3 months if toxicity present
Nausea	atazanavir	1% (5-fold higher in 1st year)	50%	5%
	zidovudine	3% (5-fold higher in 1st year)	50%	8%
Diarrhoea	atazanavir	1% (2.5-fold higher in 1st year)	50%	5%
Rash	Efavirenz	3% (in first 6 months on efavirenz)		5%
CNS toxicity	efavirenz	10% (if been on efavirenz <1 year)	80% if been on efavirenz <1 year. 90% if been on efavirenz ≥1 year	2%
	dolutegravir	5% (if been on dolutegravir <1 year)	40% if been on dolutegravir <1 year. 90% if been on dolutegravir ≥1 year	2%
Lipodystrophy	zidovudine	1.5%	100%	1%
Anaemia	zidovudine	3% (1.5-fold higher in 1st year)	20%	5%
Headache	zidovudine	10% (1.5-fold higher in 1st year)	40%	2%
Lactic acidosis	zidovudine	0.02%		100%
Renal dysfunction	tenofovir	0.35%	100%	20%
Weight gain	dolutegravir	1%	100%	0%

Switching of drugs due to toxicity

If toxicity is present then we consider in some scenarios that drugs may be switched due to toxicity.

Emergence of specific resistance mutations and their effect on drug activity

newmut (see Table S19 – S21 above) is a probability used to indicate the level of risk of new mutations arising in a given 3 month period. If this chance comes up in a given 3 month period (determined by sampling from the binomial distribution) then the following criteria operate.

Table S23. Risk of acquiring new resistance mutations.

Resistance mutation	Probability of arising	Conditions
M184	80%	if on 3TC or FTC
# TAMS increases by 1	20%	if on zidovudine and (not on 3TC nor FTC)
	12%	if on zidovudine and (on 3TC or FTC)
# TAMS increases by 2	1%	if on zidovudine and (not on 3TC nor FTC)
	1%	if on zidovudine and (on 3TC or FTC)
K65	10%	if on tenofovir
Q151	2%	if on zidovudine
K103	60%	If on efavirenz
Y181	10%	If on efavirenz
G190	10%	If on efavirenz
I50L	3%	If on atazanavir
I84V	3%	If on atazanavir
N88	3%	If on atazanavir
Integrase codon: 118	*pr_res_dol* *	if on dolutegravir or cabotegravir
140	*pr_res_dol*	if on dolutegravir or cabotegravir
148	*pr_res_dol*	if on dolutegravir or cabotegravir
155	*pr_res_dol*	if on dolutegravir or cabotegravir
263	*pr_res_dol*	if on dolutegravir or cabotegravir

*See Table S27 below of sampled parameters.

These values are chosen, in conjunction with values of *newmut*, to provide estimates of accumulation of specific classes of mutation consistent with those observed in clinical practice (Cozzi-Lepri 2010, Phillips 2001, Sigaloff 2012) They reflect a greater propensity for some mutations to arise than others. This probably relates to the ability of the virus to replicate without the mutations (e.g. probably very low in the presence of 3TC for virus without M184V) as well as the replicative capacity of virus with the mutations.

New resistance to NNRTI arising as a result of ART interruption

It is assumed that due to the long half life of NNRTIs nevirapine and efavirenz, stopping of a regimen containing one of these drugs is associated with a specific probability of an NNRTI resistance mutation arising (see, for example, Fox et al, 2008. The respective probabilities for K103, Y181 and G190 are 1.8%, 0.06% and 0.6%.

Loss of acquired mutations from majority virus

It is assumed that mutations tend to be lost from majority virus with a certain probability from 3 months after stopping to take a drug that selects for that mutation. The probability of losing mutations per 3 months (from 3 months after stopping) is as follows (Deeks 2003, Devereux 1999, Hance 2001, Tarwater 2003, Walter 2002).

Table S24. Probability of loss of acquired mutations from majority virus per 3 months after stopping drugs selecting for mutation.

M184V	0.8
L74V	0.6
Q151M	0.6
K65R	0.6
TAMS*	0.4
NNRTI mutations	0.05
Protease mutations	0.2
Integrase mutations	0.2

* to lose all TAMS

Mutations are regained in majority virus if a drug selecting for the mutation is again started.

Determination of level of resistance to each drug

Table S25. shows the level of resistance to each drug according to presence of specific resistance mutations.

Table S25. Level of resistance to each drug according to presence of specific resistance mutations.

**Resistance mutation**	**Drug**	**Level of resistance (1=full resistance)**	**Condition**
M184	3TC or FTC	0.75	
1-2 TAMS	zidovudine	0.5	No 3TC or FTC in regimen
	zidovudine	0.25	3TC or FTC in regimen and ever had M184V
	zidovudine	0.5	3TC or FTC in regimen and never had M184V

2-3 TAMS	tenofovir	0.5	
3-4 TAMS	zidovudine	0.75	No 3TC or FTC in regimen
	zidovudine	0.5	3TC or FTC in regimen and ever had M184V
	zidovudine	0.75	3TC or FTC in regimen and never had M184V
4 or more TAMS	tenofovir	0.75	No 3TC or FTC in regimen, or 3TC in the regimen and never had M184V
	tenofovir	0.5	3TC or FTC in regimen and ever had M184V
5 or more TAMS	zidovudine	1.0	No 3TC or FTC in regimen
	zidovudine	0.75	3TC or FTC in regimen and ever had M184V
	zidovudine	0.75	3TC or FTC in regimen and never had M184V
Q151	3TC or FTC	0.25	
	zidovudine	0.75	
K65	3TC or FTC	0.25	
	tenofovir	0.75	
K103	efavirenz	1.0	
Y181	efavirenz	0.75	
G190	efavirenz	0.75	
I501	atazanavir	1.0	
N88	atazanavir	1.0	
I84	atazanavir	1.0	
1 - 3 of (V32, M46, I54, V82, L90)	atazanavir	0.5	
At least 4 of (V32, M46, I54, V82, L90)	atazanavir	1.0	
Integrase mutation codon 118	Dolutegravir or cabotegravir	0.75 or 1.00 *	
Integrase mutation codon 140	Dolutegravir or cabotegravir	0.75 or 1.00 *	
Integrase mutation codon 148	Dolutegravir or cabotegravir	0.75 or 1.00 *	
Integrase mutation codon 155	Dolutegravir or cabotegravir	0.75 or 1.00 *	
Integrase mutation codon 155	Dolutegravir or cabotegravir	0.75 or 1.00 *	

* Determined by *res_level_dol_cab_mut*

These rules approximately follow the interpretation systems for conversion of mutations present on genotypic resistance test into a predicted level of drug activity (or, equivalently, of resistance; http://hivdb.stanford.edu, http://www.hivfrenchresistance.org/

Calculation of activity level of each drug

For drugs with a potency of 1 the activity level is 1-level of resistance. For ritonavir boosted PIs, which are asigned a potency of 2 it is given by 2 – (2 x level of resistance). Potency is assumed higher due to the ability to induce sustained viral suppression alone. Activity levels of each drug in the regimen are summed to give the total number of active drugs. For dolutegravir the potency is assumed to be 1.5 (the modal value of the distribution) so the activity is 1.5 – (1.5 x level of resistance). We also consider a range of values for the potency of dolutegravir, as described below (and determined by *dol_higher_potency*).

Occurrence of clinical disease and death in HIV infected people

The basic underlying rate of clinical diseases according to CD4 count, viral load and age per 3 months is given in Table S26 below. We explain further below how these rates are modified to obtain separate CD4 count- and viral load-specific rates for TB, other WHO stage 3 events, serious bacterial infections, crypococcal meningitis, and other WHO stage 4 conditions.

Table S26. Basic underlying rate of clinical disease according to CD4 count, viral load, age, and being on ART.

**Effect of CD4 count**			
If cd4 ≥ 650	rate=0.002	if 500 ≤ cd4 < 650	rate=0.010
if 450 ≤ cd4 < 500	rate=0.013	if 400 ≤ cd4 < 450	rate=0.016
if 375 ≤ cd4 < 400	rate=0.020	if 350 ≤ cd4 < 375	rate=0.022
if 325 ≤ cd4 < 350	rate=0.025	if 300 ≤ cd4 < 325	rate=0.030
if 275 ≤ cd4 < 300	rate=0.037	if 250 ≤ cd4 < 275	rate=0.045
if 225 ≤ cd4 < 250	rate=0.055	if 200 ≤ cd4 < 225	rate=0.065
if 175 ≤ cd4 < 200	rate=0.080	if 150 ≤ cd4 < 175	rate=0.10
if 125 ≤ cd4 < 150	rate=0.13	if 100 ≤ cd4 < 125	rate=0.17
if 90 ≤ cd4 < 100	rate=0.20	if 80 ≤ cd4 < 90	rate=0.23
if 70 ≤ cd4 < 80	rate=0.28	if 60 ≤ cd4 < 70	rate=0.32
if 50 ≤ cd4 < 60	rate=0.40	if 40 ≤ cd4 < 50	rate=0.50
if 30 ≤ cd4 < 40	rate=0.80	if 20 ≤ cd4 < 30	rate=1.10
if 10 ≤ cd4 < 20	rate=1.80	if 0 ≤ cd4 < 10	rate=2.50
**Independent effect of viral load**			
if v < 3	rate = rate x 0.2		
if 3 <= v < 4	rate = rate x 0.3		
if 4 <= v < 4.5	rate = rate x 0.6		
if 4.5 <= v < 5	rate = rate x 0.9		

if 5 <= v < 5.5	rate = rate x 1.2
if 5.5 <= v	rate = rate x 1.6

Independent effect of age

rate = rate x $(age / 38)^{1.2}$

Independent effect of being on ART

For patients on ART (a 3 drug regimen) the rate is multiplied by 0.6 (*ind_effect_art_hiv_disease_death* = 0.6), to reflect that being on ART has a positive effect on risk of HIV-related clinical disease and death independent of latest CD4 count and viral load.

This is informed by Phillips et al 2004.

Occurrence of WHO stage 3 diseases

WHO stage 3 conditions are modelled as a group, with the exception of TB being modelled separately. Risk of a WHO stage 3 disease event in a given 3 month period is given by the above basic underlying rate of clinical disease multiplied by a relative rate *fold_incr_who3* (which currently takes the value 5). The rate for non-TB WHO stage 3 disease is 4/5 of the resulting rate. The rate for TB is 1/5 of the resulting rate (and hence effectively the rate of TB is the same as the rate of all WHO stage 4 conditions combined).

Occurrence of active TB

As mentioned above, the rate of TB is equal to the overall underlying rate of WHO stage 4 disease.

TB preventative treatment (TPT) is recommended by WHO for people with HIV without identified active TB. The rate of use of TPT is determined by *rate_tb_proph_init*. This has recently been coded and currently the value for this in the Core model is 0. Effects of application of policies involving TPT are being explored in specific analyses and we anticipate explicitly including use of TPT in future updates to the Core model program. The assumed effect of TPT on risk of active TB is *effect_tb_proph* currently taking the value 0.5 for a duration of 1 year.

On development of active TB, we assign whether this is diagnosed “early” or “late”, with the implication of a higher probability of survival if diagnosed early. The base probability of being diagnosed late is given by *tb_base_prob_diag_l* (sampled from a distribution of 0.25, 0.50, 0.75, with equal probability). People that are under care for their HIV have a greater chance that, if they develop active TB, this is diagnosed early (*effect_visit_prob_diag_l*, 0.50 0.67 0.80). In some policies investigated there is assumed to be TB LAM testing available. If TB LAM testing is available then this has the effect of increasing the probability of active TB being diagnosed early

(*tblam_eff_prob_diag_l* = 0.5). We do not explicitly model use of specific TB drugs – we assume a reduction in risk of death in those diagnosed early due to treatment (see below).

Occurrence of cryptococcal meningitis

The risk of cryptococcal meningitis is given by the above-described underlying rate of clinical disease multiplied by a parameter representing the proportion of WHO stage 4 conditions that are CM (*prop_adc_crypm* - current value 0.15).

As with TB, we assign whether this is diagnosed "early" or "late", with the implication of a higher probability of survival if diagnosed early. The base probability of being diagnosed late is given by *crypm_base_prob_diag_l* (sampled from a distribution of 0.25, 0.50, 0.75, with equal probability). People that are under care for their HIV have a greater chance that, if they develop cryptococcal meningitis, this is diagnosed early. This effect is assumed the same as for TB and other diseases for which we model early/late diagnosis (*effect_visit_prob_diag_l*). We consider some strategies in which cryptococcal antigen testing is employed, with its effect being to increase the probability of cryptococcal meningitis being diagnosed early rather than late (*crag_eff_prob_diag_l* current value = 0.5). if *crag_cd4_l200* or *crag_cd4_l100* then if the CD4 count at presentation or return to care is measured and it is below 200 / 100 then plasma crag testing is done.

In some strategies we consider use of prophylaxis to prevent cryptococcal meningitis (with low dose fluconazole). The rate of use of this is given by *rate_crypm_proph_init*, currently = 0. Fluconazole prophylaxis is assumed to result in a decline in risk of cryptococcal meningitis given by *effect_crypm_proph*, currently with the value = 0.5 for a period of one year. We do not explicitly model use of specific drugs to treat cryptococcal meningitis – we assume a reduction in risk of death in those diagnosed early due to treatment (see below).

Occurrence of serious bacterial infection (SBI)

The risk of SBI is given by the above-described underlying rate of clinical disease multiplied by a parameter representing the proportion of WHO stage 4 conditions that are a SBI (*prop_adc_sbi* -current value 0.15). We assign whether this is diagnosed "early" or "late", with the implication of a higher probability of survival if diagnosed early. The base probability of being diagnosed late is given by *sbi_base_prob_diag_l* (sampled from a distribution of 0.25, 0.50, 0.75, with equal probability). People that are under care for their HIV have a greater chance that, if they develop SBI, this is diagnosed early. This effect is assumed the same as for other diseases for which we model early/late diagnosis (*effect_visit_prob_diag_l*).

Occurrence of other WHO stage 4 diseases

The risk of occurrence of an other (non-cryptococcal meningitis, non-SBI) WHO stage 4 condition is given by the underlying base rate of clinical disease multiplied by (1 -

prop_adc_sbi - prop_adc_crypm). We assign whether this is diagnosed "early" or "late", with the implication of a higher probability of survival if diagnosed early. The base probability of being diagnosed late is given by *oth_adc_base_prob_diag_l* (sampled from a distribution of 0.25, 0.50, 0.75, with equal probability). As for all WHO stage 4 conditions, people that are under care for their HIV have a greater chance that, if they develop the disease, this is diagnosed early.

Use and effect of co-trimoxazole

From 2015 onwards, there is an 80% chance that people under care for their HIV are on co-trimoxazole prophylaxis. This has the effect of reducing the underlying base risk of clinical disease by a multiple 0.8 (*effect_pcp_p_death_rate* = 0.8).

Measurement of CD4 count

We have a parameter which determines whether the policy is for CD4 count to be measured each time a person comes back into care or have viral load > 1000 cps/mL, to see if have advanced HIV disease (as in WHO guidelines). The above-described approaches to enable early diagnosis of clinical disease tend to rely on CD4 measurement to identify those with advanced HIV disease.

Risk of HIV-related death

The underlying risk of HIV-related death is given by the underlying base rate of clinical disease, multiplied by a factor *fold_decr_hivdeath* (current value = 0.25 [0.15 Zimbabwe]). Beyond this there is a multiplicative increase in risk of death in a 3-month period in which TB or a WHO stage 4 condition occurs; *incr_death_rate_tb, incr_death_rate_oth_adc, incr_death_rate_crypm, incr_death_rate_sbi*.

The effect of TB being diagnosed early on rate of death from the tb event is given by *rel_rate_death_tb_diag_e*, the value of which is sampled from the values 0.50 0.67 0.80. Similarly for cryptococcal meningitis, SBI, and other WHO stage 4 disease (*rel_rate_death_oth_adc_diag_e, rel_rate_death_crypm_diag_e, rel_rate_death_sbi_diag_e*).

Some deaths are related to CD4 but will not go down as who4_ related (e.g. other cancers, but not including liver death) so a proportion (15%) are classified as non-HIV deaths.

CVD death risk

Risk of death from CVD depends on systolic blood pressure, age and gender.

cvd_death_risk = *base_cvd_death_risk* * exp (((age - 15) * *effect_age_cvd_death*) + (*effect_gender_cvd_death**(gender - 1)) + ((sbp - 115)* *effect_sbp_cvd_death*)) ;

where *base_cvd_death_risk* = 0.00002 *effect_age_cvd_death* = 0.03 *effect_gender_cvd_death* = 0.4 and *effect_sbp_cvd_death* = 0.05

This applies also to HIV negative people.

Other non-HIV-related death in people with HIV

Risk of non-HIV death is based on age specific death rates from South Africa in 1997, before the main impact of HIV. South Africa has a death registration system and hence provides a reliable setting from which to source death rates. These are given in Table S2.

Since CVD is modelled separately, we modify these rates to remove the CVD component. Based on data suggesting 10% of deaths in over 50's and 35 in 15-49 year olds are due to CVD (Sifuna et al 2018). Thus we multiply the rates applied from Table 2 by 0.93 or 0.90 depending on age.

There is an additional risk of death of 0.005 during a period in which there is presence of a nephrologic toxicity, and of 0.10 in a period in which there is presence of the drug toxicity of lactic acidosis.

Risk of non-HIV death is increased by a factor rr_non_aids_death_hiv_off_art in people with HIV who are not on ART (due to effects of HIV on non-AIDS conditions) and rr_non_aids_death_hiv_on_art for people on ART.

In addition, there is an age-specific risk of death due to COVID-19 during the period 2020.25 to 2021.75.

Death in HIV negative people

The death rate in HIV negative people is as for non-HIV death in people with HIV, with the exception that we explicitly model TB death in HIV negative people.

Risk of TB is given by the parameter *non_hiv_tb_risk* (current value = 0.0005). We again model early and late diagnosis and assume a reduced death risk in those diagnosed early. Death risk is given by *non_hiv_tb_death_risk* (current value = 0.3).

Local community TLD PEP/PrEP/ART Access

For some policy questions, we consider the possibility of introduction of community access to tenofovir-lamivudine-dolutegravir, without prescription, along with free self-test kits (referred to as "community TLD"). The primary intent would be that post-exposure prophylaxis (PEP) is readily available to people who have had risky condomless sex, within at most 24 hours if possible. Giving unrestricted access to TLD would mean some people might use the drugs as pre-exposure prophylaxis (PrEP) or as treatment without formally engaging with the health system. Therefore the net effects on health outcomes are uncertain. Potential beneficial effects of making TLD widely available locally (hereafter referred to as community TLD) include: (i) higher PEP/PrEP prevention coverage for episodes of condomless sex in HIV negative people (ii) greater likelihood of people living with HIV being on ART, and (iii) lower risk of resistance in any person taking TLD as PrEP rather than TDF/FTC. Potential negative effects include: (i) lack of initial / 3- monthly HIV testing in some PrEP and PEP users, meaning some have undiagnosed HIV and hence are taking TLD guided by sexual risk when it should be continuous, with a possible increased risk of resistance, (ii) possible lower uptake of long-acting injectable cabotegravir due to easier access of TLD PEP/PrEP (iii) an increase in the proportion of people on ART who are not under care and not monitored with viral load testing or given adherence counselling, possibly leading to lower ART effectiveness; (iv) increased risk of immune response inflammatory Syndrome (IRIS) in people with advanced HIV and other concurrent infections starting ART without clinical assessment, and (v) some use of TLD in people without HIV and without risk for HIV which results in some drug wastage and possibly unnecessary drug toxicity.

Modelling of PrEP was described in section 7 and, since PrEP is assumed to be risk-informed and used when there is an "indication" for PrEP in a 3-month period; i.e. if person has condomless sex with at least one non-primary partner, or if the primary partner may have unsuppressed HIV (see section 7 for full detail).
Some people are considered to live in circumstances that makes them unable to access clinic services and these will not access HIV testing or regular clinic-based PrEP (variable hard_reach = 1). With community TLD introduction, for all people with an indication for PrEP/PEP there is a possibility of TLD being used, as PEP/PrEP, with those unable to access clinic services assumed to have access due to local free availability.
In relation to the higher PEP/PrEP prevention coverage during periods of condomless sex, we introduce a parameter *prob_prep_pop_wide_tld*, which is the probability of starting TLD PEP / PrEP in a given 3 -month period for a person who is PEP / PrEP willing (see section 7) and has an indication for PEP / PrEP when community TLD access is available. *prob_prep_pop_wide_tld* is sampled from the following distribution: 0.05: 50%; 0.1: 50%;

Under local community TLD, the proportion of PEP/PrEP use in people with a PEP/PrEP indication that is as PEP rather than PrEP is represented by *prop_pep* (0.5: 33% 0.7: 33% 0.9: 33%). The efficacy of PEP for HIV prevention is indicated by the parameter *pep_efficacy* (0.9: 80% 0.95: 20%) (Irvine et al), while, as described in section 7, the efficacy of oral PrEP (*prep_oral_efficacy*) is (0.9: 20% 0.95: 80%). The effectiveness of PEP is, like oral PrEP, also dependent on adherence. We also consider that self-taking TLD as PEP/PrEP as a result of local community availability but without clinical

supervision could decrease adherence or could increase adherence since people have self-taken the decision to use PEP/PrEP (indicated by the parameter *pop_wide_prep_adh_effect (no change: 60% 0.75-times:10%, 0.9 times:10%, 1/0.75 times: 10%, 1/0.9: times: 10%*).
Regarding the lack of initial / 3- monthly HIV testing in people with a PrEP indication, some people who are taking PEP/PrEP due to community access may nevertheless be tested each three months under clinical supervision. *prob_test_pop_wide_tld_prep* (0.1: 33% 0.25: 33% 0.5: 33%) gives the probability per three months of this occurring.

Since there is easier local access to drug without the need for clinic attendance, which can mean unaffordable cost in time or travel cost, there is assumed to be a lower rate of interruption of ART given by *rr_interrupt_pop_wide_*tld (1/1.5: 30% 1/2: 30% 1/3: 30%   1/5: 10%), which gives the degree to which the rate of interruption of ART is reduced with local community TLD access, while *rr_return_pop_wide_tld* (1.5: 25% 2:25%   3:25%   5: 25%) gives the degree to which the rate of restarting ART in people who had discontinued is increased with local community TLD access.

Regarding the increase in the proportion of people on ART who are not under care and not monitored *prob_onartvis0_0_to_1* (0.02: 33% 0.05: 33% 0.1: 33%) and prob_onartvis0_1_to_0 (0.005: 25%   0.01: 25%   0.03: 25%   0.05: 25%) are the probabilities that a person who is on ART under clinical care will transition to self-taking ART (and the reverse). For people on ART but not visiting clinic, we assume they are not monitored or receiving possible benefits of enhanced adherence counselling, nor are they receiving benefits of prevention and early diagnosis associated with the WHO advanced HIV disease package that is assumed available if required to 80% of people visiting clinic . This is implemented as a 25% higher death rate.

A possible additional effect on adherence beyond this of not being under clinical monitoring (onartvisit0=1) is determined by *artvis0_lower_adh* (0 no effect: 80% 1, lower adherence: 20%).   If *artvis0_lower_adh* = 1 then the adherence is assumed to be lower in 50% of people with artvisit01 and the extent of the lower adherence per 3-month period per person is given by sampling from a beta(1.5, 10) distribution (resulting in a mean lower adherence by 0.13).

People that have never tested for HIV in a clinical context may use TLD if they believe they have HIV, possibly based on use of a self-test kit available in local communities with TLD. There could be some people who are HIV negative who start TLD due to a false positive self-test or due to thinking they have HIV without testing. The parameter *pop_wide_tld_selective_hiv* (10:50% 50:50%) indicates how many times greater the probability of a person with HIV starts TLD without a clinic-confirmed positive HIV test compared with a person without HIV.   A parameter *prob_tld_hiv_concern* (0: 33% 0.001:33% 0.005:33%) represents the probability per 3 months of a person who has never tested for HIV but has had at least one short-term condomless partner in the past starting TLD. This is for a person with HIV. The probability is *pop_wide_tld_selective_hiv* times lower for a person without HIV.

Additional absolute risk of IRIS when starting ART with CD4 count < 100 and not under clinical supervision is given by *death_r_iris_pop_wide_tld* (0.01: 33% 0.03: 33% 0.05: 33%) (Sereti et al).

Disadvantages in accessibility to care for sex workers and effect of a sex worker program

We consider that sex workers have disadvantages in terms of lower ability to engage in care (determined by *sw_art_disadv*). These disadvantages can be in terms of a higher probability of not engaging in care after HIV diagnosis (*sw_higher_prob_loss_at_diag*), higher rate of interruption of ART (*sw_higher_int)* and lower tendency to be able to adhere to ART (e.g. due to mobility) (*rel_sw_lower_adh*).
We also consider that a Key Populations (KP) program exists to support sex workers (determined by *sw_program*) since 2010. The rate of engagement (*rate_engage_sw_progra*m) is (0.03: 33% 0.05: 33% 0.10: 33%/per 3 months) and the rate of disengagement *(rate_disengage_sw_progr*am) is (0.01: 50% 0.03: 50%). There are potential effects of such a program, the magnitudes of which are determined by sampling. We consider effects of a sex worker program on: levels of condomless sex, whether 6 monthly HIV testing is done, , the propensity to take PrEP, and the persistence on bacterial any STI they may acquire. Such a program will also impact on the disadvantages a sex worker has; it will lower the rate of interruption of ART for women on ART, increase the level of adherence to ART for women on ART and increase the probability of engaging with care for women at time of HIV diagnosis. Additional effects of the AMETHIST intervention are shown on page 3.

Distributions for parameters

In Table S27 below, we describe all the parameters from which we sample for each model run to create setting scenarios. Some parameters are overwritten with new values to generate epidemics specific to Zimbabwe, these are labelled ‘Zim’.

Table S27. Parameter distributions sampled for each model run / setting scenario ^ Parameters are sampled for each model run as indicated. Of model runs, those with HIV incidence in 15-49 year olds below 0.2/100 person years or HIV prevalence in 15-49 year olds below 30% in mid-2022 were excluded. See also Supplementary Table 1 of Supplementary Tables which compares epidemic characteristics of setting-scenarios with observed data.

**Parameter name^**	**Description**	**Distribution sampled (value; % with value)**	**Motivation for distribution**
Population demographics			
inc_cat	Three future demographic structures with differing levels of population growth.	1: 33% 2: 33% 3: 33%	Different countries in sub-Saharan Africa have different population growth rates so we consider a range from around 1% to 3% per year (UN WPP)
Parameters relating to sexual behaviour			
base_rate_sw	Base rate per 3 months of a woman becoming a sex worker (also influenced by age and lifetime propensity)	0.0010: 50% 0.0015: 50%	Informed by data on the proportion of women who are sex workers (Vandepitte 2006) and by Population Size Estimates (Cowan 2023)
base_rate_stop_sexwork	Base rate per 3 months of a sex worker stopping sex work (also influenced by age)	0.005: 40% 0.01:20% 0.015: 40%<br><br>Zim: 0.005: 40% 0.01:40% 0.015: 20%	Informed by data on the proportion of women who are sex workers and duration of sex work (Vandepitte 2006) by Population Size Estimates (Cowan 2023)
sw_trans_matrix	Transition matrices determining probabilities of transition between categories of number of condomless partners had in the 3 month period by sex workers. (See Table S9 above)	1: 50% 2: 20% 3: 30%	We consider a range of matrices to reflect uncertainty. Reports of condomless partner numbers are associated with inaccuracy of recall and potential bias in over-estimating consistency of condom use. (Cowan 2017)
p_rred_sw_newp	Effect of population level changes in sexual behaviour on the probability of starting and stopping sex work and/or moving to a lower category of number of condonless sex partners (see section 3 above)	0.01: 33% 0.03: 33% 0.10: 33%	Population level changes in sexual behaviour are believed to partially explain changes in HIV incidence in early phases of the HIV epidemic. (Gregson 2010, Halperin 2011)
newp_factor	To express uncertainty in underlying propensity of whole population to form short-term partnership with condomless sex,	0.5:33% 1.0:33% 2:33%	

**Parameter name^**	**Description**	**Distribution sampled (value; % with value)**	**Motivation for distribution**
sex_beh_trans_matrix_m and sex_beh_trans_matrix_m	Matrix determining rate of transition between four levels of sexual behaviour. There are 15 versions for each of men and women.	1/15 probability for each transition matrix for men, the same for women	Due to the fact that data on sexual behaviour are from self-report, which is known to be highly unreliable (Yeatman 2011, Gregson 2002, Desmond 2018, Glynn 2011), there is uncertainty over longitudinal patterns of sexual behaviour and the degree of skewness in the distribution of number of new partners we consider a range of possible matrices (15 for each gender = 225 possible combinations).<br><br>Jointly with other parameters in this section these help to determine the extent to which risk behaviour is concentrated both between individuals and within individuals over time (Mishra 2015)
sex_age_mixing_matrix_m and sex_age_mixing_matrix_w	Matrix determining the age gender sexual mixing from male and female perspectives	6 different matrices for each gender persective, sampled at random	Uncertainty about mixing patterns by age and gender so we consider a range of these.
p_rred_p	Indicates the proportion of the population in whom the sexual risk behaviour is very low	0.3: 33% 0.5: 33% 0.7: 33%	In order to include a person-level effect on sexual behaviour this and the parameter below allow the population to be divided into three according to the lifelong tendency to have short term condomless sex partners.
p_hsb_p	Indicates the proportion of the population in whom the sexual risk behaviour has a tendency to be higher than average	0.05: 33% 0.08: 33% 0.15: 33%	As above
rred_a_p	Relative condomless sex levels by gender and age; four different patterns.	1: 15% 2: 15% 3: 35% 4: 35%	Uncertainty over levels of condomless sex by age so we consider a range of possibilities
eprate	Base rate (youngest age group) of starting to have a long term condomless sex partner.	Lognormal(0.1, 0.25)	Informed by outputs that give the proportion of people with a long term condomless sex partner by age.
conc_ep	Parameter indicating the degree to which those with a primary condomless sex partner have a lower of higher probability of short term (non-primary) condomless sex partners than those without a primary condomless sex partner.	0.333: 33% 1: 33% 3: 33%	This is likely to vary across setting scenarios and we wished to consider across the range. Again, this distribution of values was found, in certain (randomly selected) combination with other sexual behaviour parameter values to re-produce

Parameter name^	Description	Distribution sampled (value; % with value)	Motivation for distribution
			epidemics within the observed prevalence range.
ych_risk_beh_newp	Degree of reduction in condomless sex with short term partners per year from 1995 – 2000	0.5: 5% 0.6: 40% 0.70: 30% 0.80:15% 0.9: 5% 1.0:5%	In order to explain the decrease in incidence and prevalence of HIV in southern Africa in the late 1990s it is necessary to assume there was a reduction in condomless sex, which is supported by data in Zimbabwe (Gregson 2002, Gregson 2010, Halperin 2011)
ych_risk_beh_ep	Degree of reduction in condomless sex per year with long term partners from 1995-2000	0.8: 25% 0.9: 25% 0.95: 25% 1.0: 25%	As above
ch_risk_diag_newp	Degree of reduction (fold change) in condomless sex with short term partners in a person diagnosed with HIV	0.7: 25% 0.8: 25% 0.9: 25% 1.0: 25%	Informed by (Fonner 2012)
ch_risk_diag	Degree of reduction in condomless sex with long term partner in a person diagnosed with HIV	0.7: 25% 0.8: 25% 0.9: 25% 1.00: 25%	As above
ych2_risk_beh_newp	Degree of change in condomless sex with short term partners per year from 2010 – 2015	1/0.975: 5% 1/0.990: 5% 1/0.995:15% 1:50% 0.995:15% 0.990:5% 0.975: 5%	It is uncertain whether there have been recent changes in condomless sex, hence a neutral distribution was used.
exp_setting_lower_p_vl1000<br><br>*external_exp_factor*<br>*rate_exp_set_lower_p_vl1000*	Whether there is exposure of individuals to settings with lower population viral suppression levels, due to migration (and return)<br>Measure of level of effect of the above exposure<br>Rate of exposure	In 20% of runs<br><br>Uniform(1,2)<br>Uniform(0, 0.01)	A subset of a population may have sexual exposure to others outside the population and the degree of this will be setting dependent so this is varied across model runs.
higher_newp_with_lower_adhav	In people with lower adherence to ART there is a tendency for lower number of condomless partners	In 20% of runs	There could be correlation between ART adherence and sexual risk behaviour, in either direction
**Parameters relating to male circumcision**			
circ_inc_rate<br><br><br>*rel_incr_circ_post_2013*	Determines the rate with which male circumcision increases over time<br><br><br>Relative increase in VMMC after 2013	0.0001: 10% 0001: 30% 0.003: 40% 0.01: 10% 0.10: 10%<br><br>Zim: 0.0001: 25% 0.01: 50% 0.02: 25%<br><br>0.8: 10% 1: 25% 3: 25% 7: 40%	This varies by country in the region.<br><br><br>Due to for example PEPFAR programmes

Parameter name^	Description	Distribution sampled (value; % with value)	Motivation for distribution
rel_incr_circ_post_2023	Relative increase in VMMC after 2023	Zim: 2: 25% 3: 50% 4: 25%<br><br>Zim: 2: 25% 3: 50% 4: 25%	
circ_inc_15_19<br>*circ_red_20_30*<br>*circ_red_30_50*	Relative increases in age groups	1.5: 33% 2.0:33% 3.0: 33%<br>0.3: 33% 0.4:33% 0.5: 33%<br>0.15: 33% 0.25:33% 0.35: 33%<br><br>0.05: 33% 0.1: 32% 0.4: 20% 0.9: 10%	
prob_birth_circ	Probability of circumcision at birth	Zim: 0.05: 25% 0.1: 50% 0.15: 25%	
**Parameters relating to being hard to reach for services**			
*p_hard_reach_w_*	Proportion of women that have a propensity to be hard to reach with prevention and testing servcices	Uniform(0.05, 0.15)	A small proportion of people have a long term propensity not to take up HIV services, for various possible reasons including stigma, physical barriers, etc. (Grimsrud 2020)
hard_reach_higher_in_men	The extent to which this is higher in men (in men this also includes propensity to be medically circumcised)	Uniform(0,0.1)	
**Parameters relating to HIV testing**			
an_lin_incr_test	Parameter determining the rate of increase in HIV testing (any testing outside ANC)	0.0001: 20% 0.0005: 25% 0.003: 35% 0.01: 10% 0.02: 5% 0.03: 5%<br><br>Zim: 0.0001: 35% 0.0005: 30% 0.003: 20% 0.01: 15%	Range and pattern required to re-produce the observed range in proportion of HIV positive people diagnosed (see Table 1 of main paper).
*date_test_rate_plateau_*	Year in which the rate of HIV testing plateaus.	2011: 10% 2013: 10% 2015: 20% 2017: 30% 2019: 30%<br><br>Zim: 2019	Some countries have increased testing rates markedly and these have plateaued at different levels in different settings (e.g Malawi MoH)
fold_rate_decr_test_future	Parameter that multiplied with *an_lin_incr_test* determines the rate of decrease in HIV testing (any testing outside ANC), from mid-2022	0.25: 33% 0.33: 33% 0.5: 33%<br><br>Zim: 0.25: 25% 0.33: 25% 0.5: 25% 0.75: 25%	

Parameter name^	Description	Distribution sampled (value; % with value)	Motivation for distribution
rate_testanc_inc	Rate of increase in testing in ANC clinics	0.005: 20% 0.01:20% 0.03:20% 0.05:20% 0.1:20%<br><br>Zim: 0.025: 33% 0.03: 33% 0.035:33%	Distribution is intended to reflect variation across settings.
*incr_test_rate_sympt_*	The rate of increase per 3 months in the probability of a person with a WHO stage 3 or 4 disease is tested for HIV.	1.05: 20% 1.10: 20% 1.15: 20% 1.20: 20% 1.25: 20%	Little direct data on this parameter and wide range taken to reflect uncertainty and variation across settings.
max_freq_testing	A parameter defining the maximum frequency with which a person (non sex-worker) without AIDS or WHO stage 3 disease can test for HIV	Annually: 80% 6-monthly: 20%	Policy on frequency of testing varies by setting.
rr_testing_female	Extent to which women have a greater tendency to test for HIV than men. This is beyond the effect of testing in ANC.	1.5: 100%	Implied by higher levels of diagnosis in women compared with men.
test_targeting	Parameter conveying the degree to which HIV testing is targetted towards people having condomless sex since last test.	1: 20% 1.25: 60% 1.5: 20%<br><br>Zim: 1: 70% 1.1: 20% 1.3: 10%	Data on condomless sex since last test not collected but likely to be a higher tendency to test if had sexual risks. We vary the degree of such "targeting". Partially informed by outputs on testing yield.
sens_primary_testtype3	Sensitivity of rapid HIV antibody-only tests for a person in the 3 month period of primary HIV infection	50% 50%: 75%: 50%	(Taylor 2014)
sens_vct	Sensitivity of 3rd generation antibody tests from period after primary infection, for people not on cab-la.	0.98: 100%	
**Parameters relating to PrEP**			
prep_any_strategy	Assumption about people who will choose to take PrEP and when. We assume this will be risk-informed.	Primary assumption (100%): 3 month time periods in which they have condomless sex with at least one short-term partner (*newp*), when they have a long term partner (ep) who is known to have HIV but is not on ART, or when a woman feels there is a high risk her long term partner is in this position (which is implemented as women aged under 50 without HIV and with a long term condomless sex partner who is not on ART having a 5% chance that she will be considered as fulfilling the criteria for *risk-informed* PrEP,	Depends on country strategy. Some evidence that people do tend to appropriately concentrate their use of PrEP to periods of sexual risk (Koss 2020, 2021, Donnell 2021)

Parameter name^	Description	Distribution sampled (value; % with value)	Motivation for distribution
		which becomes 50% if her partner has HIV, based on the assumption that for women who suspect they are at risk are indeed at substantially higher risk that their partner has HIV)<br><br>Alternative assumptions considered separately: as above but restricting by age 15-25, women, sex workers, and with 10% replacing the 5% above.	
low_prep_inj_uptake	Whether there will be substantial uptake of cabotegravir injectable PrEP.	Yes: 67% No: 33%	Uncertainty mainly due to cost.
pref_prep_oral_beta_s1<br><br>*pref_prep_inj_beta_s1*	Whether a person is willing to take oral PrEP (variable prep_oral_willing, prep_inj_willing) depends on the value of the variable pref_prep_oral (pref_prep_inj) which is sampled for each individual from a distribution beta(*pref_prep_oral_beta_s1*, 5), where *pref_prep_oral_beta_s1* currently = 2. *pref_prep_inj_beta_s1 currently = 5 (*ie a higher preference for inj).	2: 100%<br><br>Zim: 0.6:16.7% 0.7: 16.7% 0.8: 16.7% 0.9: 16.7% 1.0: 16.7% 1.1: 16.7%<br><br>2: 20% 3: 20% 4: 20% 5: 20% 6: 20%	We aim for a value which in the context of other parameters determining PrEP use, including similar parameters for cab-la PrEP (inj), which lead to plausible proportions favouring prep_inj over prep_oral and vice versa. These parameter values will be adapted with more experience of use and depending on the exact policy question.
prep_willingness_threshold	If pref_prep_oral is above a lower threshold (*prep_willingness_threshold*, currently = 0.2) then prep_oral_willing = 1, otherwise it is 0. Similarly for pref_prep_inj (we use the same threshold for all types of PrEP).	0.2: 100%	As above, along with other parameters this determines the level of use of oral and cab-la PrEP (cab-la PrEP is referred to by “inj” in parameter and variable names).
rate_test_startprep_any	Additional rate of being tested for HIV (because of interest in PrEP) in people who have never been on PrEP but are eligible for it. Represents the probability he or she can access it easily enough to mean that they do indeed start. Referring to “all” in variable and parameter names means any type of PrEP without distinguishing between oral and cab-la.	0.1: 50% 0.5: 50%<br><br>Zim: 0.10: 33% 0.15: 33% 0.30: 33%	Distribution is chosen to reflect the range of settings and to encompass potential future higher levels of PrEP uptake and use.
pr_prep_oral_b<br>*pr_prep_inj_b*	If a person is willing to take oral PrEP (prep_oral_willing = 1) and has tested negative and meets the criteria for risk-informed PrEP and is not “hard to reach” then they	0.1:33% 0.3:33% 0.7:33%<br>Zim: 0.05: 50% 0.1: 50%	As above, distribution is chosen to reflect the range of settings and to encompass

Parameter name^	Description	Distribution sampled (value; % with value)	Motivation for distribution
	will start PrEP in the period with probability *pr_prep_oral_b* (with the exception that during the scale-up period lower values are used). Similarly for cab-la PrEP. When cab-la PrEP is available the person will start the PrEP type according to which of pref_prep_oral and pref_prep_inj has the highest value.		potential future higher levels of PrEP uptake and use.
pref_prep oral<br><br>*rate_choose_stop_prep_inj*	Rate of discontinuing PrEP per 3 months (person's choice to stop despite risky condomless sex)	0.05: 80% 0.15: 10% 0.30: 10%<br><br>0.05: 10% 0.15: 10% 0.30: 10%	To reflect likely variation by setting. Resulting outputted overall levels of PrEP persistence can be compared with those observed in various settings.
prob_prep_any_restart_choice	Probability of restarting PrEP after previous discontinuation when still having risky condomless sex (not differential by whether oral or inj PrEP)	0.05: 33% 0.10: 33% 0.20: 33%	As above
adh_pattern_prep_oral	Each person has a fixed long term tendency to adhere to ART that applies should they become infected with HIV and start ART. The degree to which this tendency to adhere is modified if the person is taking PrEP compared with if they had HIV and were taking ART is determined by this parameter.	Similar to ART adherence: 100%	Likely to be a strong person-specific component to adherence. Given data on viral suppression, adherence to ART is known to be high in the majority of people on treatment. Adherence to risk-informed oral PrEP may be similar.
rel_prep_oral_adh_younger	We assume that 50% of adolescents and young adults (age 15-24 years) will have an oral PrEP adherence *rel_prep_oral_adh_younger* times that for older adults.	0.8: 100%	AGYW known to be on average less adherent to ART than older women. (Yun 2018)
prep_oral_efficacy<br><br>*prep_inj_efficacy*	Oral PrEP efficacy (with 100% adherence)<br><br>Cab-la PrEP efficacy	0.90: 20% 0.95: 80%<br><br>0.90: 20% 0.95: 80%	(Heffron 2018, Delany-Moretlwe)
oral_prep_eff_3tc_ten_res	Effect on efficacy of oral PrEP when partner's virus has M184V and K65R mutations	0.25 times: 50% 0.5 times: 50%	(Cong 2013)
prep_inj_effect_inm_partner	Cab-la PrEP is assumed to be less efficacious (efficacy x *prep_inj_effect_inm_partner*) when the sexual partner with HIV to whom the subject is exposed has virus with an integrase inhibitor resistance mutation.	0:33% 0.25: 33% 0.5:33%	No data available to our knowledge. Sampled from a wide range to reflect uncertainty.

Parameter name^	Description	Distribution sampled (value; % with value)	Motivation for distribution
sens_tests_prep_inj	Regular rapid 3rd generation antibody tests have particularly low sensitivity. For this reason it has been proposed that it may be necessary to use RNA-based tests to in people starting cab-la PrEP to rule out HIV, and perhaps to also continue using such tests for the regular 3 monthly tests (if variable hivtest_type_1_prep_inj=1). We consider four options of sensitivity of such tests in people exposed to cab-la PrEP determined by the parameter *sens_tests_prep_inj*. Each of these involves specification of sensitivity of RNA-based tests (referred to as "type1") according to the time since HIV infection (primary, 0.25 years, 0.5 years or more) in people on cab-la. Test sensitivity for 3rd generation tests in people on cab-la is assumed the same for each of the four options<br>In primary infection = 0<br>3-6 months from infection = 0<br>≥ 6 months from infection = 0.25	Option 1:25% option 2:25% option 3:25% option 4:25%<br><br>Test sensitivity for RNA-based tests:<br><br>Option 1<br>In primary infection = 0.7<br>3-6 months from infection = 0.85<br>≥ 6 months from infection = 0.95<br><br>Option 2<br>In primary infection = 0.5<br>3-6 months from infection = 0.7<br>≥ 6 months from infection = 0.8<br><br>Option 3<br>In primary infection = 0.3<br>3-6 months from infection = 0.5<br>≥ 6 months from infection = 0.7<br><br>Option 4<br>In primary infection = 0.2<br>3-6 months from infection = 0.3<br>≥ 6 months from infection = 0.5	Eshleman 2022 x 2, Marzinke 2021 x 2. Sampled from wide ranges to reflect uncertainty.
cab_time_to_lower_threshold_g	We consider two possible distributions across individuals of the time for cabotegravir concentrations to decline below a level relevant for resistance risk after stopping cab-la.	1: 50% 2: 50%<br><br>With 1, 67% have time 0.25 years, 33% have time 0.5 years<br><br>With 2, 50% have time 0.25 years, 40% have time 0.5 years, 10% have time 1.0 years.	Cabotegravir tail known to be long but unknown what concentration is needed to pose continued risk for resistance development if HIV is present (Landovitz 2020 tail).
sens_vct_testtype3_cab_tail	Sensitivity for 3rd generation antibody testing in a person with HIV in the cab-la "early tail"	0.5 : 33% 0.8 : 33% 0.98 : 33%	Wide distribution to reflect uncertainty due to lack of data.
testt1_prep_inj_eff_on_res_prim	Due to earlier detection of HIV and more rapid ART initiation, RNA testing for people on PrEP throughout is assumed to lead to lower probability of acquiring a given integrase resistance mutation for a person in primary infection while on Cab-LA, by an amount determined by this parameter..	0.25: 33% 0.5: 33% 0.75: 33%	Eshleman 2022 x 2. Wide distribution to reflect uncertainty due to lack of data.

Parameter name^	Description	Distribution sampled (value; % with value)	Motivation for distribution
pr_inm_inj_prep_primary	This determines the risk (for each of the 5 integrase inhibitor mutations) that a person in primary infection while dosed with cabotegravir (due to cab-la PrEP being started when in the primary infection window period in which HIV was not detected, or due to breakthrough infection during cab-la) develops resistance to cabotegravir in this 3 month period of primary infection.	0.1: 25% 0.2: 25% 0.3: 25% 0.5: 25%	Marzinke 2021 x 2. High uncertainty reflected in wide distribution.
incr_res_risk_cab_inf_3m	The extent to which risk of integrase inhibitor resistance is increased in the 3 month period after primary infection, for a person on cab-la or in the early cab-la tail.	1:17% 3:17% 5:17% 10:17% 20:17% 50:17%	To reflect uncertainty. This parameter included to ensure that we consider fully the potential risk of integrase inhibitor resistance due to starting cab-la when in early infection.
rel_pr_inm_inj_prep_tail_primary	Determines the relative risk (compared with *pr_inm_inj_prep_primary* mentioned above) that a person who becomes infected with HIV who is in the early cabotegravir tail period develops resistance to cabotegravir.	0.25: 20% 0.5: 20% 0.75: 20% 1: 20% 1.33: 20%	Currently there is substantial uncertainty due to lack of data.
**Parameters relating to transmission**			
fold_change_w<br><br>*fold_change_yw*	The fold difference in female to male transmission rate compared with male to female, for a given viral load.<br><br>Rate is higher in younger women by *fold_change_yw*.	1: 5% 1.5: 25% 2: 70%<br><br>1: 33% 3: 33% 5: 33%	Informed by the higher incidence and prevalence in women in younger age groups and some direct evidence. (Masson 2015, Nicolosi 1994)
fold_change_sti	The fold difference in HIV acquisition risk for a person with a current STI.	2: 50% 3: 50%	Multiple studies show a raised risk of acquisition but uncertainty over the effect size. (Cohen 1998)
fold_tr	A higher or lower risk of acquiring HIV for a given viral load in the partner	0.67: 33% 1: 33% 1.5: 33%	The convey uncertainty in the estimate of transmission rates
fold_tr_newp	Fold transmission rate per 3 months for short-term partners compared with long-term partners	0.3:9% 0.4:9% 0.5:9% 0.6:9% 0.7: 9% 0.8:9% 0.9:9% 1.0:9% 1.25:9% 1.66:9% 2.5:9%	Uncertainty over this – considered more likely to be lower than higher due to assumed lower number of sex acts in the 3 month period with short-term partners than long term

Parameter name^	Description	Distribution sampled (value; % with value)	Motivation for distribution
res_trans_factor_nn	Parameter affecting the probability that if NNRTI resistance mutation present in source partner that this is not present/detectable in virus new host	0.5: 20% 0.7: 20% 0.8: 20% 0.90: 20% 1.00: 20%	Informed by the values needed to lead to the range of transmitted NNRTI resistance observed (see Table 1 in paper)
res_trans_factor_ii	Parameter affecting the probability that if integrase inhibitor resistance mutation present in source partner that this is not present/detectable in virus new host	0.2: 25% 0.4: 25% 0.6:25% 0.8:25%	Little data available to inform this yet – we make the assumption in 80% of runs that transmission occurs. Bailey 2007, Ndashimye 2021
tr_rate_undetec_vl	*Rate of acquisition of HIV from a condomless partner with undetectable VL*	0.0: 70% 0.0001: 20% 0.001: 10%	To express range of uncertainty.
super_infection<br><br><br>*super_inf_res*	Whether we consider super-infection (which means a person with HIV can acquire HIV drug resistant HIV through a subsequent infection with a new viral strain).<br><br>If super-infection can occur, the probability that resistance mutations in the source partner become present in the subject.	Occurs in 50% of runs.<br><br><br>0.2: 90% 0.8: 10%	Super-infection can occur but its significance is uncertain but unlikely to be substantial. (Smith 2005)
**Parameters relating to pre-ART care and development of TB and WHO stage 4 diseases**			
fx	Multiplicative factor to alter the average rate of CD4 count decline in natural HIV progression (which thus alters the incubation period distribution).	0.7: 20% 0.85: 20% 1.0: 20% 1/0.85: 20% 1/0.7: 20%<br><br>Zim: 0.8	Derived based on consideration of evidence from natural history studies (Pantazis 2005, Sabin 2000, Hubert 2000, O'Brien 1998, Henrard 1995)
gx	Multiplicative factor allowing expression of uncertainty in rates of viral load increase over time in people untreated	1.0: 33% 1.5: 33% 3.0: 33%	There is uncertainty over rates of viral load increase (Pantazis 2005)
prob_loss_at_diag<br><br><br>*prob_lossdiag_adctb*<br>*prob_lossdiag_who3e*	Probability that a person is immediately lost after initial HIV diagnosis.<br><br>...if has an AIDS disease or TB at time of diagnosis<br>...if has an AIDS disease or TB at time of diagnosis	0.02: 20% 0.05: 30% 0.15: 30% 0.35: 10% 0.50: 10%<br><br>Beta(5,95)<br>Beta(15,85)	e.g. (Rosen 2011)
rate_lost	For people under care yet to start ART or previously have taken ART, the rate of being lost to care per 3 mths.	0.2: 33% 0.35: 33% 0.5: 33%	Uncertain and will vary by setting. Distribution chosen to reflect this. This is one of the parameters influencing the proportion of diagnosed people on ART.

Parameter name^	Description	Distribution sampled (value; % with value)	Motivation for distribution
rate_return	Probability of return to care for a person who has been diagnosed with HIV (and may have started ART) but is now lost and not on ART, without current WHO stage 3 or 4 disease, per 3 months.	0.01: 15% 0.05: 15% 0.10: 40% 0.3: 15% 0.60: 15%	As above
prob_return_adc	Probability of return to care for a person who has been diagnosed with HIV (and may have started ART) but is now lost and not on ART and has a WHO stage 4 condition. This is a probability that operates just for the 3-month period that the events occurs.	0.7: 20% 0.8: 30% 0.9: 50%	As above
rate_loss_persistence	Rate of loss from majority virus of transmitted resistance mutations (per 3 months)	0.00: 10% 0.005: 10% 0.01: 10% 0.015: 40% 0.02: 30%	(e.g. Jain 2011, Yang 2015)
prob_supply_interrupted<br><br>*prob_supply_resumed*	Probability per 3 months that ART is interrupted (/ resumed) as a result of drug stock-out<br>Probability per 3 months that ART is resumed after interruption as a result of drug stock-out	0.003: 100%<br><br>0.8: 100%	Stock-outs generally now seems low in countries of focus in sub-Saharan Africa.
rate_loss_acq_nnm_offart	The rate with which NNRTI mutations are lost from majority virus when a person with an NNRTI mutation in majority virus interrupts ART	0.05: 100%	Indirectly informed by detection of NNRTI mutations in people who remain off ART.
fold_change_151_risk	Fold change in the risk of cross-NRTI mutation at codon 151	1: 100%	Included to allow possibility to vary
is_red_activity	Extent to which number of active drugs is under or over-estimated by interpretation systems	0: 100%	Included to allow possibility to vary
sd_patient_cd4_rise_art	Inter-patient variation in rate of CD4 rise - when CD4 is rising	0.2: 100%	CD4 count increases on ART are informed by comparisons with data described in section entitled *Variability in individual (underlying) CD4 counts for people on ART*
prob_cd4_meas_done	When people on ART were monitored with CD4 count measurement, probability that measure was done when due.	0.85: 100%	Assumed good implementation of viral load monitoring.
cm_1stvis_return_vlmg1000	Whether the policy is for CD4 count to be measured each time a person comes back into care or have viral load > 1000 cps/mL, to see if have advanced HIV disease (as in WHO guidelines)	1 (yes): 100%	Recommended by WHO as part of package of care for advanced HIV disease.
crag_cd4_l100<br><br>*crag_cd4_l200*	whether cryptococcal antigen testing done if measured CD4 count < 100 / 200	0 (no): 100%<br><br>0 (no): 100%	Will be explored in future analyses

Parameter name^	Description	Distribution sampled (value; % with value)	Motivation for distribution
tblam_cd4_l100<br><br>*tblam_cd4_l200*	whether tblam test done if measured cd4 count < 100 / 200	0 (no): 100%<br><br>0 (no): 100%	Will be explored in future analyses
prob_who3_diagnosed<br><br>*prob_who4_diagnosed*	When, in the past, "clinical monitoring" was used to decide on switching from 1st to 2nd line ART, it was necessary to model whether a person having a WHO stage 3 or 4 condition had that condition diagnosed and thus had the possibility of the switch being made.	0.50: 100%<br><br>0.80: 100%	Included to allow possibility to vary
sd_vl_whb<br><br>*decr_sd_vl_whb*<br><br>*vl_whb_offset*	Parameters relating to viral load measurement performance when using whole blood in the form of dried blood spots (DBS)	0.50: 100%<br><br>0.05: 100%<br><br>0.0: 100%	Discussed in detail in Phillips et al 2016 OFID
dol_higher_potency	The potency of a drug is the contribution to regimen activity if resistance to the drug is not present. For all NRTIs the value is 1, for boosted protease inhibitors it is 2, for efavirenz, dolutegravir and cabotegravir we sample from alternative values.<br><br>Potency of dolutegravir and efavirenz and cabotegravir (only used in people on cal-la PrEP who do not have their HIV diagnosed) is assumed equal and sampled between 1.5 and 2.	1.5: 50% 2:50%	Dolutegravir has substantial efficacy as monotherapy, albeit insufficient to be recommended. Dolutegravir and efavirenz appear of equal potency in the Advance trial (Venter 2019) (although a different risk of resistance)
ntd_risk_dol	Risk of neural tube defect in baby of woman on dolutegravir at conception	0.0022: 100%	Zash 2019
incr_death_rate_crypm<br>*incr_death_rate_sbi*<br>*incr_death_rate_tb*<br>*incr_death_rate_oth_adc*	Relative increase in risk of death (beyond that indicated by the current CD4 count, viral load, and age, and whether on ART) for a person with cryptococcal meningitis.<br><br>Similarly for serious bacterial infection (SBI), tb, and other WHO stage 4 condition (oth_adc)	3: 33% 5:33% 10:33%<br>1.5: 33% 2:33% 3:33%<br>1.5: 33% 2:33% 3:33%<br>1.5: 33% 2:33% 3:33%	To reflect uncertainty.
rr_non_aids_death_hiv_off_art<br><br>rr_non_aids_death_hiv_on_art	Parameters to reflect the increased risk of non-AIDS death in people with HIV, with a greater effect in those not on ART.	1.5: 33% 2: 33% 3: 33%<br><br>1.1: 33% 1.3: 33% 1.5: 33%	Phillips et al 2016

Parameter name^	Description	Distribution sampled (value; % with value)	Motivation for distribution
prop_adc_crypm<br>*prop_adc_sbi*	We determine risk of WHO stage 4 condition (AIDS defining condition ADC) occurring and then if one occurs we determine whether it is due to cryptococcal meningitis or SBI.	0.15: 100%<br>0.15:100%	Hakim 2017. Mfinanga 2015.
rate_crypm_proph_init<br>*rate_tb_proph_init*<br>*rate_sbi_proph_init ;*	Rate of initiation of prophylaxis against cryptococcal meningitis and TB	0: 100%<br>0: 100%<br>0: 100%	Placeholder values. To be varied in future analyses.
effect_tb_proph effect_crypm_proph<br>*effect_sbi_proph*	Effect of TB prophylaxis on risk of TB.<br>The same for cryptococcal meningitis and SBI	0.5: 100%<br>0.5: 100%<br>0.5: 100%	Placeholder values. Prophylaxis is not implemented currently. To be varied in future analyses in which prophylaxis is implemented.
tb_base_prob_diag_l<br>*crypm_base_prob_diag_l*<br>*sbi_base_prob_diag_l*<br>*oth_adc_base_prob_diag_l*	Base probability that tb is diagnosed late rather than early (which has a consequence for the risk of death)	0.25: 33% 0.50: 33% 0.75: 33%<br>0.25: 33% 0.50: 33% 0.75: 33%<br>0.25: 33% 0.50: 33% 0.75: 33%<br>0.25: 33% 0.50: 33% 0.75: 33%	Variation and uncertainty over this so sample from a wide range of values.
tblam_eff_prob_diag_l<br>*crag_eff_prob_diag_l*	If tested on a TB LAM test and have TB, the effect this has on the probability that the TB is diagnosed late rather than early	0.5: 100%	To be varied when TB lam tested is switched on in model in future investigations.
rel_rate_death_tb_diag_e<br>*rel_rate_death_crypm_diag_e*<br>*rel_rate_death_sbi_diag_e*<br>*rel_rate_death_oth_adc_diag_e*	Relative increase in rate of death from TB event if it is diagnosed late . The same for cryptococcal meningitis and SBI<br>and other WHO stage 4 conditions (oth_adc)	0.50: 33% 0.67:33% 0.80:33%<br>0.50: 33% 0.67:33% 0.80:33%<br>0.50: 33% 0.67:33% 0.80:33%<br>0.50: 33% 0.67:33% 0.80:33%	Expressing substantial uncertainty and variability
effect_visit_prob_diag_l	Given a WHO stage 4 or TB event, relative risk of being diagnosed late if currently under care (i.e. visit = 1)	0.50: 33%<br>0.67: 33%<br>0.80: 33%	Expressing substantial uncertainty and variability
**Parameters relating to people on ART**			
adh_pattern	Population adherence profile; described in terms of the proportion having a given average adherence and period-to-period variability in adherence. Note that adherence is additionally affected by age and gender.	1: 5% 2: 55% 3: 10% 4: 10% 5:10% 6: 5% 7:5%<br><br>Zim: 1: 5% 2: 55% 3: 20% 4: 10% 5:10%	Reflection of wide range of adherence profiles in different settings, informed by differences in proportions of people on ART with viral load suppression. This range leads to a range of levels of viral suppression and of resistance.

Parameter name^	Description	Distribution sampled (value; % with value)	Motivation for distribution
red_adh_tb_adc<br><br><br>*red_adh_tox_pop*<br><br><br>*add_eff_adh_nnrti*<br><br><br><br>*red_adh_multi_pill_pop_*<br><br><br><br>*altered_adh_sec_line_pop*	Reduction in adherence to ART associated with currently having an AIDS defining condition / TB<br><br>The extent to which drug toxicity influences adherence to ART negatively.<br><br>NNRTI drugs tend to have a longer half-life than PIs – this indicates the gain in effective adherence from NNRTIs due to this effect.<br><br>The extent to which taking multiple ARVs separately tends to lead to lower adherence than a single once daily pill.<br><br>The extent of any increase in adherence in people switched to second line ART – this is in addition to any effects of enhanced adherence counselling after a measured viral load > 1000 copies/mL.	logNormal(0.1, 0.5)<br><br><br>logNormal(red_adh_tox_pop_v, 0.5)<br>where red_adh_tox_pop_v = 0.05 50% 0.10 50%<br>logNormal(0.10, 0.30)<br><br><br><br>logNormal(red_adh_multi_pill_pop_v, 0.5)<br>where red_adh_multi_pill_pop_v = 0.05 50%<br>0.10 50%<br><br>logNormal(0.05, 0.05)	(Heestermans 2016)
pr_art_init	Probability of ART initiation per 3 months in a person in care who is eligible according to current criteria.	0.5: 20% 0.7: 20% 0.9: 20% 0.95: 20% 1.0: 20%	These parameters contribute to determine the proportion of HIV diagnosed people who are on ART. The distributions are chosen such that combinations of these parameters lead to observed proportions of HIV diagnosed people on ART (e.g. PHIA surveys)
prob_lost_art	For a person who interrupts / stops ART the probability that they are simultaneously lost from care.	0.5: 20% 0.6: 20% 0.7: 20% 0.8: 20% 0.9: 20%	(e.g. McMahon 2016)
rate_restart	Rate of restart of ART for people who previously have been on ART and have returned to care, per 3 months.	0.8: 25% 0.85: 25% 0.9: 25% 0.95: 25%	Assumed to be high, given the person has returned to care. Most people who are regularly seen in clinics who have previously started ART are on ART.
rate_int_choice	Rate of interruption / stopping of ART per 3 months. Also influenced by current drug toxicity and underlying tendency to adhere.	0.002: 30% 0.004: 30% 0.008: 30% 0.02:5% 0.05: 5%<br><br>Zim: 0.002: 33% 0.003: 33% 0.004: 33%	(McMahon 2016)

Parameter name^	Description	Distribution sampled (value; % with value)	Motivation for distribution
incr_rate_int_low_adh	Parameter indicating the extent to which people with a long-term average adherence in the lowest group have a multiplicatively increased risk of ART interruption. Effect of current low adherence on risk of treatment interruption / discontinuation.	1: 50% 2: 25% 5: 25%	(Agbaji 2015)
pr_switch_line	Probability of switch to second line per 3 months in a person who has fulfilled the failure criteria for first line failure.	0.10:25% 0.20:25% 0.5:25% 1.0:25%	(Fox 2012, Johnston 2012, PHIA surveys)
clinic_not_aw_int_frac	If a person interrupts ART, the probability that this is not disclosed to the clinic and they are classified as being on ART	0.1: 20% 0.3: 20% 0.5: 20% 0.7: 20% 0.9: 20%	Uncertain and will vary by setting, hence a broad distribution.
fold_change_mut_risk	Fold difference in rate of accumulation of mutations (for all drugs) compared with base case.	1: 80% 2: 10% 0.5: 10%	To consider that the rate of resistance mutation acquisition is higher or lower than the rate assumed, reflecting some uncertainty. This relates to all resistance mutations.
*rate_res_ten_*	Parameter reflecting the rate of acquisition of tenofovir resistance. The value of 0.1 was derived based on European cohort data and the value of 0.3 reflects the potentially higher value for subtype C in southern Africa.	0.1: 33% 0.2: 33% 0.3: 33%	(Tenores 2016)
poorer_cd4rise_fail_nn<br><br>*poorer_cd4rise_fail_ii*	This indicates the extent of poorer CD4 rise per 3 months on failing NNRTI based regimens (compared with PI)<br>This indicates whether the poorer CD4 rise also applied to failing INSTI based regimens	Normal(-6,3)<br><br>no: 50% yes: 50%	(Ledergerber 2004)
adh_effect_of_meas_alert	The effect of having a viral load measured > 1000 copies/mL on adherence, due to the enhanced adherence intervention.	0.35:15% 0.70: 70% 0.90: 15%	Uncertainty over the effect size.
prob_vl_meas_done	Probability of a viral loead measure being done. This probability operates for each time a viral load is due to be tested.	0.0: 5% 0.1: 30% 0.7: 50% 1.00: 15%	Variation in viral load implementation in different settings. Note that in half of settings with value 0 there is CD4 count monitoring of people on ART in place.
cd4_monitoring	If viral load monitoring is not being implemented (*prob_vl_meas_done* = *0*), if CD4 count monitoring being done ?	if prob_vl_meas_done = 0 then<br>no: 50% yes 50%	This will vary by setting.
switch_for_tox	Whether the ART program manages to implement drug substitutions in response to specific toxicities experienced by patients.	No: 80% Yes: 20%	This will vary by program but generally not widespread.

**Parameter name^**	**Description**	**Distribution sampled (value; % with value)**	**Motivation for distribution**
zero_3tc_activity_m184	activity of 3TC in presence of M184V mutation	No: 80% Yes: 20%	To consider alternative assumptions; distribution broadly reflects the uncertainty.
zero_ten_activity_k65	activity of tenofovir in presence of K65R mutation	No: 80% Yes: 20%	To consider alternative assumptions; distribution broadly reflects the uncertainty.
higher_rate_res_dol	Whether there is a higher rate of resistance to dolutegravir than the base assumption (i.e. 4 times lower than efavirenz compared with 13 times lower in base case).	No: 80% Yes: 20%	To consider alternative assumptions; distribution broadly reflects the uncertainty.
*prop_bmi_ge23_*	Proportion of people initiating dolutegravir who have BMI $\geq$ 23	0.5: 50% 0.75: 50%	Uncertainty over the proportion of the population starting dolutegravir who have BMI > 23 and hence a possible negative effect of weight gain on dolutegravir. (Msyamboza 2011, Thorogood 2007)
*incr_mort_risk_dol_weightg_*	Rate ratio for mortality in people on dolutegravir who had BMI $\geq$ 23 at start, due to dolutegravir induced weight gain.	1: 1% 1.1: 16% 2: 17% 2.1: 17% 2.2: 17% 3.0: 17% 4.0: 15%	Wide distribution within plausible bounds reflecting uncertainty (Berrington 2010, Flegal 2013, Achhra 2018, Kivimaki 2017)
nnrti_res_no_effect	Effect of NNRTI resistance mutations on activity of efavirenz (base case: K103N 0 activity, G190A 0.25 activity, Y181C 0.25 activity)	0.25: 25% 0.5: 5% 0: 75%	Some uncertainty over this. With this distribution the average odds ratio for VL > 1000 at 1 year from start of ART associated with pre-treatment NNRTI drug resistance = 3.3 (compared with 3.9 in a recent meta-analysis (Kanters 2019)
tox_weightg_dol	Whether weight gain is treated as a toxicity that has an associated increased risk of ART interruption	no: 50% yes: 50%	Weight gain does not seem to be mentioned as troublesome to people on dolutegravir, but this could change with time.
*rel_dol_tox_*	Relative rate of neurologic toxicity (sleep disturbance for dolutegravir and dizziness and vivid dreams for efavirenz)	0.5 fold that of efavirenz 80%<br>Equal to efavirenz 20%	While evidence suggests neurologic toxicity is higher with efavirenz, there is uncertainty over size of effect of insomnia with dolutegravir so we consider the possibility that the overall neurologic toxicity of the two drugs could be equal.

Parameter name^	Description	Distribution sampled (value; % with value)	Motivation for distribution
double_rate_gas_tox_taz	Parameter related to the rate of gastrointestinal toxity relating to atazanavir. Whether base rate is doubled or not.	Yes: 50% No: 50%	Uncertainty over gastrointestinal toxicity relating to atazanavir – consider possibility that this has been underestimated.
lower_future_art_cov	Whether future coverage of ART is below that predicted by continuation in current trend in rates.	No:93% Yes: 7%	To reflect uncertainty.
rr_int_tox	Increased rate of ART interruption according to presence of a drug toxicity.	2-fold: 33% 10-fold: 33% 30-fold: 33%	Consider possibility that ART interruption is substantially more highly related to drug toxicity than base case
greater_disability_tox	Parameter to allow consideration of a greater disability weight associated with drug toxicity (0.25) compared with the base assumption (of 0.05)	No: 50% Yes: 50%	To reflect the uncertainty and perceived relatively low likelihood that value is as high as 0.25.
*greater_tox_zdv_*	Whether the toxicity associated with zidovudine is greater than the base assumption.	No: 33% Yes, 2-fold: 33% Yes, 4-fold: 33%	https://clinicalinfo.hiv.gov/en/guidelines/pediatric-arv/zidovudine
zdv_potency_p75	Whether potency of zdv is 0.75 of an active drug rather than 1.	No: 50% Yes: 50%	To reflect uncertainty
higher_newp_less_engagement	Whether there is a tendency for people with more short term partners to be less likely to be engaged with ART care	No: 80% yes: 20%	Likely to vary by setting
reg_option_104	Indicator of which regimen sequencing strategy is used from 2021: whether all people on ART are switched to TLD or whether it is only new ART initiators in which TLD is used.	All switch: 50% New initiators only: 50%	WHO guidelines say new initiators only but several countries are switching in all.
reg_option_107_after_cab	Indicator of whether there is a policy that a person who becomes diagnosed with HIV having previously been on cab-la is started on an atazanavir/r based regimen rather than dolutegravir.	No: 80% Yes: 20%	Uncertainty about whether it will be possible to implement such a policy.
pr_res_dol	Probability of each integrase inhibitor drug resistance mutation emerging in a given 3 month period in which the criteria for *newmut* is met (see above).	0.005 : 33% 0.01 : 33% 0.015 : 33%	Rate of emergence of dolutegravir resistance is low (Walmsley 2013, 2015, Venter 2019), even in the context of dolutegravir monotherapy (albeit that the risk is too high for clinical use as monotherapy) (Fournier 2022, Mbhele 2021, Rossetti 2022)
rr_res_cab_dol	Relative risk of an integrase inhibitor mutation arising with cabotegravir compared with dolutegravir, for a given number of active drugs, adherence_drug level, viral load.	1: 33% 1.5: 33% 2:33%	Oliveira 2018

Parameter name^	Description	Distribution sampled (value; % with value)	Motivation for distribution
res_level_dol_cab_mut	Degree to which full activity of dolutegravir is diminished as a result of presence of one of the primary integrase inhibitor resistance mutations (1.00 means there is no residual antiviral effect when a resistance mutation is present).	0.75: 80% 1.00: 20%	https://hivdb.stanford.edu/hivdb/by-patterns/
**Parameters relating to pregnancy**			
prob_pregnancy_base	Parameter determining base rate of pregnancy for women having condomless sex (to which there is an effect of age)	Uniform (0.06, 0.05)<br>if inc_cat = 1 then prob_pregnancy_base increased 1.75-fold<br>if inc_cat = 3 then prob_pregnancy_base decreased 1.75 -fold	Variability between settings in fertility rate.
rate_birth_with_infected_child	Parameter determining the risk of mother to child transmission (MTCT), for a given level of mother viral load.	0.3: 5% 0.4: 25% 0.5: 60% 0.6: 10%	To produce plausible variation in the MTCT rate.
*oth_dol_adv_birth_e_risk_*	Risk of dolutegravir-induced adverse birth event, due to dolutegravir-induced weight gain	0.0005: 20% 0.0015: 40% 0.002: 20% 0.003: 20%	Wide distribution within plausible bounds reflecting uncertainty. (Cresswell 2012)
Parameters related to sex worker disadvantages			
sw_higher_prob_loss_at_diag<br><br>*sw_higher_int*<br><br>*rel_sw_lower_adh*	Multiplicative factor increasing risk of being lost at diagnosis<br><br>Multiplicative factor increasing risk of interruption<br><br>Multiplicative factor applied to adherence levels	2: 33% 5:33% 10: 33%<br><br>2: 25% 5:25% 10: 25% 20:25%<br><br>0.8: 50% 0.9: 50%	Sampling widely to consider various levels of disadvantage
**Parameters relating to sex worker programmes**			
sw_program	Whether a program for sex workers is in place.	100% Key Populations since 2010	Specific programme exists in Zimbabwe (e.g. Cowan 2018)
effect_sw_prog_newp;	Effect of sex worker program on:<br><br>levels of condomless sex	0.05: 50% 0.1: 50%	Sampling widely to consider various program effects

Parameter name^	Description	Distribution sampled (value; % with value)	Motivation for distribution
effect_sw_prog_6mtest	whether 6 monthly HIV testing is done	0.05: 33% 0.10: 33% 0.15: 33%	
effect_sw_prog_prep	multiplicative factor in the propensity to take PrEP	0.05: 50% 0.10: 50%	
effect_sw_prog_pers_sti	multiplicative factor on probability of any STI lasting >3 months	0.10: 50% 0.20: 50%	
Impact on sex worker disadvantages:			
effect_sw_prog_int;	the rate of interruption of ART for women on ART	0.3: 33% 0.5: 33% 0.7: 33%	
effect_sw_prog_adh	reduction in adherence to ART for women on ART	0.10:33% 0.15: 33% 0.25: 33%	
effect_sw_prog_lossdiag	the probability of not engaging with care for women at time of HIV diagnosis	0.3: 33% 0.5: 33% 0.7: 33%	
**Parameter for COVID-19 death risk**			
cov_death_risk_mult	There is a calendar time limited between April 2020 and Sept 2021 risk of death from COVID-19 which is age dependent. The base risk per 3 months during the epidemic is as follows<br><br>if 15 <= age < 20 then cov_deathrix = 0.0001<br>if 20 <= age < 30 then cov_deathrix = 0.0003<br>if 30 <= age < 40 then cov_deathrix = 0.0008<br>if 40 <= age < 50 then cov_deathrix = 0.0016<br>if 50 <= age < 60 then cov_deathrix = 0.006<br>if 60 <= age < 70 then cov_deathrix = 0.019<br>if 70 <= age < 80 then cov_deathrix = 0.043<br>if 80 <= age then cov_deathrix = 0.078<br><br>This parameter allows us to consider uncertainty in the form of a relative risk to allow consideration of higher risk.	1: 0.4 2: 40% 3: 20%	To reflect uncertainty

Parameter name^	Description	Distribution sampled (value; % with value)	Motivation for distribution
**Parameters relating to local community TLD access**			
prob_prep_pop_wide_tld	The probability of starting TLD PEP / PrEP in a given 3 -month period for a person who is PrEP willing and has an indication for PEP / PrEP, when local community TLD access is available.	0.02: 33% 0.05: 33% 0.1: 33%	Unknown – information to be obtained from pilot studies
inc_oral_prep_pref_pop_wide_tld	The proportion by which a person's PrEP preference value for oral PEP/PrEP moves towards one when local community TLD access is introduced.	0.1: 33% 0.3: 33% 0.5:33%	Unknown – information to be obtained from pilot studies
prop_pep	Under local community TLD access, the proportion of PEP/PrEP use in people with a PEP/PrEP indication that is as PEP rather than PrEP.	0.2: 33% 0.5: 33% 0.8: 33%	Unknown – information to be obtained from pilot studies
pep_effiacy	The efficacy of PEP for HIV prevention (i.e. with full adherence) .	0.9: 80% 0.95: 20%	Irvine 2015.; Massud et al.
pop_wide_prep_adh_effect	effect of taking pop wide TLD without clinical supervision (indicated by having no supervised test this period (tested=0)) on prevention *effectiveness*	Lower effectiveness by 25%:<br>Lower effectiveness by 11%:<br>No effect: 60%<br>Higher effectiveness by 10%<br>Higher effectiveness by 10%	Unknown – would perhaps be partially informed by pilot studies
rr_interrupt_pop_wide_tld<br><br>*rr_return_pop_wide_tld*	The degree to which the rate of interruption of ART is reduced with local community TLD access.<br><br>The degree to which the rate of restarting ART in people who had discontinued is reduced with local community TLD access.	1/1.5: 30% 1/2: 30% 1/3: 30% 1/5: 10%<br><br>1.5: 25% 2:25% 3:25% 5: 25%	Unknown – would perhaps be partially informed by pilot studies
pop_wide_tld_selective_hiv	People that have never tested for HIV in a clinical context may use TLD if they believe they have HIV, possibly based on use of a self-test kit available in local communities with TLD. There could be some people who are HIV negative who start TLD due to a false positive self-test or due to thinking they have HIV without testing. This parameter indicates how many times greater the probability of a person with HIV starts TLD compared with a person without HIV.	5 times: 33%% 10 times: 50 times	Unknown – would perhaps be partially informed by pilot studies
prob_tld_if_untested	The probability per 3 months of a person who has never tested for HIV but has had at least one short-term condomless partner in the past starting TLD. This is for a person with HIV. The probability is	0: 33% 0.001:33% 0.005:33%	Unknown – would perhaps be partially informed by pilot studies

Parameter name^	Description	Distribution sampled (value; % with value)	Motivation for distribution
	pop_wide_tld_selective_hiv times lower for a person without HIV.		
prob_test_pop_wide_tld_prep	Some people who are taking PEP/PrEP due to community access may nevertheless be tested each three months under clinical supervision (tested=1. This gives the probability per three months of this occurring.	0.1: 33% 0.25: 33% 0.5: 33%	Unknown – would perhaps be partially informed by pilot studies
prob_onartvis_0_to_1<br><br>*prob_onartvis_1_to_0*	These are the probabiltiies that a person who is on ART under clinical care will transition to self-taking ART (and the reverse) and hence not being monitored or receiving any benefits of enhanced adherence counselling.	0.02: 25% 0.05: 25% 0.1: 25% 0.2:25%<br><br>0.005: 25% 0.01: 25% 0.03: 25% 0.05: 25%	Unknown – would perhaps be partially informed by pilot studies
artvis0_adh	effect of being on ART but not under clinical monitoring on adherence, beyond the effects of not having viral load tested and thus not having enhanced adherence counselling if VL > 1000.	No effect: 80%<br>Lower adherence: 20%	Unknown – would perhaps be partially informed by pilot studies
death_r_iris_pop_wide_tld	Additional absolute risk of IRIS when starting ART with CD4 count < 100 and not under clinical supervision	0.01: 33% 0.03: 33% 0.05: 33%	Sereti 2020 - *assumed higher risk due to not in care*

^ model program (in which these variable names are used) available on figshare (see main paper)
Parameters are randomly chosen from distributions as shown in the table above. The value of these parameters will impact on key epidemic characteristics such as HIV prevalence, incidence, proportion diagnosed, on ART and virally suppressed. The latter are all outputs of the model and as such are not directly inputted into the model.

Disability weights and costs

Table S28. Disability weights

Values are 1 in each three-month period except for the following:

Condition in current 3-month period	Disability weight for current 3-month period	Source
Diagnosed HIV (even if asymptomatic)	0.02	
Any drug toxicity in current 3-month period	0.05 (0.25 if greater_disability_tox =1)	(Salomon 2012)
Any WHO stage 3 condition (except TB) in current 3-month period	0.22	
TB in current 3-month period	0.40	
Any WHO stage 4 condition in current 3-month period	0.54	

Table S29. Unit Costs

Item	Unit Cost	Source / explanation
Drug costs per year:		CHAI 2022
TLE	$74 per year ($62 without supply chain costs	
TLD	$65 per year ($54 without supply chain costs)	
ZL-PI (PI atazanavir)	$259 per year ($216 without supply chain costs)	
ZLD	$103 per year ($94 without supply chain costs)	

Item	Unit Cost	Source / explanation
Cost of treatment of a WHO stage 4 condition over 3 months (cost is incurred for 3 months)<br><br>Cost of treatment of a WHO stage 3 condition over 3 months (cost is incurred for 3 months)<br><br>Cost of treatment of TB per 3 months (cost is incurred for 6 months)<br><br>Cotrimoxazole annual cost	$200<br><br><br><br>$20<br><br><br><br>$50<br><br><br>$5	Specific data not available on average unit costs of treating WHO stage 3 and 4 conditions and per clinic visit costs - costs used are informed by evidence synthesis from studies that cost according to current CD4 count of those in pre-ART care, cost of ART initiation, which also include costs of CD4 tests (Eaton 2014, Hyle 2025)
CD4 count measurement	$10	(Hyle 2014, Keebler 2014, Hyle 2025)
Viral load measurement:	$22	Human resource costs $3, sample collection consumables $2, relaying of results $2 (this costing information was provided by Médecins Sans Frontierès (MSF) (including equipment and other costs such as consumables, maintenance and shipping) $15. Updates are consistent with this cost (Global Fund)
Non-ART programme costs per year	$80 ($40 per year if on tiered care due to viral load < 1000)	Siapka 2014, Tagar 2014, Menzies 2012, Nichols 2021 x 2, Shiri at al 2021
Cost of the targeted adherence counselling intervention triggered by a viral load > 1000 copies/mL	$10	Assumption
HIV test (including personnel costs)	$3.70	Personal communication. CHAI.
Cost of providing free HIV self tests in the context of community availability of TLD for PEP / PrEP / ART.	$1 per 3 months per person self-taking PEP / PrEP in context of community availability of TLD.	Unknown extent to which these self test kits will be used.
Annual cost of treatment for a child born with HIV	$160	This cost was estimated based on a drug cost of $75 per year, a one-off cost of early infant diagnosis of $22, cost of viral load testing of $22 per year, costs of clinic visits of $40 or $80 per year (depending on whether viral load is suppressed), assuming 50% of

Item	Unit Cost	Source / explanation
		children will achieve viral suppression. This is likely to be a lower limit cost per year.
VMMC	$106	
Clinic-provided Oral PrEP (tenofovir + lamivudine)<br><br>Cab-LA PrEP<br><br>Self-administered TLD as PEP / PrEP	$116 per year ($29 per 3 months)<br><br>$144 per year ($36 per 3 months)<br><br>$65 per year ($16 per 3 months)	$29 per 3 months, consisting of a drug cost of $15 (including supply chain costs, based on the South Africa tender price for PrEP drugs, $4 for an HIV test per 3 months, and $10 per 3 months for additional costs necessary to facilitate education and access (including any costs of HBV or creatinine testing that might be done at first start).<br><br>For Cab-LA we used a similar drug cost ($15 per 3 months $10 per injection), but HIV test and clinic visit costs are 1.5 fold higher due to 6 visits per year rather than 4 (so $36 per 3 months in total, $144 per year).<br><br>TLD used as PEP is conservatively costed for the full 3 months of use, as a 28 day course of PEP could have been used 3 times in the period.

References

Phillips, A.N., Cambiano, V., Nakagawa, F., Brown, A.E., Lampe, F., et al. Effect on transmission of HIV-1 resistance of timing of implementation of viral load monitoring to determine switches from first to second-line antiretroviral regimens in resource-limited settings. AIDS, 2011. 25(6).

Cambiano, V., Phillips, A.N., Nakagawa, F., Brown, A.E., Lampe, F., et al. Transmission of Drug Resistant HIV and Its Potential Impact on Mortality and Treatment Outcomes in Resource-Limited Settings. The Journal of Infectious Diseases, 2013. 207(suppl_2): p. S57-S62.

Cambiano, V., Phillips, A.N., Nakagawa, F., Brown, A.E., Lampe, F., et al. Predicted levels of HIV drug resistance: potential impact of expanding diagnosis, retention, and eligibility criteria for antiretroviral therapy initiation. AIDS, 2014. 28: p. S15-S23.

CIA. The World Factbook. 2021 [cited 2021 21st January]; Available from: https://www.cia.gov/the-world-factbook/.

Fonner, V.A., Campbell, A., Chawla, M., Lissouba, P., Goh, K., et al. Voluntary counseling and testing (VCT) for changing HIV-related risk behavior in developing countries. Cochrane Database Syst Rev, 2012. 9(9): p. Cd001224.

Desmond, N., Nagelkerke, N., Lora, W., Chipeta, E., Sambo, M., et al. Measuring sexual behaviour in Malawi: a triangulation of three data collection instruments. BMC Public Health, 2018. 18:807.

Glynn, J.R., Kayuni, N., Banda, E., Parrott, F., Floyd, S., et al. Assessing the Validity of Sexual Behaviour Reports in a Whole Population Survey in Rural Malawi. PLoS ONE, 2011. 6(7): e22840.

Yeatman, S., Trinitapoli, J. Best-Friend Reports: A Tool for Measuring the Prevalence of Sensitive Behaviors. Am J Public Health, 2011. 101:1666–1667.

Gregson, S., Zhuwau, T., Nyamukapa, C.A., Garnett, G.P., Mason, P.R., et al. Methods to Reduce Social Desirability Bias in Sex Surveys in Low-Development Settings: Experience in Zimbabwe. Sexually Transmitted Diseases, 2002. 29(10): p. 568-575.

Johnson, L. and R. Dorrington, The demographic and epidemiological impact of HIV/AIDS treatment and prevention programmes: an evaluation based on the ASSA2000 model. Paper presented at the Demographic Association of Southern Africa Conference, Cape Town, 26-27 September 2002, 2002.

Hollingsworth, T.D., Anderson, R.M., Fraser, C. HIV-1 Transmission, by Stage of Infection. The Journal of Infectious Diseases, 2008. 198(5): p. 687-693.

Bellan, S.E., Bhattacharya, T., Moss, S., Mooney, T., Andrey, K., et al. Reassessment of HIV-1 Acute Phase Infectivity: Accounting for Heterogeneity and Study Design with Simulated Cohorts. PLOS Medicine, 2015. 12(3): p. e1001801.

Cohen, M.S., Chen, Y.Q., McCauley, M., Gamble, T., Hosseinipour, M.C., et al. Sexually transmitted diseases enhance HIV transmission: no longer a hypothesis. The Lancet, 1998. 351: S5-S7.

Bailey, R.C., Moses, S., Parker, C., Agot, K., Maclean, I., et al. Male circumcision for HIV prevention in young men in Kisumu, Kenya: a randomised controlled trial. Lancet, 2007. 369:643–56.

Auvert, B., Taljaard, D., Lagarde, E., Sobngwi-Tambekou, J., Sitta, R., et al. Randomized, controlled intervention trial of male circumcision for reduction of HIV infection risk: the ANRS 1265 Trial. PLoS Med, 2005. 2:e298.

Gray, R.H., Kigozi, G., Serwadda, D., Makumbi, F., Nalugoda, F., et al. The effectiveness of male circumcision for HIV prevention and effects on risk behaviors in a posttrial follow-up study. AIDS, 2012. 26:609–15.

Heffron, R., Ngure, K., Odoyo, J., Mugo, N., Mbori-Ngacha, D., et al. Pre-exposure prophylaxis for HIV-negative persons with partners living with HIV: uptake, use, and effectiveness in an open-label demonstration project in East Africa. Gates Open Research, 2018. 1:3.

Castro, H., Phillips, A.N., Smit, E., Hoepelman, A.I.M., and Bunnik, E. Persistence of HIV-1 transmitted drug resistance mutations. Journal of Infectious Diseases, 2013. 208:1459–1463.

Smith, D.M., May, S., Ganesh, S., Tovanabutra, S., and Rerks-Ngarm, S. HIV drug resistance acquired through superinfection. AIDS, 2005. 19(12): p. 1251-1256.

Corvasce, M., Martins, M., Simões, E., Carreira, D., and Almeida, D. Evidence of differential selection of HIV-1 variants carrying drug-resistant mutations in seroconverters. Antiviral Therapy, 2006. 11:329–334.

Turner, S.E., Hull, M., and Robinson, L. Diminished Representation of HIV-1 Variants Containing Select Drug Resistance–Conferring mutations in Primary HIV-1 Infection. JAIDS, 2004. 37:1627–1631.

Phillips, A.N., Cambiano, V., Nakagawa, F., Brown, A.E., Lampe, F., et al. Increased HIV Incidence in Men Who Have Sex with Men Despite High Levels of ART-Induced Viral Suppression: Analysis of an Extensively Documented Epidemic. PLoS ONE, 2013. 8(2): e55312.

Grimsrud, A., Wilkinson, L., Eshun-Wilson, I., Holmes, C., Sikazwe, I., et al. Understanding Engagement in HIV Programmes: How Health Services Can Adapt to Ensure No One Is Left Behind. Current HIV/AIDS Reports, 2020. 17:458–466.

Wanyenze, R.K., Kamya, M., Bassett, I.V., Joloba, M., and McLellan, S. Linkage to HIV care and survival following inpatient HIV counseling and testing. AIDS and Behavior, 2011. 15:751-760.

Bassett, I.V., Wong, W., Freeman, E., Gibbons, M., and Narine, R. Routine voluntary HIV testing in Durban, South Africa: the experience from an outpatient department. Journal of Acquired Immune Deficiency Syndromes, 2007. 46:181-185.

Hensen, B., Lewis, H., Mwai, P., Muganwa, M., and Molyneux, R. Universal voluntary HIV testing in antenatal care settings: a review of the contribution of provider-initiated testing & counselling. Tropical Medicine & International Health, 2012. 17:59-70.

Sabapathy, K., Van den Bergh, R., Fidler, S., Hayes, R., and Ford, N. Uptake of home-based voluntary HIV testing in sub-Saharan Africa: a systematic review and meta-analysis. PLoS Medicine, 2012. 9(12): e1001351.

Gibas, K.M., van den Berg, P., Powell, V.E., Krakower, D.S. Drug Resistance during HIV Preexposure Prophylaxis. Drugs, 2019. 79(6): 609–619.

Lehman, D.A., Baeten, J.M., McCoy, C.O., Weis, J.F., Peterson, D., et al. Risk of Drug Resistance Among Persons Acquiring HIV Within a Randomized Clinical Trial of Single- or Dual-Agent Preexposure Prophylaxis. J Infect Dis, 2015. 211(8):1211-1218.

Parikh, U.M., Mellors, J.W. Should we fear resistance from tenofovir/emtricitabine preexposure prophylaxis? Curr Opin HIV/AIDS, 2016. 11:49-55.

Landovitz, R.J., Donnell, D., Clement, M.E., Karuna, S., and Cresswell, D. Cabotegravir for HIV prevention in cisgender men and transgender women. N Engl J Med, 2021. 385(7):595-608.

Delany-Moretlwe, S., Hughes, J.P., Bock, P., Ouma, S., Hunidzarira, P., et al. Cabotegravir for prevention of HIV-1 in women: results from HPTN 084, a phase III, 2 randomised controlled trial. Lancet, 2022.

Eshleman, S.H., Fogel, J.M., Piwowar-Manning, E., Chau, G., Cummings, V., et al. Characterization of Human Immunodeficiency Virus (HIV) Infections in Women Who Received Injectable Cabotegravir or Tenofovir Disoproxil Fumarate/Emtricitabine for HIV Prevention: HPTN 084. J Infect Dis, 2022.
Eshleman S et al. CAB-LA PrEP: early detection of HIV infection may reduce INSTI resistance risk. CROI 2022, 12–16 and 22–24 February, virtual meeting. Oral abstract 95. https://www.croiconference.org/abstract/cab-la-prep-early-detection-of-hiv-infection-may-reduce-insti-resistance-risk/

Marzinke M (presenter Landovitz RJ) Laboratory analysis of HIV infections in HPTN 083: injectable CAB for PrEP. Conference on Retroviruses and Opportunistic Infections, abstract 183, 2021.
Marzinke, M.A., Grinsztejn, B., Fogel, J.M., Piwowar-Manning, E., Li, M.J., et al. Characterization of Human Immunodeficiency Virus (HIV) Infection in Cisgender Men and Transgender Women Who Have Sex With Men Receiving Injectable Cabotegravir for HIV Prevention: HPTN 083. J Infect Dis, 2021.

Radzio-Basu, J., Council, O., Cong, M.E., Ruone, S., Newton, A., et al. Drug resistance emergence in macaques administered cabotegravir long-acting for pre-exposure prophylaxis during acute SHIV infection. Nature Communications, 2019.

Phillips, A.N., Cambiano, V., Nakagawa, F., Brown, A.E., Lampe, F., et al. Outcomes from monitoring of patients on antiretroviral therapy in resource-limited settings with viral load, CD4 cell count, or clinical observation alone: a computer simulation model. The Lancet, 2008. 371:1443-1451.

Phillips, A.N., Cambiano, V., Nakagawa, F., Brown, A.E., Lampe, F., et al. Effect on transmission of HIV-1 resistance of timing of implementation of viral load monitoring to determine switches from first to second-line antiretroviral regimens in resource-limited settings. AIDS, 2011. 25:843-850.

Nakagawa, F., Phillips, A.N., Smit, E., Mortimer, P., and Mills, E. Projected life expectancy of people with HIV according to timing of diagnosis. AIDS, 2012. 26:335-343.

Nakagawa, F., Smit, E., Muir, A., Phillips, A.N., and Lundgren, J. Projected lifetime healthcare costs associated with HIV infection. PLOS ONE, 2015. 10(4): e0125018.

Pantazis N, Touloumi G. Bivariate modelling of longitudinal measurements of two human immunodeficiency type 1 disease progression markers in the presence of informative drop-outs. JRSS C. 2005;54:405–423.

Sabin CA, Devereux H, Phillips AN, Wainwright E, Loveday C, et al. Course of viral load throughout HIV-1 infection. J Acquir Immune Defic Syndr. 2000;23:172–177.

Hubert JB, Burgard M, Dussaix E, Costagliola D, Rouzioux C, et al. Natural history of serum HIV-1 RNA levels in 330 patients with known date of infection. AIDS. 2000;14:123–131.

O'Brien TR, Rosenberg PS, Yellin F, Goedert JJ, et al. Longitudinal HIV-1 RNA levels in a cohort of homosexual men. J Acquir Immune Defic Syndr. 1998;18:155–161.

Henrard DR, Phillips JF, Muenz LR, Clark WS, Gwinn M, et al. Natural history of HIV-1 cell-free viraemia. JAMA. 1995;274:554–558.

Lyles RH, Munoz A, Yamashita TE, Bazmi H, Detels R, et al. Natural history of human immunodeficiency virus type 1 viremia after seroconversion and proximal to AIDS in a large cohort of homosexual men. J Infect Dis. 2000;181:872–880.

Touloumi G, Hatzakis A, Rosenberg PS, Goedert JJ, et al. Differences in HIV RNA levels before the initiation of antiretroviral therapy among 1864 individuals with known HIV-1 seroconversion dates. AIDS. 2004;18:1697–1705.

Mellors JW, Munoz A, Giorgi JV, Margolick JB, Tassoni CJ, et al. Plasma viral load and CD4+ lymphocytes as prognostic markers of HIV-1 infection. Ann Intern Med. 1997;126:946–954.

Koot M, Keet IP, Vos AH, de Goede RE, Roos MT, et al. Prognostic value of HIV-1 syncytium-inducing phenotype for rate of CD4+ cell depletion and progression to AIDS. Ann Intern Med. 1993;118:681–688.

Darby SC, Ewart DW, Giangrande PL, Spooner RJ, Rizza CR. Importance of age at infection with HIV-1 for survival and development of AIDS in UK haemophilia population. Lancet. 1996;347(9015):1573–1579.

Fox MP, Van Cutsem G, Giddy J, Maskew M, Keiser O, et al. Rates and predictors of failure of first-line antiretroviral therapy and switch to second-line ART in South Africa. J Acquir Immune Defic Syndr. 2012;60(4):428–437.

Johnston V, Fielding KL, Charalambous S, Churchyard G, Phillips A, et al. Outcomes following virological failure and predictors of switching to second-line antiretroviral therapy in a South African treatment program. J Acquir Immune Defic Syndr. 2012;61(3):370–380.

Rohr JK, Ive P, Berhanu R, Shearer K, Maskew M, et al. Predictors of time to switch to second-line ART after first-line failure in Johannesburg, South Africa. Top Antivir Med. 2014;22:280.

Narainsamy D, Mahomed S. Delays in switching patients onto second-line antiretroviral treatment at a public hospital in eThekwini, KwaZulu-Natal. S Afr J HIV Med. 2017;18:5.

Petersen ML, Tran L, Geng EH, Reynolds SJ, Kambugu A, et al. Delayed switch of antiretroviral therapy after virologic failure associated with elevated mortality among HIV-infected adults in Africa. AIDS. 2014;28:2097–2107.

Ramadhani HO, Bartlett JA, Thielman NM, Pence BW, Kimani SM, et al. The effect of switching to second-line antiretroviral therapy on the risk of opportunistic infections among patients infected with human immunodeficiency virus in Northern Tanzania. Open Forum Infect Dis. 2016;3:ofw018.

Murphy RA, Court R, Maartens G, Sunpath H. Second-line antiretroviral therapy in sub-Saharan Africa: it is time to mind the gaps. AIDS Res Hum Retroviruses. 2017;33:1181–1184.

Cheng Y, Sauer B, Zhang Y, Nickman NA, Jamjian C, et al. Adherence and virologic outcomes among treatment-naïve veteran patients with human immunodeficiency virus type 1 infection. Medicine (Baltimore). 2018;97(2):e9430.

Fox Z, Phillips AN, Cohen C, Neuhaus J, Baxter J, et al. Viral resuppression and detection of drug resistance following interruption of a suppressive non-nucleoside reverse transcriptase inhibitor-based regimen. AIDS. 2008;22:2279–2289.
Bangsberg DR, Moss AR, Deeks SG, Aranow R, Tucker L et al. Paradoxes of adherence and drug resistance to HIV antiretroviral therapy. J Antimicrob Chemother. 2004;53(5):696–699.

Bangsberg DR, Acosta EP, Gupta R, Guzman D, Riley ED et al. Adherence-resistance relationships for protease and non-nucleoside reverse transcriptase inhibitors explained by virological fitness. AIDS. 2006;20(2):223–231. PMID: 16511415

Bangsberg DR. Less than 95% adherence to nonnucleoside reverse-transcriptase inhibitor therapy can lead to viral suppression. Clin Infect Dis. 2006;43(7):939–941. PMID: 16941380

Hamers RL, Wallis CL, Kityo C, Siwale M, Mandaliya K et al. HIV-1 drug resistance in antiretroviral-naive individuals in sub-Saharan Africa after rollout of antiretroviral therapy: a multicentre observational study. Lancet Infect Dis. 2011;11(10):750–759. PMID: 21802367

Hassan AS, Nabwera HM, Mwaringa SM, Obonyo CA, Sanders EJ et al. HIV-1 virologic failure and acquired drug resistance among first-line antiretroviral experienced adults at a rural HIV clinic in coastal Kenya: a cross-sectional study. AIDS Res Ther. 2014;11(1):9. PMID: 24456757

Hoffmann CJ, Charalambous S, Sim J, Ledwaba J, Schwikkard G et al. Viremia, resuppression, and time to resistance in HIV subtype C during first-line antiretroviral therapy in South Africa. Clin Infect Dis. 2009;49(12):1928–1935. PMID: 19911963

Hoffmann CJ, Charalambous S, Grant AD, Morris L, Churchyard GJ et al. Durable HIV RNA resuppression after virologic failure while remaining on a first-line regimen: a cohort study. Trop Med Int Health. 2014;19(2):236–239. PMID: 24588012

Kobin AB, Sheth NU. Levels of adherence required for virologic suppression among newer antiretroviral medications. Ann Pharmacother. 2011;45(3):372–379. PMID: 21386024

Li JZ, Gallien S, Ribaudo H, Heisey A, Bangsberg DR et al. Incomplete adherence to antiretroviral therapy is associated with higher levels of residual HIV-1 viremia. AIDS. 2014;28(2):181–186. PMID: 24361679

Mackie NE, Phillips AN, Kaye S, Booth C, Geretti AM. Antiretroviral drug resistance in HIV-1-infected patients with low-level viremia. J Infect Dis. 2010;201(9):1303–1307. PMID: 20350161

Rosenblum M, Deeks SG, van der Laan M, Bangsberg DR, Havlir DV et al. The risk of virologic failure decreases with duration of HIV suppression, at greater than 50% adherence to antiretroviral therapy. PLoS One. 2009;4(9):e7196. PMID: 19787058

Tran DA, Wilson DP, Shakeshaft A, Ngo AD, Doran C et al. Determinants of virological failure after 1 year's antiretroviral therapy in Vietnamese people with HIV: findings from a retrospective cohort of 13 outpatient clinics in six provinces. Sex Transm Infect. 2014;90(7):538–544. PMID: 24619575

Usitalo A, Leister E, Tassiopoulos K, Allison S, Malee K et al. Relationship between viral load and self-report measures of medication adherence among youth with perinatal HIV infection. AIDS Care. 2014;26(1):107–115. PMID: 23800360

von Wyl V, Klimkait T, Yerly S, Nicca D, Furrer H et al. Adherence as a predictor of the development of class-specific resistance mutations: the Swiss HIV Cohort Study. PLoS One. 2013;8(10):e77691. PMID: 24147057

Johannessen A, Naman E, Kivuyo SL, Kasubi MJ, Holberg-Petersen M et al. Virological efficacy and emergence of drug resistance in adults on antiretroviral treatment in rural Tanzania. BMC Infect Dis. 2009;9:108. PMID: 19583845

Musengimana G, Tuyishime E, Kiromera A, Malamba S, Mulindabigwi A et al. Acquired HIV drug resistance among adults living with HIV receiving first-line antiretroviral therapy in Rwanda: a cross-sectional nationally representative survey. Antivir Ther. 2022;27(3):1–16.

Agegnehu CD, Techane MA, Mersha AT, Atalell KA. Burden and associated factors of virological failure among people living with HIV in Sub-Saharan Africa: a systematic review and meta-analysis. AIDS Behav. 2022;26(11):3456–3469.

Muyingo SK, Walker AS, Reid A, Munderi P, Gibb DM et al. Patterns of individual and population-level adherence to antiretroviral therapy and risk factors for poor adherence in the first year of the DART trial in Uganda and Zimbabwe. J Acquir Immune Defic Syndr. 2008;48(4):468–475. PMID: 18614918

Walmsley SL, Antela A, Clumeck N, Duiculescu D, Eberhard A et al. Dolutegravir plus abacavir-lamivudine for the treatment of HIV-1 infection. N Engl J Med. 2013;369(19):1807–1818. PMID: 24195548

Walmsley S, Baumgarten A, Berenguer J, Felizarta F, Florence E et al. Dolutegravir plus abacavir/lamivudine for the treatment of HIV-1 infection in antiretroviral therapy-naive patients: week 96 and week 144 results from the SINGLE randomized clinical trial. J Acquir Immune Defic Syndr. 2015;70(5):515–519. PMID: 26262777

O'Connor JL, Gardner EM, Esser S, Mannheimer SB, Lifson AR, et al. A simple self-reported adherence tool as a predictor of viral rebound in people with viral suppression on antiretroviral therapy. HIV Med. 2016;17:124–132.

Filimão DBC, Moon TD, Senise JF, Diaz RS, Sidat M, et al. Individual factors associated with time to non-adherence to ART pick-up within HIV care and treatment services in three health facilities of Zambézia Province, Mozambique. PLoS One. 2019;14(3):e0213804.

Haberer JE, Bosco M, Bwana M, Orrell C, Asiimwe S, et al. ART adherence and viral suppression are high among most non-pregnant individuals with early-stage, asymptomatic HIV infection: an observational study from Uganda and South Africa. J Int AIDS Soc. 2019;22:e25232.

Liegeois F, Eymard-Duvernay S, Boyer S, Maradan G, Kouanfack C, et al. Heterogeneity of virological suppression in the national antiretroviral programme of Cameroon (ANRS 12288 EVOLCAM). HIV Med. 2019;20:38–46.

Jiamsakul A, Kariminia A, Althoff KN, Cesar C, Cortes CP, et al. HIV viral load suppression in adults and children receiving antiretroviral therapy—results from the IeDEA collaboration. J Acquir Immune Defic Syndr. 2017;76:319–329.

Malawi Ministry of Health. Quarterly reports. www.health.gov.mw

Population Health Impact Surveys. https://phia.icap.columbia.edu/

Chi BH, Cantrell RA, Zulu I, Mulenga LB, Levy JW, et al. Adherence to first-line antiretroviral therapy affects non-virologic outcomes among patients on treatment for more than 12 months in Lusaka, Zambia. Int J Epidemiol. 2009;38(3):746–756.

WHO HIV Drug Resistance Surveillance Report 2012. http://apps.who.int/iris/bitstream/handle/10665/75183/9789241503938_eng.pdf

Cozzi-Lepri A, UK HIV Drug Resistance, UK CHIC. Long-term probability of detecting drug-resistant HIV in treatment-naive patients initiating combination antiretroviral therapy. Clin Infect Dis. 2010;50(9):1275–1285.

Cheeseman SH, Hattox SE, McLaughlin MM, Koup RA, Andrews C, et al. Pharmacokinetics of nevirapine: initial single-rising-dose study in humans. Antimicrob Agents Chemother. 1993;37(2):178–182.

Gardner EM, Burman WJ, Steiner JF, Anderson PL, Bangsberg DR. Antiretroviral medication adherence and the development of class-specific antiretroviral resistance. AIDS. 2009;23(9):1035–1046.

Gross R, Bilker WB, Wang H, Chapman J. How long is the window of opportunity between adherence failure and virologic failure on efavirenz-based HAART? HIV Clin Trials. 2008;9(3):202–206.

Meresse M, March L, Kouanfack C, Bonono RC, Boyer S, et al. Patterns of adherence to antiretroviral therapy and HIV drug resistance over time in the Stratall ANRS 12110/ESTHER trial in Cameroon. HIV Med. 2014;15(8):478–487.

Parienti JJ, Massari V, Reliquet V, Chaillot F, Le MG, et al. Effect of twice-daily nevirapine on adherence in HIV-1-infected patients: a randomized controlled study. AIDS. 2007;21(16):2217–2222.

Hill A, McBride A, Sawyer AW, Clumeck N, Gupta RK. Resistance at virological failure using boosted protease inhibitors versus nonnucleoside reverse transcriptase inhibitors as first-line antiretroviral therapy—implications for sustained efficacy of ART in resource-limited settings. J Infect Dis. 2013;207 Suppl 2:S78–S84.

Orrell C, Harling G, Lawn SD, Kaplan R, McNally M, et al. Conservation of first-line antiretroviral treatment regimen where therapeutic options are limited. Antivir Ther. 2007;12(1):83–88.

Rutstein SE, Hosseinipour MC, Kamwendo D, Soko A, Mkandawire M, et al. Dried blood spots for viral load monitoring in Malawi: feasible and effective. PLoS One. 2015;10(4):e0124748.

Bonner K, Mezochow A, Roberts T, Ford N, Cohn J. Viral load monitoring as a tool to reinforce adherence: a systematic review. J Acquir Immune Defic Syndr. 2013;64(1):74–78.

Bärnighausen T, Chaiyachati K, Chimbindi N, Haberer JE, Newell ML, et al. Interventions to increase antiretroviral adherence in sub-Saharan Africa: a systematic review of evaluation studies. Lancet Infect Dis. 2011;11:942–951.

Kranzer K, Ford N. Unstructured treatment interruption of antiretroviral therapy in clinical practice: a systematic review. Trop Med Int Health. 2011;16(10):1297–1313.

Kranzer K, Lewis JJ, Ford N, Zeinecker J, Orrell C, et al. Treatment interruption in a primary care antiretroviral therapy program in South Africa: cohort analysis of trends and risk factors. J Acquir Immune Defic Syndr. 2010;55(3):e17–e23.

Tassie JM, Baijal P, Vitoria MA, Alisalad A, Crowley SP, et al. Trends in retention on antiretroviral therapy in national programs in low-income and middle-income countries. J Acquir Immune Defic Syndr. 2010;54(4):437–441.

Wandeler G, Keiser O, Pfeiffer K, Pestilli S, Fritz C, et al. Outcomes of antiretroviral treatment programs in rural Southern Africa. J Acquir Immune Defic Syndr. 2012;59(2):e9–e16.

Dear N, Esber A, Iroezindu M, Bahemana E, Kibuuka H, et al. Routine HIV clinic visit adherence in the African Cohort Study. AIDS Res Ther. 2022;19(1):1–10.

Wallis CL, Mellors JW, Venter WD, Sanne I, Stevens W. Varied patterns of HIV-1 drug resistance on failing first-line antiretroviral therapy in South Africa. J Acquir Immune Defic Syndr. 2010;53(4):480–484.

McMahon JH, Elliott JH, Bertagnolio S, Kubiak R, Jordan MR. Viral suppression after 12 months of antiretroviral therapy in low- and middle-income countries: a systematic review. Bull World Health Organ. 2013;91(5):377–385.

Charurat M, Oyegunle M, Benjamin R, Habib A, Eze E, et al. Patient retention and adherence to antiretrovirals in a large antiretroviral therapy program in Nigeria: a longitudinal analysis for risk factors. PLoS One. 2010;5(5):e10584.

DeGruttola V, Dix L, D'Aquila R, Holder D, Phillips A, et al. The relation between baseline HIV drug resistance and response to antiretroviral therapy: re-analysis of retrospective and prospective studies using a standardized data analysis plan. Antivir Ther. 2000;5(1):41–48.

Grinsztejn B, Nguyen BY, Katlama C, Gatell JM, Lazzarin A, et al. Safety and efficacy of the HIV-1 integrase inhibitor raltegravir (MK-0518) in treatment-experienced patients with multidrug-resistant virus: a phase II randomised controlled trial. Lancet. 2007;369(9569):1261–1269.

Paton N, Kityo C, Thompson J, Nankya I, Bagenda L, et al. Nucleoside reverse-transcriptase inhibitor cross-resistance and outcomes from second-line antiretroviral therapy in the public health approach: an observational analysis within the randomised, open-label, EARNEST trial. Lancet HIV. 2017;4(8):e341–e348.

Rosenbloom DI, Hill AL, Rabi SA, Siliciano RF, Nowak MA. Antiretroviral dynamics determines HIV evolution and predicts therapy outcome. Nat Med. 2012;18(9):1378–1385.

Genberg BL, Wilson IB, Bangsberg DR, Arnsten J, Goggin K, et al. Patterns of antiretroviral therapy adherence and impact on HIV RNA among patients in North America. AIDS. 2012;26(11):1415–1423.

Cambiano V, Lampe FC, Rodger AJ, Smith CJ, Geretti AM, et al. Use of a prescription-based measure of antiretroviral therapy adherence to predict viral rebound in HIV-infected individuals with viral suppression. HIV Med. 2010;11(3):216–224.

Arnsten JH, Demas PA, Farzadegan H, Grant RW, Gourevitch MN, et al. Antiretroviral therapy adherence and viral suppression in HIV-infected drug users: comparison of self-report and electronic monitoring. Clin Infect Dis. 2001;33(8):1417–1423.

Montaner JS, Reiss P, Cooper D, Vella S, Harris M, et al. A randomized, double-blind trial comparing combinations of nevirapine, didanosine, and zidovudine for HIV-infected patients: the INCAS Trial. JAMA. 1998;279(12):930–937.

Eron JJ, Benoit SL, Jemsek J, MacArthur RD, Santana J, et al. Treatment with lamivudine, zidovudine, or both in HIV-positive patients with 200 to 500 CD4+ cells per cubic millimeter. N Engl J Med. 1995;333(25):1662–1669.

Havlir D, McLaughlin MM, Richman DD. A pilot study to evaluate the development of resistance to nevirapine in asymptomatic HIV-infected patients with CD4 cell counts of >500/mm^3: AIDS Clinical Trials Group Protocol 208. J Infect Dis. 1995;172(5):1379–1383.

Kuritzkes DR, Quinn JB, Benoit SL, Shugarts DL, Griffin A, et al. Drug resistance and virologic response in NUCA 3001, a randomized trial of lamivudine (3TC) versus zidovudine (ZDV) versus ZDV plus 3TC in previously untreated patients. AIDS. 1996;10(9):975–981.

Larder BA. Viral resistance and the selection of antiretroviral combinations. J Acquir Immune Defic Syndr Hum Retrovirol. 1995;10 Suppl 1:S28–S33.

Phillips AN, Eron J, Bartlett J, Kuritzkes DR, Johnson VA, et al. Correspondence between the effect of zidovudine plus lamivudine on plasma HIV level/CD4 lymphocyte count and the incidence of clinical disease in infected individuals. AIDS. 1997;11(2):169–175.

Wittkop L, Gunthard HF, de Wolf F, Dunn D, Cozzi-Lepri A, et al. Effect of transmitted drug resistance on virological and immunological response to initial combination antiretroviral therapy for HIV (EuroCoord-CHAIN joint project): a European multicohort study. Lancet Infect Dis. 2011;11(5):363–371.

Wittkop L, Bitard J, Lazaro E, Neau D, Bonnet F, et al. Effect of cytomegalovirus-induced immune response, self antigen-induced immune response, and microbial translocation on chronic immune activation in successfully treated HIV type 1-infected patients: the ANRS CO3 Aquitaine Cohort. J Infect Dis. 2013;207(4):622–627.

Gallant JE, Staszewski S, Pozniak AL, DeJesus E, Suleiman JM, et al. Efficacy and safety of tenofovir DF vs stavudine in combination therapy in antiretroviral-naive patients: a 3-year randomized trial. JAMA. 2004;292(2):191–201.

Harrigan PR, Hogg RS, Dong WW, Yip B, Wynhoven B, et al. Predictors of HIV drug-resistance mutations in a large antiretroviral-naive cohort initiating triple antiretroviral therapy. J Infect Dis. 2005;191(3):339–347.

Ledergerber B, Egger M, Opravil M, Telenti A, Hirschel B, et al. Clinical progression and virological failure on highly active antiretroviral therapy in HIV-1 patients: a prospective cohort study. Lancet. 1999;353(9156):863–868.

Phillips AN, Staszewski S, Weber R, Kirk O, Francioli P, et al. HIV viral load response to antiretroviral therapy according to the baseline CD4 cell count and viral load. JAMA. 2001;286(20):2560–2567.

Phillips AN, Dunn D, Sabin C, Pozniak A, Matthias R, et al. Long term probability of detection of HIV-1 drug resistance after starting antiretroviral therapy in routine clinical practice. AIDS. 2005;19(5):487–494.

Staszewski S, Miller V, Sabin C, Carlebach A, Berger AM, et al. Virological response to protease inhibitor therapy in an HIV clinic cohort. AIDS. 1999;13(3):367–373.

Staszewski S, Miller V, Sabin C, Schlecht C, Gute P, et al. Determinants of sustainable CD4 lymphocyte count increases in response to antiretroviral therapy. AIDS. 1999;13(8):951–956.

Van Leth LF, Phanuphak P, Ruxrungtham K, Baraldi E, Miller S, et al. Comparison of first-line antiretroviral therapy with regimens including nevirapine, efavirenz, or both drugs, plus stavudine and lamivudine: a randomised open-label trial, the 2NN Study. Lancet. 2004;363(9417):1253–1263.

Cambiano V, Bertagnolio S, Jordan M, Pillay D, Perriens J, et al. Predicted levels of HIV drug resistance: potential impact of expanding diagnosis, retention, and eligibility criteria for antiretroviral therapy initiation. AIDS. 2014;28 Suppl 1:S15–S23.

Ruggiero A, Cozzi-Lepri A, Beloukas A, Richman D, Khoo S, et al. Factors associated with persistence of plasma HIV-1 RNA during long-term continuously suppressive first-line antiretroviral therapy. Open Forum Infect Dis. 2018;5(2):ofy032.

Gross R, Bilker WB, Friedman HM, Strom BL. Effect of adherence to newly initiated antiretroviral therapy on plasma viral load. AIDS. 2001;15(16):2109–2117.

Ledergerber B, Lundgren JD, Walker AS, Sabin C, Justice A, et al. Predictors of trend in CD4-positive T-cell count and mortality among HIV-1-infected individuals with virological failure to all three antiretroviral-drug classes. Lancet. 2004;364(9428):51–62.

Mocroft A, Phillips AN, Gatell J, Ledergerber B, Fisher M, et al. Normalisation of CD4 counts in patients with HIV-1 infection and maximum virological suppression who are taking combination antiretroviral therapy: an observational cohort study. Lancet. 2007;370:407–413.

Bishop J, DeShields S, Cunningham T, Troy SB. CD4 count recovery after initiation of antiretroviral therapy in patients infected with human immunodeficiency virus. Am J Med Sci. 2016;352(3):239–244.

Geng E, Neilands T, Thiebaut R, Bosco Bwana M, Nash D, et al. CD4 T cell recovery during suppression of HIV replication: an international comparison of the immunological efficacy of antiretroviral therapy in North America, Asia and Africa. Int J Epidemiol. 2015;44(1):251–263.

O'Connor J, Smith CJ, Lampe FC, Hill T, Gompels M, et al. Failure to achieve a CD4 cell count response on combination antiretroviral therapy despite consistent viral load suppression. AIDS. 2014;28:919–924.

d'Arminio Monforte A, Cozzi-Lepri A, Phillips AN, De Luca A, Murri R, et al. Interruption of HAART in HIV clinical practice: results from the ICONA study. J Acquir Immune Defic Syndr. 2005;38:407–416.

Li X, Margolick JB, Conover CS, Badri S, Riddler SA, et al. Interruption and discontinuation of HAART in the MACS. J Acquir Immune Defic Syndr. 2005;38(3):320–328.

Mocroft A, Youle M, Moore A, Sabin CA, Madge S, et al. Reasons for modification and discontinuation of antiretrovirals: results from a single treatment centre. AIDS. 2001;15(2):185–194.

Wit FWNM, Blanckenberg DH, Brinkman K, Prins JM, van der Ende ME, et al. Safety of long-term interruption of successful antiretroviral therapy: the ATHENA cohort study. AIDS. 2005;19(3):345–348.

Sigaloff KC, Ramatsebe T, Viana R, de Wit TF, Wallis CL, et al. Accumulation of HIV drug resistance mutations in patients failing first-line antiretroviral treatment in South Africa. AIDS Res Hum Retroviruses. 2012;28(2):171–175.

Deeks SG, Grant RM, Wrin T, Paxinos EE, Liegler T, et al. Persistence of drug-resistant HIV-1 after a structured treatment interruption and its impact on treatment response. AIDS. 2003;17(3):361–370.

Devereux HL, Youle M, Johnson MA, Loveday C, Wainwright E, et al. Rapid decline in detectability of HIV-1 drug resistance mutations after stopping therapy. AIDS. 1999;13:F123–F127.

Devereux HL, Emery VC, Johnson MA, Loveday C, Wainwright E, et al. Replicative fitness in vivo of HIV-1 variants with multiple drug resistance associated mutations. J Med Virol. 2001;65:218–224.

Hance AJ, Lemiale V, Izopet J, Pellegrin I, Massip P, et al. Changes in HIV-1 populations after treatment interruption in patients failing antiretroviral therapy. J Virol. 2001;75:6410–6417.

Tarwater PM, Parish M, Gallant JE. Prolonged treatment interruption after immunologic response to HAART. Clin Infect Dis. 2003;37:1541–1548.

Walter H, Low P, Harrer T, Holfelder H, Jaeger H, et al. No evidence for persistence of multidrug resistant viral strains after a 7-month treatment interruption in an HIV-1 infected individual. J Acquir Immune Defic Syndr. 2002;31:137–146.

Phillips A, CASCADE Collaboration. Short-term risk of AIDS according to current CD4 cell count and viral load in antiretroviral drug-naïve individuals and those treated in the monotherapy era. AIDS. 2004;18(1):51–58.

Sifuna P, Otieno L, Ogwang S, Ogutu B, Andagalu B, et al. Cause-specific mortality in the Kombewa health and demographic surveillance systems site, rural Western Kenya from 2011–2015. Glob Health Action. 2018;11(1):1442959.

UN. 2019 Revision of World Population Prospects. United Nations Department of Economic and Social Affairs, Population Division.

Vandepitte J, Lyerla J, Dallabetta G, Crabbe F, Alary M, et al. Estimates of the number of female sex workers in different regions of the world. Sex Transm Infect. 2006;82(Suppl III):iii18–iii25.

Cowan FM, Davey CB, Fearon E, Mushati P, Dirawo J, Cambiano V, et al. The HIV care cascade among female sex workers in Zimbabwe: results of a population-based survey from the Sisters Antiretroviral Therapy Programme for Prevention of HIV, an integrated response (SAPPH-IRe) trial. J Acquir Immune Defic Syndr. 2017;74:375–382.

Cowan FM, editor. Getting to precision – Female Sex Worker population size estimates in Zimbabwe. 22nd International Conference on AIDS and STIs in Africa (ICASA); 2023; Harare, Zimbabwe.

Frascino NY, Edwards JK, Herce ME, Maselko J, Pettifor AE, Mbeye N, et al. Differences in access to HIV services and risky sexual behaviors among Malawian women at social venues who do and do not engage in sex work. AIDS Behav. 2021;25(9):2897–2907.

Lancaster KE, Cernigliaro D, Zulliger R, Fleming PF. HIV care and treatment experiences among female sex workers living with HIV in sub-Saharan Africa: a systematic review. Afr J AIDS Res. 2016;15(4):377–386.

Cowan FM, Chabata ST, Musemburi S, Fearon E, Davey C, Ndori-Mharadze T, et al. Strengthening the scale-up and uptake of effective interventions for sex workers for population impact in Zimbabwe. J Int AIDS Soc. 2019;22 Suppl 4:e25320.

Fearon E, Chabata ST, Magutshwa S, Ndori-Mharadze T, Musemburi S, Chidawanyika H, et al. Estimating the population size of female sex workers in Zimbabwe: comparison of estimates obtained using different methods in twenty sites and development of a national-level estimate. J Acquir Immune Defic Syndr. 2020;85(1):30–38.

Gregson S, Garnett GP, Nyamukapa CA, Hallett TB, Lewis JJ, et al. HIV decline in Zimbabwe due to reductions in risky sex? Evidence from a comprehensive epidemiological review. Int J Epidemiol. 2010;39(5):1311–1323.

Halperin DT, Mugurungi O, Hallett TB, Muchini B, Campbell B, et al. A surprising prevention success: why did the HIV epidemic decline in Zimbabwe? PLoS Med. 2011;8(2):e1000414.

Mishra S. Using mathematical models to characterize HIV epidemics for the design of HIV prevention strategies. Imperial College London. 2015.

Donnell D, Haberer JE, Celum C, Baeten JM, Mugo NR, et al. Incorporating oral PrEP into standard prevention services for South African women: a nested interrupted time-series study. Lancet HIV. 2021;8:e495–e501.

Koss CA, Charlebois ED, Ayieko J, Kwarisiima D, Kabami J, et al. Uptake, engagement, and adherence to pre-exposure prophylaxis offered after population HIV testing in rural Kenya and Uganda: 72-week interim analysis of observational data from the SEARCH study. Lancet HIV. 2020;7:e245–e254.

Koss CA, Havlir DV, Ayieko J, Kwarisiima D, Kabami J, Chamie G, et al. HIV incidence after pre-exposure prophylaxis initiation among women and men at elevated HIV risk: a population-based study in rural Kenya and Uganda. PLoS Med. 2021;18(2):e1003492.

Yun K, Xu JJ, Zhang J, Zhang J, Hu Q, et al. Female and younger subjects have lower adherence in PrEP trials: a meta-analysis with implications for the uptake of PrEP service to prevent HIV. Sex Transm Infect. 2018;94:163–168.

Cong M, Mitchell J, Sweeney E, Bachman S, Hanson DL, Heneine W, et al. Prophylactic efficacy of oral emtricitabine and tenofovir disoproxil fumarate combination therapy against a tenofovir-resistant simian/human immunodeficiency virus containing the K65R mutation in macaques. J Infect Dis. 2013;208:463–467.

Landovitz RJ, Li S, Eron JJ, Grinsztejn B, Dawood H, Liu AY, et al. Tail-phase safety, tolerability, and pharmacokinetics of long-acting injectable cabotegravir in HIV-uninfected adults: a secondary analysis of the HPTN 077 trial. Lancet HIV. 2020;7:e472–e481.

Masson L, Passmore JA, Liebenberg LJ, Werner L, Baxter C, et al. Genital inflammation and the risk of HIV acquisition in women. Clin Infect Dis. 2015;61(2):260–269.

Nicolosi A, Leite ML, Musicco M, Molinari S, Lazzarin A, et al. The efficiency of male-to-female and female-to-male sexual transmission of the human immunodeficiency virus: a study of 730 stable couples. Epidemiology. 1994;5(6):606–612.

Taylor D, Durigon M, Davis H, Archibald C, Konrad B, et al. Probability of a false-negative HIV antibody test result during the window period: a tool for pre- and post-test counselling. Int J STD AIDS. 2014;26(4):215–224.

Ndashimye E, Li Y, Reyes PS, Avino M, Olabode AS, et al. High-level resistance to bictegravir and cabotegravir in subtype A- and D-infected HIV-1 patients failing raltegravir with multiple resistance mutations. J Antimicrob Chemother. 2021;76:2965–2974.

Bailey AJ, Rhee SY, Shafer RW. Integrase strand transfer inhibitor resistance in integrase strand transfer inhibitor-naive persons. AIDS Res Hum Retroviruses. 2021;37:736–743.

Rosen S, Fox MP. Retention in HIV care between testing and treatment in sub-Saharan Africa: a systematic review. PLoS Med. 2011;8:e1001056.

Jain V, Sucupira MC, Bacchetti P, Hartogensis W, Diaz RS, et al. Differential persistence of transmitted HIV-1 drug resistance mutation classes. J Infect Dis. 2011;203(8):1174–1181.

Yang WL, Kouyos RD, Böni J, Yerly S, Klimkait T, et al. Persistence of transmitted HIV-1 drug resistance mutations associated with fitness costs and viral genetic backgrounds. PLoS Pathog. 2015;11(3):e1004722.

Phillips A, Cambiano V, Revill P, Miners A, Nakagawa F, et al. Point-of-care viral load testing for sub-Saharan Africa: informing a target product profile. Open Forum Infect Dis. 2016;3(3):ofw161.

Venter WDF, Moorhouse M, Sokhela S, Fairlie L, Mashabane N, et al. Dolutegravir plus two different prodrugs of tenofovir to treat HIV. N Engl J Med. 2019;381:803–815.

Zash R, Holmes L, Diseko M, Mayondi GK, Mmalane M, et al. Neural-tube defects and antiretroviral treatment regimens in Botswana. N Engl J Med. 2019;381:827–840.

Phillips AN. Understanding the extent of excess risk of NCDs and death in people with HIV suppression on ART: an epidemiologic perspective. UNAIDS and New York Academy symposium on HIV and NCDs. 2016 May 26.

Hakim J, Musiime V, Szubert AJ, Mallewa J, Siika A, et al. Enhanced prophylaxis plus antiretroviral therapy for advanced HIV infection in Africa. N Engl J Med. 2017;377:233–245.

Mfinanga S, Chanda D, Kivuyo SL, Nyirenda S, Ngowi BJ, et al. Cryptococcal meningitis screening and community-based early adherence support in people with advanced HIV infection starting antiretroviral therapy in Tanzania and Zambia: an open-label, randomised controlled trial. Lancet. 2015;385:2173–2182.

Heestermans T, Browne JL, Aitken SC, Vervoort SC, Klipstein-Grobusch K. Determinants of adherence to antiretroviral therapy among HIV-positive adults in sub-Saharan Africa: a systematic review. BMJ Glob Health. 2016;1:e000125.

McMahon JH, Spelman T, Ford N, Greig J, Mesic A, et al. Risk factors for unstructured treatment interruptions and association with survival in low to middle income countries. AIDS Res Ther. 2016;13:25.

Agbaji OO, Abah IO, Falang KD, Ebonyi AO, Musa J, et al. Treatment discontinuation in adult HIV-infected patients on first-line antiretroviral therapy in Nigeria. Curr HIV Res. 2015;13:184–192.

TenoRes Study Group. Global epidemiology of drug resistance after failure of WHO recommended first-line regimens for adult HIV-1 infection: a multicentre retrospective cohort study. Lancet Infect Dis. 2016;16:565–575.

Msyamboza KP, Ngwira B, Dzowela T, Mvula C, Kathyola D, et al. The burden of selected chronic non-communicable diseases and their risk factors in Malawi: nationwide STEPS survey. PLoS One. 2011;6(5):e20316.

Thorogood M, Connor M, Tollman S, Lewando Hundt G, Fowkes G, et al. A cross-sectional study of vascular risk factors in a rural South African population: data from the Southern African Stroke Prevention Initiative (SASPI). BMC Public Health. 2007;7:326.

Berrington de Gonzalez A, Hartge P, Cerhan JR, Flint AJ, Hannan L, et al. Body-mass index and mortality among 1.46 million white adults. N Engl J Med. 2010;363(23):2211–2219.

Flegal KM, Kit BK, Orpana H, Graubard BI. Association of all-cause mortality with overweight and obesity using standard body mass index categories: a systematic review and meta-analysis. JAMA. 2013;309(1):71–82.

Achhra AC, Sabin CA, Ryom L, Hatleberg C, d'Arminio Monforte A, et al. Body mass index and the risk of serious non-AIDS events and all-cause mortality in treated HIV-positive individuals: D:A:D cohort analysis. J Acquir Immune Defic Syndr. 2018;78(5):579–588.

Kivimäki M, Kuosma E, Ferrie JF, Luukkonen R, Nyberg ST, et al. Overweight, obesity, and risk of cardiometabolic multimorbidity: pooled analysis of individual-level data for 120,813 adults from 16 cohort studies from the USA and Europe. Lancet Public Health. 2017;2:e277–e285.

Kanters S, et al. Meta-analysis of association between baseline NNRTI resistance and virologic failure prepared for WHO guidelines meeting. 2019.

Fournier AL, Hocqueloux L, Braun DL, Metzner KJ, Kouyos RD, et al. Dolutegravir monotherapy as maintenance strategy: a meta-analysis of individual participant data from randomized controlled trials. Open Forum Infect Dis. 2022;9(6):ofac107.

Mbhele N, Chimukangara B, Gordon M. HIV-1 integrase strand transfer inhibitors: a review of current drugs, recent advances and drug resistance. Int J Antimicrob Agents. 2021;58(6):106343.

Rossetti B, Fabbiani M, Di Carlo D, Incardona F, Abecasis A, et al. Effectiveness of integrase strand transfer inhibitors in HIV-infected treatment-experienced individuals across Europe. HIV Med. 2022;23(9):932–940.

Oliveira RI, Anstett K, Mésplède T, Routy JP, Robbins MA, et al. Selective resistance profiles emerging in patient-derived clinical isolates with cabotegravir, bictegravir, dolutegravir, and elvitegravir. Retrovirology. 2018;15:56.

Cresswell J, Campbell OMR, De Silva MJ, Filippi V. Effect of maternal obesity on neonatal death in sub-Saharan Africa: multivariable analysis of 27 national datasets. Lancet. 2012;380:1325–1330.

Cowan FM, Davey C, Fearon E, Mushati P, Dirawo J, et al. Targeted combination prevention to support female sex workers in Zimbabwe accessing and adhering to antiretrovirals for treatment and prevention of HIV (SAPPH-IRe): a cluster-randomised trial. Lancet HIV. 2018;5(8):e417–e426.

Wilson D. HIV programs for sex workers: lessons and challenges for developing and delivering programs. PLoS Med. 2015;12(6):e1001808.

Salomon JA, Vos T, Hogan DR, Gagnon M, Naghavi M, et al. Common values in assessing health outcomes from disease and injury: disability weights measurement study for the Global Burden of Disease Study 2010. Lancet. 2012;380:2129–2143.

CHAI update 2022. Personal communication.

Eaton JW, Menzies NA, Stover J, Cambiano V, Chindelevitch L, et al. Health benefits, costs, and cost-effectiveness of earlier eligibility for adult antiretroviral therapy and expanded treatment coverage: a combined analysis of 12 mathematical models. Lancet Glob Health. 2014;2(1):e23–e34.

Hyle EP, Jani IV, Lehe J, Su AE, Wood R, et al. The clinical and economic impact of point-of-care CD4 testing in Mozambique and other resource-limited settings: a cost-effectiveness analysis. PLoS Med. 2014;11(9):e1001725.

Keebler D, Revill P, Braithwaite S, Phillips A, Cambiano V, et al. How should HIV programmes monitor adults on ART? A combined analysis of three mathematical models. Lancet Glob Health. 2014;2(1):e35–e43.

Global Fund Releases. 2015. New approach on HIV viral load testing. http://www.theglobalfund.org/en/mediacenter/newsreleases/2015-06-10_New_Approach_on_HIV_Viral_Load_Testing/

Siapka M, Remme M, Dayo Obure C, Maier C, Dehne KL, et al. Is there scope for cost savings and efficiency gains in HIV services? A systematic review of the evidence from low- and middle-income countries. Bull World Health Organ. 2014;92:499–511.

Tagar E, Sundaram M, Condliffe K, Matatiyo B, Chimbwandira F, et al. Multi-country analysis of treatment costs for HIV/AIDS (MATCH): facility-level ART unit cost analysis in Ethiopia, Malawi, Rwanda, South Africa and Zambia. PLoS One. 2014;9(11):e108304.

Menzies NA, Berruti AA, Blandford JM. The determinants of HIV treatment costs in resource-limited settings. PLoS One. 2012;7(11):e48726.

Jamieson L, Gomez GB, Rebed K, Brown B, Subedar H, et al. The impact of self-selection based on HIV risk on the cost-effectiveness of preexposure prophylaxis in South Africa. AIDS. 2020;

Pretorius C, Schnure M, Dent J, Glaubius R, Mahiane G, et al. Modelling impact and cost-effectiveness of oral pre-exposure prophylaxis in 13 low-resource countries. J Int AIDS Soc. 2020;23(2):e25451.

Roberts DA, Barnabas RV, Abuna F, Lagat H, Kinuthia J, et al. The role of costing in the introduction and scale-up of HIV pre-exposure prophylaxis: evidence from integrating PrEP into routine maternal and child health and family planning clinics in western Kenya. J Int AIDS Soc. 2019;22 Suppl 4:e25296.

Nichols BE, Cele R, Jamieson L, Long LC, Siwale Z, Banda P. Community-based delivery of HIV treatment in Zambia: costs and outcomes. AIDS. 2021;35(2):299–306.

Nichols BE, Cele R, Lekodeba N, Tukei B, Ngorima-Mabhena N, Tiam A, et al. Economic evaluation of differentiated service delivery models for HIV treatment in Lesotho: costs to providers and patients. J Int AIDS Soc. 2021;24(4):e25692.

Irvine C, Egan KJ, Shubber Z, Van Rompay KK, Beanland RL et al. Efficacy of HIV Postexposure Prophylaxis: Systematic Review and Meta-analysis of Nonhuman Primate Studies. Clin Infect Dis. 2015 Jun 1;60 Suppl 3:S165-9

Massud I, Ruone S, Zlotorzynska M, Haaland R, Mills P et al. Single oral dose for HIV pre or post-exposure prophylaxis: user desirability and biological efficacy in macaques. EBioMedicine. 2020 Aug;58:102894.

Sereti I, Sheikh V, Shaffer D, Phanuphak N, Gabriel E et al. Prospective International Study of Incidence and Predictors of Immune Reconstitution Inflammatory Syndrome

and Death in People Living With Human Immunodeficiency Virus and Severe Lymphopenia. Clin Infect Dis. 2020 Jul 27;71(3):652-660

Emily P Hyle, Thulani Maphosa, Ajay Rangaraj, Mary Feser, Geoffrey C Singini Clinical impact and cost-effectiveness of the WHO-recommended advanced HIV disease package of care. Lancet Glob Health. 2025 Aug;13(8):e1436-e1447
